# Physical activity promotion in physical therapy, exercise therapy and other movement-based therapies: a scoping review and content analysis of intervention studies and theoretical works

**DOI:** 10.1186/s12966-025-01772-1

**Published:** 2025-06-10

**Authors:** Leon Matting, Klaus Pfeifer, Gorden Sudeck, Andrés Jung, Florian Langhirt, Wolfgang Geidl

**Affiliations:** 1 Education & Health Research, Institute of Sports Science, Faculty of Economics and Social Sciences, University of Tübingen, Wächterstraße 67, Tübingen, 72074 Germany; 2 Department of Sport Science and Sport, Friedrich-Alexander-Universität Erlangen-Nürnberg, Gebbertstraße 123b, 91058 Erlangen, Germany

**Keywords:** Physical activity promotion, Movement-based therapy, Behavior change, Exercise therapy, Non-communicable diseases, Behavior change techniques

## Abstract

**Background:**

Movement-based therapists, including physical, exercise, and sport therapists, play a key role in promoting physical activity in individuals with non-communicable diseases. However, no clear consensus exists on effective intervention approaches. This scoping review examines available intervention studies and theoretical works for physical activity promotion in movement-based therapy.

**Methods:**

In accordance with Colquhoun et al.‘s framework and PRISMA-ScR guidelines, we systematically searched PubMed, Scopus, Web of Science, and PsycINFO until March 31, 2024. Eligible records described physical activity-promoting concepts including interventional studies and theoretical works applicable in movement-based therapies for individuals with non-communicable diseases. Data extraction covered assessment, therapeutic content, didactic-methodological principles, and theoretical underpinnings. Interventions were categorized based on behavior change techniques (BCTs), the behavior change wheel, and a clinical reasoning model for clients behavior change. Network analysis explored relationships between therapeutic content and didactic-methodological principles.

**Results:**

Fifty-seven records met inclusion criteria; 77% were intervention studies, and 23% were theoretical works. Most concepts originated from orthopedics/rheumatology (23%), neurology (21%), and oncology (9%), while 12% were generic concepts. Across concepts, 66 biopsychosocial assessment instruments and 60 BCTs were applied (Median BCTs per concept: 11.5, range: 4–37). Key didactic-methodological principles included tailoring/individualization (*n* = 47), active participation (*n* = 39), collaborative communication (*n* = 21), and patient self-responsibility and independence (*n* = 14). Least mentioned was facilitating positive movement experiences and enjoyment of physical activity (*n* = 3). Network analysis identified action planning, goal setting, and feedback as central BCTs.

**Conclusion:**

This review provides an overview of 57 physical activity promotion concepts used in movement-based therapies for individuals with non-communicable diseases. Findings reveal considerable heterogeneity, highlighting diverse strategies used by movement-based therapists to influence physical activity behavior.

**Trial registration:**

Open Science Framework (OSF), December 23, 2022 (DOI: 10.17605/OSF.IO/AXZSJ).

**Supplementary Information:**

The online version contains supplementary material available at 10.1186/s12966-025-01772-1.

## Background

Physical activity promotion (PAP) through healthcare professionals is a crucial strategy for increasing population-level physical activity [1]. While all healthcare professionals can contribute to supporting inactive individuals in becoming more active [2], movement-based therapists—including physical therapists, physiotherapists, exercise therapists, exercise physiologists, kinesiologists, and sport therapists—are particularly well-positioned to lead these efforts due to their frequent and sustained contact with inactive individuals and their expertise in the field of physical activity and health.

Movement-based therapists and the therapies they provide are often described using various terms. In this context, we use the term *movement-based therapists* to refer to a group of health care professionals who share fundamental commonalities. From a biomedical perspective, their primary goal is to improve body functions and structures to enhance the functional capacity for independent mobility, for coping with the movement demands of everyday life or for participating in sports. From a biopsychosocial perspective, it is also important to enhance well-being and promote physical activity as one aspect of participation in various life domains (e.g., family, work, and leisure-time including exercise and sports settings). The emphasis on each perspective may vary depending on the specific profession and area of practice [3–5]. Despite differences in specialization and approach, all movement-based therapists use physical activity as the core therapeutic measure in their practice. Their interventions are rooted in disciplines such as anatomy, biomechanics, exercise physiology, kinesiology, clinical pathology, and behavioral science [6–8]. Collectively, their therapies fall under the umbrella of *movement-based therapy*, which includes physical therapy, physiotherapy, exercise therapy, and sport therapy.

Movement-based therapists not only consider the promotion of physical activity as a core aspect of their role [9] but also have regular and extended interactions with physically inactive populations. A large proportion of the individuals they treat have non-communicable diseases, among whom physical inactivity is particularly prevalent [10]. In addition, their expertise in the relationship between physical activity and health enables them to foster positive physical activity experiences in practice. Overall, this enables them to design and deliver safe, effective physical activity programs [11, 12].

Though movement-based therapists are in an ideal position for promoting physical activity, this is still a relatively recent focus within the field. Traditionally, physical, exercise, and sports therapists have emphasized improvements in physical functioning [13]. However, over the past 15 years, professional associations have increasingly advocated for integrating health behavior change, particularly PAP, as a core component of movement-based therapies [14]. This shift has led to the development of diverse PAP approaches, incorporating coaching, patient education, tailored interventions, attention to individual preferences and motivations, and self-monitoring to track physical activities [11, 12, 15, 16].

These approaches are underpinned by key principles, such as a biopsychosocial perspective and patient-centered methodologies. Despite progress in this area, however, there is no consensus on the best PAP concepts for movement-based therapists to promote healthier lifestyles. Here, we understand a PAP concept as a structured idea or approach that guides therapists in their interventional efforts to promote physically active lifestyles by outlining key principles, contents and methods. PAP concepts appear in both practical intervention studies and theoretical work. In 2018, Lowe et al. conducted an scoping review, providing a brief overview of PAP by physical therapists [17]. The aim of this scoping review is to expand on their work by offering a broader, more detailed exploration of PAP for movement-based therapists. Specifically, we aim to include all types of movement-based therapists, focus exclusively on their treatment of individuals with non-communicable diseases, and delve deeper into the content and principles of these PAP concepts. Our primary research question is: What concepts are available for movement-based therapists (e.g., physical, exercise, and sport therapists) to promote physical activity in individuals with non-communicable diseases? Further guiding questions for the content analyses include: What theoretical frameworks underpin these concepts? Which assessment methods are used? What are the interventional components and didactical-methodological principles applied, and how are they interconnected?

## Methods

This scoping review followed the methods of Colquhoun et al. [18] and was reported according to the PRISMA-ScR guidelines [19]. The protocol was registered on December 23, 2022, in the Open Science Framework (OSF) (doi: 10.17605/OSF.IO/AXZSJ).

### Information sources

We searched PubMed, Scopus, Web of Science, and PsycINFO from inception to March 31, 2024 (search strategy in Table S3, additional file 1). Reference lists of relevant reports were also screened.

### Identifying relevant studies

The search results were managed in Mendeley for duplicate removal. Titles and abstracts were screened by two independent reviewers (LM, AJ). Disagreements were resolved by discussion or consultation with a third reviewer (WG). Screening was facilitated using the Rayyan web app [20].

### Eligibility criteria

Records describing PAP concepts applicable in movement-based therapies for individuals with non-communicable diseases were included. Both intervention studies and theoretical works were considered. Congress abstracts, commentaries, and reviews were excluded unless they introduced a new PAP concept. If a report lacked sufficient detail but referenced an eligible concept, only the cited publication was included. Inclusion and exclusion criteria are described in Additional file 1 (Table S2). During data extraction, some intervention studies lacked sufficient detail and did not reference additional sources. These were assessed using the TIDIER checklist [21]. If relevant information could not be extracted, the report was excluded. Additionally, interventional studies and theoretical works where physical activity was secondary to pain management or disease-specific treatment were excluded. Personal contact on site offers unique opportunities for designing interventions (such as leveraging group dynamics during physical activities) that are not possible in online-only formats. For this reason, remote/e-health-only interventions were not included, but hybrid approaches combining face-to-face and remote sessions were.

### Data extraction

One reviewer (LM) extracted data, which was verified by a second reviewer (AJ). A pilot-tested extraction sheet was used to collect: (a) Author, year, and country of publication; (b) name of the PAP concept (if no name was provided, the first author´s name was used instead (e.g. Dean et al.´s paper)); (c) target population (target patient population and target healthcare provider-group); (d) setting for which the PAP concept was developed; (e) short description of the PAP concept; (f) theoretical underpinning of the PAP concept; (g) objective(s) of the PAP concept; (h) educational (i.e. training for the intervention providers) and organizational requirements; and (i) factors relevant to the cybernetic model.

### Content analysis

We applied a combined inductive-deductive approach. Therapeutic processes were mapped using the cybernetic model, categorizing assessment, therapy goals, content/methodology, and didactic-methodological principles (DMPs) (see [22]). We then categorized data using: (a) The taxonomy of behavior change techniques (BCTs) and the behavior change wheel [23] (b) a clinical reasoning model for client behavior change [16]. Additional categories emerged inductively via qualitative content analysis [24]. Two reviewers (LM, FL) conducted the content analysis in MAXQDA, and two reviewers (AJ, WG) checked accuracy. Disagreements were resolved through discussion between LM, AJ and WG. A codebook was developed, pilot tested, and refined (Additional File 5).

### Network analysis

We performed a network analysis to examine relationships between BCTs and DMPs. Nodes represented individual BCTs and DMPs, while connections illustrated their interrelations. Analysis was conducted in Gephi (v.0.10.1) using the Fruchterman-Reingold algorithm [25] with optimized settings (“Area” set to 100, “Gravity” to 10, and “Speed” to 1) for balanced node distribution [26].

## Results

### Study selection

**Fig. 1:**
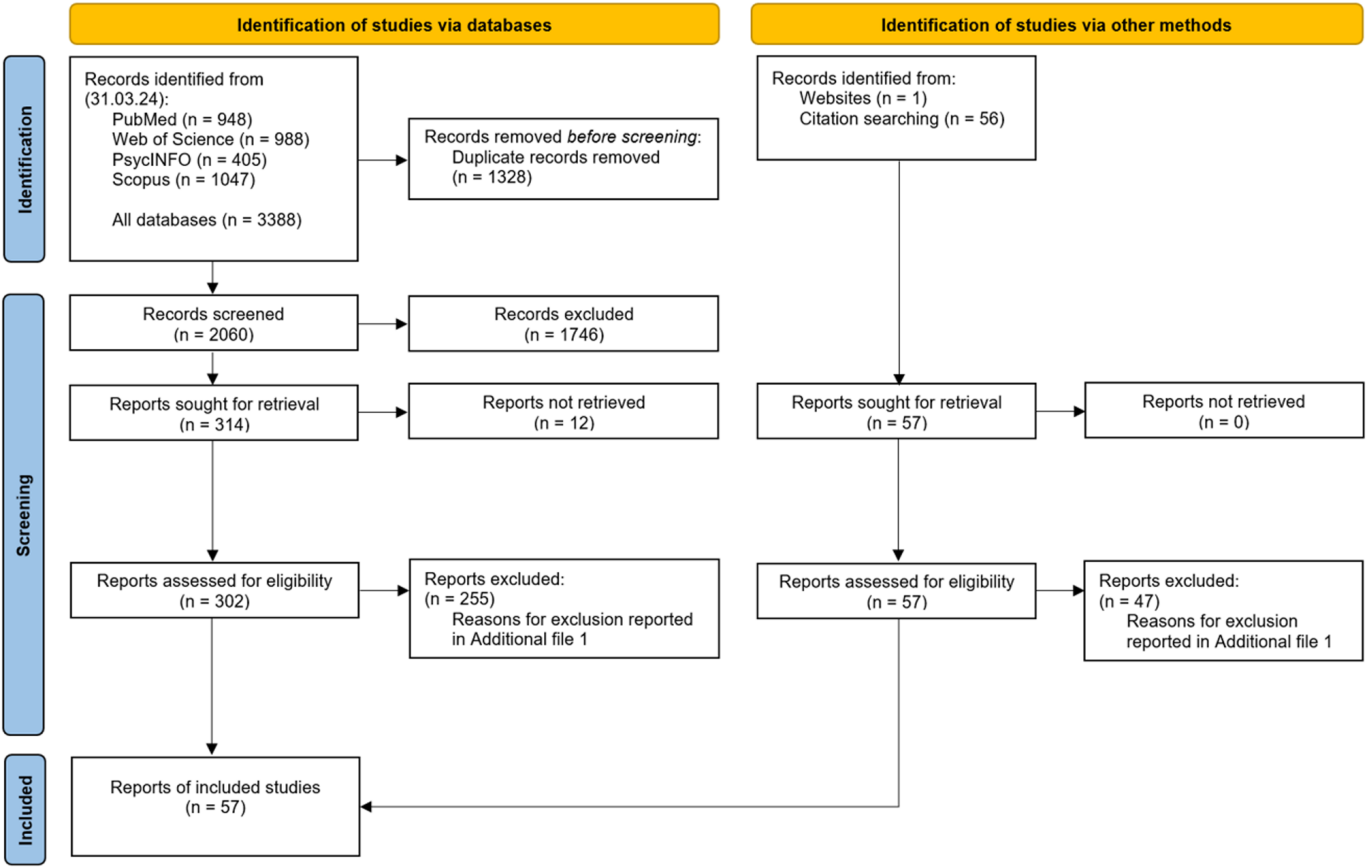
Study selection process

Figure 1 illustrates the study selection process. The systematic search yielded a total of 3388 records; 2060 remained after removing duplicates. Title and abstract screening excluded 1746 records. Of the remaining 314 full texts, 12 were inaccessible. After reviewing 302 reports, 47 were included. Screening reference lists added 57 full texts; 10 met inclusion criteria, bringing the total to 57 reports [11, 12, 14–16, 27–78]. Exclusion reasons (*n* = 302) are in Table S4 (Additional file 1), with the most common being the absence of a PAP concept (*n* = 136).

### Study characteristics

Table 1 lists all 57 included PAP concepts as well as their name and aim, the target group and the setting as well as the theoretical underpinning. (Additional file 2 contains an extended version of this table, in which, for example, a brief description of the concepts can also be found.)

**Table 1:** Overview of included interventional studies and theoretical works

Author, year, country	Name of the PAP concept	Objective of the concept	Target patient population	Target healthcare provider group	Setting	Theoretical underpinning of the concept
Adams et al., 2015 [27] Canada	Group medical visits (GMV)	Increase PA in patients with chronic depression.	Patients with depression	Psychiatrist & Exercise therapist	Primary care setting	Did not report any theoretical foundations or reference commonly recognized models and theories in the field
Alanko et al., 2022 [28] Finland	Advisory treatment protocol of SEMC.	Implementation of regular PA into daily life and give guidance on other health-promoting habits such as diet, rest and the reduction of substance use.	Patients with non-communicable diseases	Physican, Physiotherapists, Nutritional therapists, Psychologist	Sports and Exercise Medicine Clinic (SEMC)	Did not report any theoretical foundations or reference commonly recognized models and theories in the field
Borg et al., 2017 [29] Sweden	Behavioral medicine intervention	Evaluate the effects of a behavioral medicine intervention in exercise-based cardiac rehabilitation on exercise adherence, muscle endurance, level of PA, physiological parameters, anxiety and depression, health-related quality of life, patient enablement and self-efficacy, compared with usual care.	Patients with coronary artery disease (CAD)	Physiotherapists	Physiotherapy	Control Theory (CT) as part of Social cognitive theory (SCT)
Brauer et al., 2024 [30] Australia	Brauer et al.‘s paper	Determine if a group program of exercise aimed to increase physical fitness and gait performance incorporated with a self-management approach results in a greater and more sustained improvement in free-living PA (steps/day) and secondary outcomes than usual care.	People with Parkinson’s disease (PD)	Physiotherapists	University gait laboratory and the gym of hospital or community-based physiotherapy outpatient services	Health Action Process Approach (HAPA)
Bricca et al., 2022 [31] Denmark	MOBILIZE intervention	Improve health-related quality of life.	People with multimorbidity	Physiotherapists	Clinical practice	Medical Research Council Framework
Casey et al., 2018 [32] Ireland	Exercise combined with Acceptance and Commitment Therapy (ExACT)	Promote psychological flexibility through methods that encourage openness, awareness and engagement. The overall aim is to reduce pain-avoidant behaviors, in the service of living a rich and meaningful life.	Patients with a chronic pain condition	Physiotherapists	Physiotherapy department of the hospital (Pain clinic)	Acceptance and Commitment Therapy (ACT)
Cheng et al., 2022 [33] Australia	Cheng et al.‘s paper	Reduce sedentary behavior via three target behaviors: (i) replace sedentary behavior with stepping; (ii) replace sedentary behavior with standing; (iii) break up prolonged bouts of sedentary behavior.	People with chronic obstructive pulmonary disease (COPD)	Physiotherapists	Pulmonary rehabilitation	COM-B Model; Behavior Change Wheel (BCW)
Christiansen et al., 2018 [34] USA	Christiansen et al.‘s paper	Increase PA after total knee replacement.	Patients after total knee replacement	Physiotherapists	Outpatient physical therapy clinic	Social cognitive theory (SCT)
Christie et al., 2021 [35] USA	Integrative Medicine Fitness Program (IM-FIT)	Increasing PA and strength training, improving nutrition, and facilitating stress management and behavior change.	Cancer survivors	Physiotherapists, Dietitian, Psychologist	Integrative Medicine Center outpatient clinic	Did not report any theoretical foundations or reference commonly recognized models and theories in the field
Cramp et al., 2020 [36] UK	Promoting engagement in PA in early rheumatoid arthritis (PEPA-RA)	Support long-term PA engagement to optimise maintenance of physical function.	Adults with rheumatoid arthritis	Physiotherapists	Community hospitals	Determination Theory (SDT); (COM-B) Model
Daley et al., 2004 [37] UK	Sheffield women’s exercise and well-being project	Investigate the effectiveness of exercise therapy in changing exercise behavior and attitudes in breast cancer survivors by including 3- and 6-month follow-up assessment of outcomes measures.	Female breast cancer survivors	Exercise specialist	Exercise therapy	Transtheoretical Model (TTM)
de Vries et al., 2013 [15] Netherlands	Coach2Move	Promoting PA in the broad population of older adults suffering from or at risk of mobility problems.	(Pre-)frail older adults with mobility problems and/or a physically inactive lifestyle	Physiotherapists	Physiotherapy practices	Own clinical reasoning model (consistent with ICF)
Elsman et al., 2014 [38] Netherlands	Maintenance program for the SLIMMER diabetes prevention intervention	Sustain a healthy diet and PA pattern by targeting knowledge, attitudes, subjective norms and perceived behavioral control of the participants.	Diabetes prevention intervention for high-risk individuals	Physiotherapists, Dietician	Primary health care	Transtheoretical Model (TTM); Theory of Planned Behaviour (TPB); Self-determination Theory (SDT)
Elven et al., 2015 [16] Sweden	Clinical Reasoning model focused on clients’ Behavior Change with reference to Physiotherapists (CRBC-PT)	Develop and validate a conceptual model to guide physiotherapists’ clinical reasoning focused on clients’ behavior change.	Any patient	Physiotherapists	Physiotherapy practice	Own clinical reasoning model (consistent with ICF)
Focht et al., 2004 [39] USA	Group-mediated cognitive behavioral PA intervention program (GMCB)	Encourage patients to learn lifestyle adaptations and to adhere to the skills that encourage self-regulation of health behavior.	Participants who were eligible for inclusion in cardiac rehabilitation	Certified exercise leaders	Cardiac rehabilitation	Social Cognitive Theory (SCT)
Foster et al., 2012 [40] UK	Let’s Get Moving – A PA care pathway.	Increase the PA levels of adults (16+) not meeting the Chief Medical Officer’s (CMO’s) recommendations for PA.	Any patient between 16 and 74 years of age who is classified as being less than physically active	Primary care practitioners	Primary care	Did not report any theoretical foundations or reference commonly recognized models and theories in the field
Geidl et al., 2014 [12] Germany	Behavioral exercise therapy (BET)	Basic treatment goals of behavioral exercise therapy are – in addition to improved physical functioning – the promotion of a physically active lifestyle. Behavioral exercise therapy, therefore, especially addresses modifiable personal factors that determine the level of PA.	Individuals with chronic diseases	Physiotherapists	Rehabilitation	The integrative model of motivation and volition for the initiation of regular health-enhancing PA; Health Action Process Approach (HAPA); Motivation–Volition (MoVo)-Processmodel
Groen et al., 2021 [41] Netherlands	“Veerkracht” (“resilience”)	Development of a patient-centered and goal-directed exercise program entitled “Veerkracht” (“resilience”) to improve physical functioning in relation to daily activities, regular PA, and/or intentional exercise.	Patients with metastatic breast cancer	Physiotherapists	Physical therapy	Did not report any theoretical foundations or reference commonly recognized models and theories in the field
Hassett et al., 2022 [42] Australia	Brief PA counselling by physiotherapists (BEHAVIOUR)	Delivering a multi-faceted implementation strategy to teams of hospital-based physiotherapists to support them to implement brief PA counselling into routine care.	Patient participants are likely to be affected by common and/or burdensome conditions such as, but not limited to, osteoarthritis, lower limb amputations, chronic pain, stroke, brain injury, cardiac and pulmonary disease.	Physiotherapists	South Western Sydney Local Health District	COM-B Model; Self-Determination Theory (SDT)
Helmink et al., 2010 [43] Netherlands	BeweegKuur programme	Achieve health benefits through increased PA and improved dietary behavior for patients with type 2 diabetes or prediabetes. The primary objective is to promote a healthy lifestyle in terms of PA and dietary behavior.	Patients with diabetes	Practice nurse, Physiotherapists	Primary health care	Did not report any theoretical foundations or reference commonly recognized models and theories in the field
Hilberdink et al., 2020 [44] Netherlands	Hilberdink et al.‘s paper	Identify axSpA-specific exercise determinants and connect these with effective intervention components to optimize exercise behavior in people with axSpA.	Individuals with axial spondyloarthritis (axSpA)	Physiotherapists, Exercise therapists	Outpatient rehabilitation center	I-Change Model
Johannesen et al., 2024 [45] Denmark	Johannesen et al.‘s paper	Assess the concepts’ usability in constructing and implementing different interventions (focusing on individualized energy distribution and meaningful engagement in PA).	Patients with sleep apnea	Physiotherapists, Occupational therapists	Zealand University hospital at the Department of Occupational and Physiotherapy	Sense of Coherence (SOC) theory
Johnson et al., 2020 [46] UK	Individualised PA and physiotherapy behavior change intervention tool	Assess whether it is feasible to deliver an individually tailored physiotherapy and behavior change based PA intervention for breast cancer survivors.	Breast cancer survivors	Physiotherapists	A secondary care breast cancer unit	COM-B Model; Behavior Change Wheel (BCW)
Jones et al., 2016 [47] Australia	Novel and complex physical therapist intervention (myMoves)	Increasing PA after acquired brain injury.	Individuals with an acquired brain injury	Physiotherapists	Physical therapy	PRECEDE-PROCEED model
Knittle et al., 2015 [48] Netherlands	Knittle et al.‘s paper	Examining the effects of an intervention which combines motivational interviewing and self-regulation coaching to specifically target autonomous motivation, self-efficacy and PA among sedentary patients with RA by combining motivation and action phase-related components.	Patients with rheumatoid arthritis (RA)	Physiotherapists, Rheumatology nurse	Leiden University Medical Center	Did not report any theoretical foundations or reference commonly recognized models and theories in the field.
Labie et al., 2024 [49] Belgium	Best-practice KOA care (BPC)	Investigating the effectiveness of cognitive behavioral therapy for insomnia (CBTi) combined with best-practice KOA care (BPC) compared to best-practice KOA care and lifestyle education.	Patients with knee osteoarthritis (KOA)	Physiotherapists	Physical therapy settings in the areas of Brussels and Leuven	Did not report any theoretical foundations or reference commonly recognized models and theories in the field
Laitakari et al., 1998 [50] Finland	Practical model of counseling on health-related PA	Aid practical decision making in the counseling of health-related PA.	-	Health care professionals	Preventive, curative and rehabilitative work	PRECEDE-PROCEED model
Larkin et al., 2017 [51] Ireland	Communitybased intervention to promote PA in rheumatoid arthritis (CIPPARA)	Promote PA in RA (CIPPA-RA) is to determine if a theory-based intervention delivered in a community setting will promote PA behavior in people who are newly diagnosed with RA compared to a control group.	People who have RA	Physiotherapists	Community setting	Theory of Planned Behavior (TPB); Behavior Change Wheel (BCW)
Larkin et al., 2024 [52] Ireland	Promoting PA in rheumatoid arthritis through a physiotherapist led behavior changebased intervention (PIPPRA)	The objective of this study was to examine the feasibility of a physiotherapist led, behavior change (BC) theory-informed, intervention to promote PA in people with RA who have low levels of current PA.	People With Rheumatoid Arthritis	Physiotherapists	University Hospital Limerick (UHL) in the outpatient rheumatology department and the hospital’s Clinical Education and Research Centre (CERC)	Change Wheel (BCW); Medical Research Council framework for complex interventions
Lein et al., 2017 [14] UK	Health-Focused Physical Therapy Model (HFPTM)	Develop and validate a Health-Focused Physical Therapy Model (HFPTM) that could be integrated into and compatible with the Guide to Physical Therapist Practice’s Patient/Client Management Model.	Patients & Clients	Physiotherapists	Physical Therapist Practice	Theory of Reasoned Action (TRA); Social Cognitive Theory (SCT); Transtheoretical Model (TTM)
Louie et al., 2022 [53] Australia	Maximising Abilities, Negotiating and Generating Exercise options (MANAGE)	Examine the feasibility of a 12-week multiple sclerosis self-management program (MANAGE), involving group-based exercise, education and community integration. Secondarily, the impact of the program on walking, balance, fatigue, quality of life and exercise barriers and benefits was explored.	People with multiple sclerosis	Physiotherapists, Exercise physiologist	Outpatient hospital setting	Did not report any theoretical foundations or reference commonly recognized models and theories in the field
Ma et al., 2019 [54] Canada	ProACTIVE SCI	Significant improvements in PA behavior, aerobic fitness and psychosocial predictors of PA.	People with spinal cord injury (SCI)	Physiotherapists	Health and fitness Professional settings (e.g., physiotherapy clinics, personal training gyms)	Health Action Process Approach (HAPA)
Manning et al., 2014 [55] UK	Education, Self-Management, and Upper Extremity Exercise Training in People with Rheumatoid Arthritis [EXTRA] program	To evaluate the effectiveness of a brief supervised education, self-management, and global upper extremity exercise training program, supplementing a home exercise regimen, for people with rheumatoid arthritis.	People with rheumatoid arthritis	Physiotherapists	Rheumatology clinics and physiotherapy departments of 4 public hospitals	Social Cognitive theory (SCT)
Moore et al., 2018 [56] USA	A Framework and Resources for Shared Decision Making	Synthesize general and rehab-specific SDM information; present a model of SDM stages, outcomes, and facilitators relevant to physical therapy; relate how TDF domains help to identify local facilitators and barriers to SDM implementation; and provide resources that physical therapists can access to increase their SDM capabilities.	Patients	Physiotherapists	Physical Therapy	Theoretical Domains Framework (TDF)
Moore et al., 2020 [57] UK	PARAS	Equip the stroke survivors with the knowledge and skills to enable them to self-manage their PA and sedentary behavior in the long-term.	Stroke survivors	Physiotherapists, Occupational therapists	Community stroke services	Social Cognitive Theory (SCT); Health Belief Model; Self-Regulation Theory
Morris et al., 2022 [58] UK	We Walk	Increase PA by promoting outdoor walking with support from a walking buddy.	People with stroke (PWS)	Health and exercise professionals	Community rehabilitation stroke services	Behaviour Change Wheel (BCW); Theoretical Domains Framework (TDF); Health Action Process Approach (HAPA).
Motl et al., 2022 [59] USA	Health-Focused Physical Therapy Model (HFPTM)	Provide a systematic approach based on an experimental medicine perspective for a 4-step process in the design, delivery, and evaluation of behavior change interventions that can be applied to health promotion in physical therapy research and practice.	Non-communicable diseases in society	Physiotherapists	Physical Therapist Research and Practice	Theoretical Domains Framework (TDF); Behaviour Change Wheel (BCW); COM-B Model.
O’Dwyer et al., 2017 [60] Ireland	Increasing PA in Ankylosing Spondylitis (INPACT-AS)	Motivate and support individuals to participate in PA, taking into account their needs, ambitions, preferences and available resources.	Adults with ankylosing spondylitis (AS)	Physiotherapists	Rheumatology outpatient clinics	Did not report any theoretical foundations or reference commonly recognized models and theories in the field
Pollock et al., 2001 [61] USA	Pollock et al.‘s paper	Integrate exercise promotion into psychotherapy treatment is presented.	Depressed patients	Psychotherapists	Psychotherapy treatment	ECBIS (dimensions: Emotional, Cognitive, Behavioral, Interpersonal, Social systemic)
Quinn et al., 2016 [62] UK	ENGAGE-HD	Develop self-determined PA behaviors through intentionally promoting feelings of autonomy, competence, and relatedness.	People with Huntington disease	Physiotherapists, Occupational therapists, Research nurses, Exercise trainers	Physical therapy	Self Determination Theory (SDT)
Reid et al., 2012 [63] Canada	Motivational counselling (MC) intervention	Comparing long-term PA between a theoretically guided motivational counselling intervention group and a usual care control group. The behavioral objective was to have the patient engage in 330 min of PA at a moderate or vigorous intensity 5 or more days per week.	Patients with coronary artery disease	Physiotherapists	Cardiac rehabilitation	Ecological perspective (individual, social-environmental and physical-environmental factors)
Rethorn et al., 2022 [11] USA	From expert to coach	(1) Describe a health coaching approach to facilitating behavior change; (2) Discuss behavior change theories and models underpinning health coaching; and [3) Provide suggestions for how PTs can integrate health coaching in clinical practice.		Physiotherapists	Physiotherapy	Transtheoretical model (TTM); Self Determination Theory (SDT)
Richmond et al., 2018 [64] UK	PROSPER (PRevention Of Shoulder ProblEms Trial)	Improve shoulder function through an early, progressive, home-based exercise programme with integrated behavioral support strategies.	Musculoskeletal shoulder problems are common after breast cancer treatment	Physiotherapists	UK clinical centres	Did not report any theoretical foundations or reference commonly recognized models and theories in the field
Ryan et al., 2017 [65] UK	iStep-MS	Investigate the feasibility of delivering a behavior change intervention to increase PA and reduce sedentary behavior as part of routine physiotherapy care.	People with MS	Physiotherapists	Berkshire MS Therapy Centre	Did not report any theoretical foundations or reference commonly recognized models and theories in the field
Salmon et al., 2019 [66] UK	Salmon et al.‘s paper	Enable RA patients to develop their capability to self-manage fatigue through modifying PA, identify physical and social opportunities to support PA modification, and enhance motivation to modify PA within the context of their fatigue.	Adults with RA who experience fatigue	Physiotherapists	Community and hospital settings	Behaviour Change Wheel (BCW); Theoretical Domains Framework (TDF); COM-B Model; Self Determination Theory (SDT)
Saxton et al., 2013 [67] UK	Pragmatic exercise intervention for people with multiple sclerosis (ExIMS Trial)	Investigate the effects of the pragmatic exercise intervention on PA behavior and important health outcomes up to 9 months of follow-up, as well as cost-effectiveness of the intervention in relation to standard care.	People with multiple sclerosis (MS)	Physiotherapists, Exercise physiologist	Hospital	Transtheoretical model (TTM)
Stevens et al., 2018 [68] Netherlands	Patient-specific method for physiotherapy goal setting (PSG): a user-centered design	With the PSG, patients can be actively involved in identifying, prioritizing and rating their performance of the most important activities they are having problems with due to their disorder.	Patients with chronic disorders	Physiotherapists	Physiotherapy	Did not report any theoretical foundations or reference commonly recognized models and theories in the field
Sudeck et al., 2011 [69] Germany	Volitional Interventions within Cardiac Exercise Therapy (VIN-CET)	Promote PA and improve health-related quality of life in cardiac rehabilitation.	Individuals with cardiovascular diseases	Exercise therapists	Cardiac rehabilitation	Rubicon theory of action phase; Social Cognitive Theory (SCT)
Tatta et al., 2022 [70] USA	Mindfulness and acceptance–based interventions (MABIs) Acceptance and Commitment Therapy (ACT)	Allowing all experiences, even the distressing ones, as part of the human experience. This teaches the patient to tolerate discomfort and uncertainty as simply another transient mental state.	People with mental illness and stress-related chronic illnesses	Physiotherapists	Physical therapist practice	Theory of mindfulness
Teuwen et al., 2024 [71] Netherlands	The Longstanding-EXercise Therapy in people with RA (L-EXTRA)	This study is the first evaluation of a personalised, longstanding, supervised exercise therapy programme in patients with RA with severe disability due to persisting disease activity, joint damage and/or comorbidities.	People With Rheumatoid Arthritis and severe functional limitations	Physiotherapists	Primary care physical therapy practice in the neighbourhood/place of residence of the patient or at the patient’s home.	International Classification of Functioning, Disability and Health (ICF); Hypothesis Oriented Algorithm for Clinicians.
van der Ploeg et al., 2006 [72] Netherlands	Sport stimulation program ‘Rehabilitation & Sports’ (R&S) combined with the daily PA promotion program ‘Active after Rehabilitation’ (AaR)	Improving sport participation after rehabilitation.	Patients with amputation, stroke, neurological disorders, orthopaedic disorders, spinal cord injury, rheumatic related disorders, back disorders or whiplash	Sport counsellor	Rehabilitation centres	Transtheoretical Model (TTM)
van Grootel et al., 2024 [73] Netherlands	Goal-directed movement intervention (GOAL)	To describe the development of a goal-directed movement intervention in two medical wards, including recommendations for implementation and evaluation.	Hospitalized patients	Health care professionals	Pulmonology and nephrology/gastroenterology wards of the University Medical Centre Utrecht	Intervention mapping approach
van Nimwegen et al., 2010 [74] Netherlands	ParkFit	Stimulate patients to increase their PA levels.	Patients with Parkinson’s disease (PD)	Physiotherapists	Physical therapy	Social Cognitive Theory (SCT); Transtheoretical Model (TTM)
Willett et al., 2021 [75] UK	Willett et al.‘s (2021) paper	Optimise PA adherence in patients with lower-limb OA.	Patients with lower-limb osteoarthritis	Physiotherapists	Royal Orthopaedic Hospital (ROH)	Theoretical Domains Framework (TDF)
Willett et al., 2023 [76] UK	Willett et al.‘s (2023) concept	Create a more motivationally empowering treatment climate to deliver BCTs that target the determinants of PA adoption and maintenance in patients with lower-limb OA.	Patients with lower-limb osteoarthritis	Physiotherapists	National Health Service physiotherapy outpatient setting	Self Determination Theory (SDT)
Wittink et al., 2024 [77] UK	Wittink et al.‘s concept	The goal of this Active Living after Stroke (ACTS) study was to develop a behavior change intervention to improve walking performance in stroke survivors as a form of task-specific aerobic training.	Stroke survivors	Physiotherapists, Exercise therapists	Primary care settings	Participatory Action Research (PAR) design approach
Wolf et al., 2021 [78] Germany	ImPuls	Combining the current evidence on the efficacy of MVAE and sustained exercise behavior change with specific demands of a real-world outpatient health care setting, ImPuls was developed as a manualized group exercise intervention.	Patients with major depressive disorders, insomnia, panic disorder, agoraphobia and PTSD	Exercise therapists	Outpatient mental health care system	Did not report any theoretical foundations or reference commonly recognized models and theories in the field

Abbreviations: PA: physical activity

Most PAP concepts (*n* = 44, 77.2%) were published as part of an interventional study. The remaining concepts constituted theoretical work. PAP concepts were scattered across three continents, with Europe being the most common location (*n* = 41, 71.9%). Most PAP concepts had been published between 2011 and 2024 (*n* = 50, 87.7%). Table 2 summarizes the concept characteristics.

**Table 2:** Summary of characteristics of included interventional studies and theoretical works

Characteristic	*N* (%)
Location	
UK	15 (26.3)
The Netherlands	12 (21.1)
USA	8 (14.0)
Australia	5 (8.8)
Ireland	4 (7.0)
Canada	3 (5.3)
Germany	3 (5.3)
Finland	2 (3.5)
Sweden	2 (3.5)
Denmark	2 (3.5)
Belgium	1 (1.8)
Year of publication	
1990 to 2000	1 (1.8)
2001 to 2010	6 (10.5)
2011 to 2024	50 (87.7)
Overarching characteristic	
Intervention study	44 (77.2)
Theoretical work	13 (22.8)
Duration	
Short (up to 8 weeks)	10 (17.5)
Medium (between 9 and 24 weeks)	20 (35.1)
Long (25 weeks and longer)	6 (10.5)
Not specified	21 (36.8)
Number of sessions	
Low (up to 8)	17 (29.8)
Medium (between 9 and 24)	11 (19.3)
High (25 and more)	8 (14.0)
Not specified	20 (35.1)
Format of delivery	
Group-based	7 (12.3)
One-to-one	22 (38.6)
Combined	12 (21.1)
Not specified	16 (28.1)
Mode of delivery	
Face-to-face	19 (33.3)
Combined (face-to-face and phone	19 (33.3)
calls or online tools)	
Not specified	19 (33.3)
Delivered by	
Movement-based therapists only	31 (52.6)
Movement-based therapists together	6 (17.5)
with other health care professionals	
(e.g. psychologist, dietician)	
Not specified	20 (29.8)
Target patient population	
Orthopaedical & Rheumatological	13 (22.8)
Neurological	12 (21.1)
Oncological	5 (8.8)
Psychiatric	4 (7.0)
Cardiological	4 (7.0)
Endocrinological	2 (3.5)
Transdiagnostic	7 (12.3)
Others	10 (17.5)

### Theoretical underpinning of PAP concepts

PAP concepts utilize various theoretical approaches. Behavioral theories guided concept development to explain the behavioral changes and inform PAP content and methods. The concepts incorporated one or more of the following behavior change theories or models: (a) behavior change wheel or COM-B model (*n* = 14), (b) social cognitive theory (*n* = 9), (c) self-determination theory (*n* = 8), (d) transtheoretical model (*n* = 7), (e) health action process approach (*n* = 4), (f) theory of planned behavior (*n* = 3), (g) mindfulness-based theory (*n* = 2), (h) rubicon theory of action phases (*n* = 1), (i) motivation-volition process model (*n* = 1), (j) integrative model of motivation and volition for the initiation of regular health-enhancing physical activity (*n* = 1), k) goal-setting theory (*n* = 1), l) theory of reasoned action (*n* = 1), m) health belief model (*n* = 1), o) I-change model (*n* = 1), p) sense of coherence model (*n* = 1), q) ecological perspective (*n* = 1), and r) the ECBIS psychotherapy model (*n* = 1). Additionally, some interventional studies apply implementation frameworks for planning and implementation, either with behavior change theories or independently, including (a) theoretical domains framework (*n* = 5), (b) PRECEDE-PROCEED model (*n* = 2), (c) medical research council framework (*n* = 1), (d) intervention mapping approach (*n* = 1), and (e) participatory action research ​design approach (*n* = 1). Fourteen papers did not report theoretical foundations.

### Core therapeutic processes

Four core therapeutic processes were identified: (1) assessment; (2) therapy goals; (3) content and methodology; and (4) didactic-methodological principles. The extraction results for each are detailed below.

### Assessment

Table 3 summarizes the assessment results. Most concepts (*n* = 50, 88%) included assessment in their PAP strategy. With respect to assessment domains, 47 concepts (82%) specified which domains were evaluated. The most reported was physical activity behavior (*n* = 34), while previous physical activity experiences were least frequently assessed (*n* = 4).

**Table 3:** Summary of assessment categories and related measuring instruments

(Sub-)Category and definition	Number of PAP concepts reporting	Measuring instruments (if reported)
**Physical Activity Behavior (Outcome)** This category includes all measurements related to current movement behavior, encompassing the frequency, intensity, duration, and types of physical activities performed by an individual (e.g. questionnaire or accelerometer/pedometer). This also includes all forms of self-monitoring related to physical activity.	n = 34	Device-based measurement ActiGraph GT1M [67]; ActiGraph GT3X [29, 34]; ActiGraph GT9X [54]; ActivPAL3 micro [33]; Fitbit Zip Pedometer [27, 32]; FitBit Charge 5 [45]; Garmin Vivosmart fitness tracker [30]; Physical Activity Monitor (PAM AM 400) [73]; Onmood Pedometer [71]; Yamax Pedometer [78]; Yamax SW-200 Digi-walker Pedometer [65]; Digi-walkers (New Lifestyles, Inc.) [39]; TracmorD Triaxial Accelerometer [74] Questionnaires General Practitioner Physical Activity Questionnaire (GPPAQ) [40]; Godin-Shepard Leisure-Time Exercise Questionnaire (GLTEQ) [46]; LASA Physical Activity Questionnaire (LAPAQ) [15]; Leisure Time PA Questionnaire (LTPAQ) [54]; Sedentary Behaviour Questionnaire [33]; Short Questionnaire to Assess Health-Enhancing Physical Activity [48] Others Diary [29]; Interview (based on COM-B) [42]
**Previous physical activity experiences** This category includes all measurements related to the sport or exercise history.	n = 4	Informal survey through conversation [11, 62, 63, 72]
**Other health-related behaviors** This category includes all measurements related to other health-related behaviors (e.g. dietary behavior or smoking status).	n = 6	Lifestyle Questionnaire [28]; Informal survey through conversation [50, 63]
**Physical capabilities & skills** This category includes all measurements related to physical capabilities and skills to engage in the activity-related behavior (e.g. strength, mobility or balance).	n = 13	Six-minute walk test [15, 28, 34, 41, 45, 53]; Stress ECG-test [28]; Sit-ups form a chair [28]; Timed Up & Go Test (TUG) [15, 41]; 5 times sit to stand (5TSTST) [41]; Squats [28]; Hand-grip Strength [15, 28]; One repetition maximum—lower extremities (1RM-LE) [41]; One repetition maximum—upper extremities (1RM-UE) [41]; One-leg stance [28]; Berg Balance Scale (BBS) [15]; Short physical performance battery (SPPB) [41]; Range Of Motion (ROM) [15, 64]; Functional Reach [53]; Rivermead Mobility Index [57]; Interview (based on COM-B) [33]; Activity of daily living (ADL) [45]
**Psychological capabilities & skills** This category includes all measurements related to psychological capability and skills to engage in the activity-related behavior (e.g. cognitions, beliefs, expectations, fear, self-efficacy etc.).	n = 18	Barrier and Task Self Efficacy Scale [46]; Exercise Benefits Barriers Scale [53]; Confidence scale [64]; Fear of Movement Scale for Osteoarthritis questionnaire (FMSO) [34]; Chronic Pain Acceptance Questionnaire (CPAQ) [70]; Interview (based on COM-B) [33]
**Additional psychological factors** This category includes all measurements related to additional psychological factors that are not directly related to the activity-related behavior (e.g. depression, well-being, mindfulness etc.)	n = 5	Warwick-Edinburgh Mental Well-Being Scale [57]; Generalized Anxiety Disorder Questionnaire (GAD-7) [35]; Distress Thermometer [35]; Patient Health Questionnaire (PHQ-9) [35]; Five Facets of Mindfulness [70]; State Mindfulness Scale for Physical Activity [70]; Valued living questionnaire (VLQ) [70]
**Biomedical influencing factors** This category includes all measurements related to biomedical deficits, such as disease and tissue pathology, as well as health status indicators such as BMI and overall health metrics.	n = 19	Numerical pain rating scale (NPRS) [41]; Multidimensional fatigue inventory (MFI) [41]; Visual analog scale for fatigue (VASF) [41]; Abbreviated fatigue questionnaire (AFQ) [41]; Fatigue Severity Scale [53]; Multiple Sclerosis Impact Scale (MSIS-29) [53]; Rheumatoid Arthritis Disease Activity Index (RADAI) [48]; Electrocardiogram (ECG) [28]; Knee Outcome Scale (KOS) [34]; Disability of Arm Shoulder and Hand (DASH) [46]; Hip Disability and Osteoarthritis Out-come Score (HOOS) [56]; Western Ontario & McMaster Universities Osteoarthritis Index (WOMAC) [46]; Medical Outcomes Study (MOS) [34]; 12-item Short Form Survey (SF-12) [56]; Tanita® scale (Body weight; percent body fat) [35]
**Social and physical opportunities** This category includes both physical and social opportunities, encompassing the environmental and social factors that influence an individual's behavior.	n = 9	Interview (based on COM-B) [33]
**Others** This category includes all factors that could not be covered by other categories, e.g., quality of life.	n = 9	Quality of life Questionnaire (RAND-36) [28]; Patient-Specific Complaints (PSC) [15]; Canadian Occupational Performance Measure (COPM) [45]

### Therapy goals

Most concepts (*n* = 54, 95%) mentioned therapy goals in their PAP strategy, all incorporating goal setting (see results regarding content and methodology below). Temporal aspects of goals were specified in 20 concepts, while 10 provided content details. Regarding communication style/technique, 18 concepts provided relevant information, with 11 using motivational interviewing during goal setting. Shared decision-making was explicitly mentioned or applied in 13 concepts. Specific techniques like SMART goals were described in 9 concepts (see also Additional file 3).

### Content and methodology

**Fig. 2:**
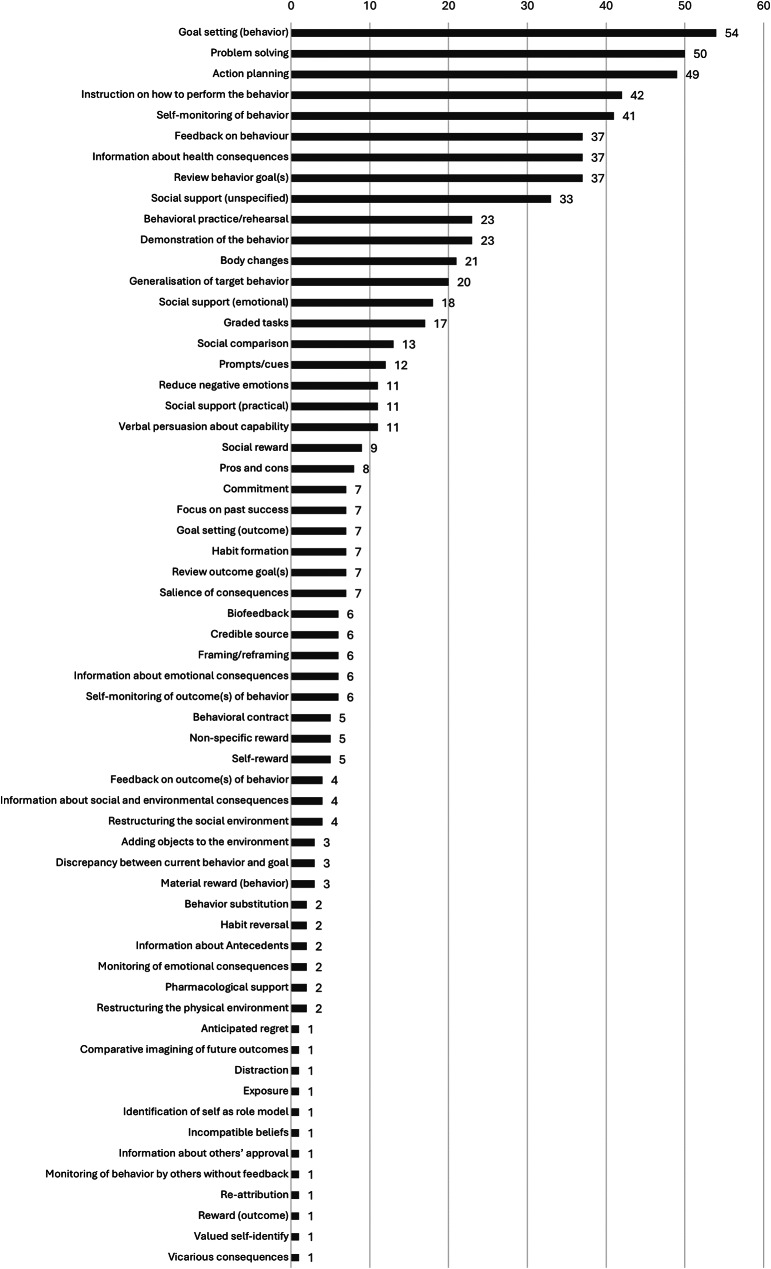
Summary of the behavior change techniques used in 57 physical activity promotion concepts

Figure 2 summarizes the extracted results, which are presented in detail in Additional File 4. The median number of BCTs per concept was 11 (range: 0 to 37), with 60 of 93 possible BCTs applied. The three most used were goal setting (*n* = 54), problem solving (*n* = 50), and action planning (*n* = 49). Additionally, 28 PAP concepts described the practical physical activity or exercise component, including endurance (*n* = 19), strength (*n* = 19), flexibility (*n* = 9), and relaxation (*n* = 3).

### Didactic-methodological principles

Most PAP concepts (*n* = 54, 95%) explicitly or implicitly described didactic-methodological principles (DMPs) (see Additional file 3). The most frequently described were “tailoring and individualization” (*n* = 47) and “active participation in shaping the therapy process by patients” (*n* = 39), followed by “consideration of interaction & language / collaborative communication” (*n* = 21) and “patients’ self-responsibility and independence” (*n* = 14). Of the 19 PAP concepts using group format (see Table 2), 11 explicitly use the group setting as a methodological-didactic principle, such as by facilitating group discussions. The least mentioned principle was “facilitating positive movement experiences, enjoyment of physical activity” (*n* = 3).

### Network analysis

**Fig. 3:**
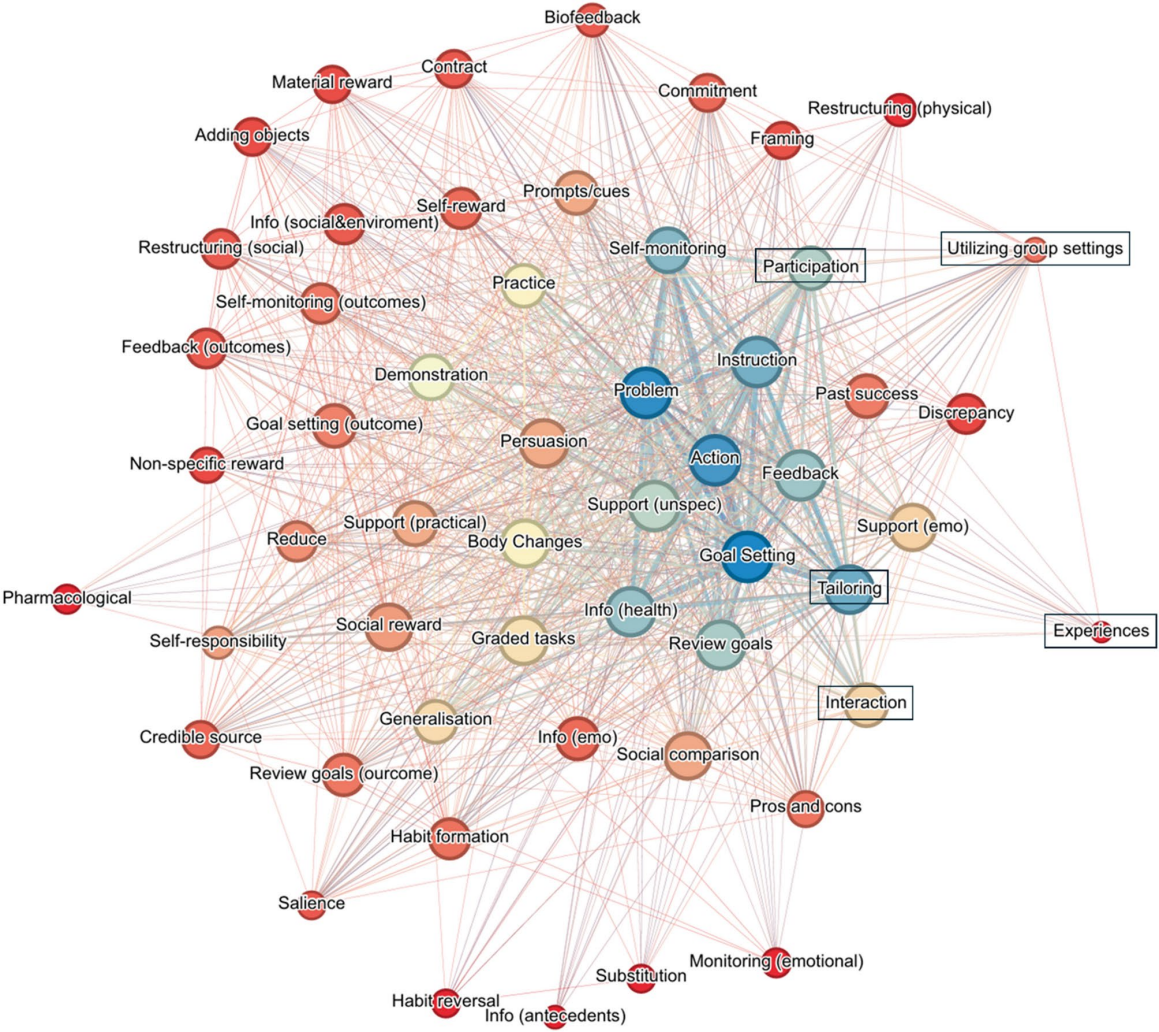
The network visualization of behavior change techniques (BCTs) and didactic-methodological principles (DMPs; highlighted with a black border) was generated via the Fruchterman-Reingold algorithm, which clusters techniques based on their connectivity. Node size reflects degree centrality, with larger nodes indicating greater interconnectedness. Node color ranges from red (low weighted degree) to blue (high weighted degree). To highlight stronger or multiple associations, single (simple) connections between techniques were excluded from the visualization. Note: For improved readability, some BCTs and DMPs have been abbreviated. The corresponding full terms are as follows: Action = Action planning; Adding objects = Adding objects to the environment; Contract = Behavioral contract; Demonstration = Demonstration of the behavior; Discrepancy = Discrepancy between current behavior and goal; Experiences = Facilitating positive movement experiences, enjoyment of physical activity; Feedback = Feedback on behavior; Feedback (outcomes) = Feedback on outcome(s) of behavior; Framing = Framing/reframing; Generalization = Generalization of target behavior; Goal setting = Goal setting (behavior); Info (antecedents) = iInformation about antecedents; Info (health) = Information about health consequences; Info (social & environment) = Information about social and environmental consequences; Instruction = Instruction on how to perform the behavior; Interaction = consideration of interaction and language/collaborative communication; Material reward = Material reward (behavior); Participation = Active participation in shaping the therapy process; Past success = Focus on past success; Pharmacological = Pharmacological support; Practice = Behavioral practice/rehearsal; Problem = Problem solving; Reduce = Reduce negative emotions; Restructuring (physical) = Restructuring the physical environment; Restructuring (social) = Restructuring the social environment; Review goals = Review behavior goal(s); Review goals (outcome) = Review outcome goal(s); Salience = Salience of consequences; Self-monitoring: Self-monitoring of behavior; Self-monitoring (outcomes) = Self-monitoring of outcome(s) of behavior; Self-responsibility = Patients’ self-responsibility and independence; Support (emo) = Social support (emotional); Support (unspec) = Social Support unspecified; Substitution = Behavior Substitution; Tailoring = Tailoring and individualization; Utilizing group settings = Utilizing group settings

The network analysis (Fig. 3) of BCTs and DMPs, visualized using the Fruchterman-Reingold algorithm, reveals clusters of techniques based on weighted degree centrality (the weighted degree in network analysis measures a node’s total connection strength by summing the weights of its links). Central techniques—“Action Planning”, “goal setting”, “feedback on behavior”, and “instruction on how to perform the behavior” (depicted in blue)—are highly interconnected and pivotal. Node size, determined by degree centrality, reflects the number of direct connections each technique has, with larger nodes indicating greater interconnectedness with other techniques. Surrounding these central nodes are moderately connected techniques, including “social support (unspecified)”, “tailoring”, and “problem solving”, forming a secondary layer of importance. Node coloring, from red (low) to blue (high) weighted degree, highlights the significance of individual techniques. Action planning and goal setting appear as large blue nodes, emphasizing their central role. In contrast, peripheral techniques like material reward (behavior) and salience of consequences have smaller red nodes, indicating fewer connections. To emphasize stronger associations, single connections were excluded, removing weakly linked techniques and highlighting those with integral roles.

## Discussion

The scoping review summarizes 57 intervention approaches designed to promote physical activity among individuals with non-communicable diseases, as implemented by movement-based therapists. It analyzes the theoretical foundations, therapeutic content, and overarching methodological-didactical principles of these approaches. Our findings highlight substantial diversity and heterogeneity in the physical activity promotion strategies used by movement-based therapists. Notably, these approaches incorporate 60 distinct Behavior Change Techniques and consistently emphasize five key didactical-methodological principles: tailoring and individualization, active participation in shaping the therapy process by patients, consideration of interaction & language / collaborative communication, patients’ self-responsibility and independence, facilitating positive movement experiences, enjoyment of physical activity and leveraging the group setting to enhance outcomes.

This review extends the work of Lowe et al. [17] by including a broader range of movement-based therapists—such as physical therapists, sport therapists, kinesiologists, and exercise therapists—and offering more detailed analysis of intervention contents, principles, and theoretical frameworks. As noted by Lein et al. [14], physical activity promotion is a relatively recent focus for physical therapists. Our analysis of publication trends underscores the growing significance of this topic, with 50 of 57 identified approaches published after 2011.

The development of physical activity promotion approaches is grounded in several behavior change theories. Older, well-established rational-cognitive theories, such as social-cognitive theory and the transtheoretical model, are most frequently referenced (*n* = 23). In contrast, only a small number of approaches (*n* = 8) incorporate humanistic frameworks, such as self-determination theory (SDT). The prevalence of rational-cognitive theories likely accounts for the frequent use of techniques like goal-setting and action planning, which have been shown to be effective in movement-based therapy [79]. However, these techniques tend to assume that individuals make decisions based solely on rational, fact-based reasoning. Current research (e.g. [80, 81]) suggests that emotional experiences during movement also play a critical role in driving changes in physical activity behavior. Despite this, few approaches address the promotion of positive, enjoyable movement experiences, even those that draw on humanistic theories. Future PAP developments should integrate models such as the affective-reflective theory of physical inactivity and exercise [82] and the model of physical activity-related health competence [83, 84], to better account for the emotional and motivational aspects of behavior change.

Although movement-based therapists often rely on evidence-based, standardized treatment pathways to optimize physical function and fitness, high-quality standards in PAP are largely absent. For example, of the 57 approaches reviewed, only 34 include an initial assessment of current physical activity behavior, despite the emphasis on improving activity levels. Some approaches [14, 16] highlight the value of a biopsychosocial approach to assessment, suggesting its critical role in promoting physical activity. However, many studies lack a comprehensive initial assessment. Currently, no minimum standards exist for physical activity behavior assessments in movement-based therapy. The development of a biopsychosocial assessment tool that is comprehensive yet practical for everyday use remains a key challenge.

For movement-based therapists in practice, these findings offer new insights and potential strategies for promoting physical activity by providing an overview of useful content. Scientists can utilize the results to refine standards and guidelines in the field of PAP. In Germany, for instance, this scoping review is being combined with data on intervention effectiveness [79] to inform the co-creation of practical recommendations for promoting physical activity in rehabilitation settings.

### Strengths and limitations

A key strength of this work is the comprehensive literature search combined with the inclusion of interventional studies and theoretical works used by various movement-based therapists. However, limitations exist, as we only included PAP concepts in English and German, and we were unable to obtain the full texts of 12 potentially relevant papers. Consequently, some PAP concepts may have been overlooked.

The use of the Behavior Change Technique (BCT) taxonomy [23] is another strength, though it has certain limitations. On one hand, this taxonomy provides excellent standardization in describing PAP concepts. On the other hand, the systematic combination and arrangement of different BCTs creates a therapeutic quality that exceeds the sum of its parts. For instance, when movement-based therapists teach new motor skills (e.g., Nordic walking, which involves walking with specially designed poles), incorporating self-awareness techniques, contrast exercises, provide feedback, and social interaction in group settings (e.g., playful group exercises on walking), the complexity and quality of the therapy cannot be fully captured by classifying individual BCTs. A successful physical activity promotion program is more than just the sum of its BCTs. The Physical Activity-Related Health Competence (PAHCO) model [83] attempts to address this “holistic” aspect, emphasizing that systematically linking physical activity practice with learning and experience is central to developing the competencies required for a physically active lifestyle [84]. However, our analysis does not systematically describe these linkages within the PAP concepts. To address this, our network analysis aims to explore the integration and connections between BCTs by considering the underlying didactical methodological principles. This analysis highlights how BCTs relate to overarching principles, such as individual tailoring or fostering enjoyment of physical activity. However, it does not provide detailed insights into the methodological and didactical arrangement of BCTs. Future development of PAP concepts should more precisely describe the methodological-didactic principles and the specific connections between BCTs.

## Conclusions

This scoping review offers a comprehensive overview of 57 physical activity promotion concepts utilized by movement-based therapists (including physiotherapists, sport therapists, and exercise physiologists) for individuals with non-communicable diseases. The findings highlight considerable heterogeneity in interventional studies and theoretical works, revealing the variety of strategies employed to influence physical activity behavior. The in-depth analysis of these concepts—covering their theoretical foundations, content, underlying didactic-methodological principles, and theoretical underpinnings—provides a solid foundation for advancing physical activity promotion within movement-based therapies. This work contributes to the development of standards and guidelines for effectively integrating physical activity promotion into therapeutic practice.

## Electronic supplementary material

Below is the link to the electronic supplementary material.

Supplementary Material 1: Additional file 1 provides details on deviations from the preregistered protocol, eligibility criteria search strategies, and a list of excluded studies with reasons for exclusion.

Supplementary Material 2: Additional file 2 provides an overview of the included studies, including their characteristics.

Supplementary Material 3: Additional file 3 provides an overview of the data extracted regarding the core therapeutic processes.

Supplementary Material 4: Additional file 4 provides an overview of the data extracted regarding the behavior change techniques.

Supplementary Material 5: Additional file 5 contains the codebook used for content analysis.

## Data Availability

All data relevant to the results of this systematic review are available in the main tables and additional files. The data collected including data extraction forms can be made available upon reasonable request.

## Abbreviations

BCTs: Behavior Change Techniques
DMPs: Didactic-Methodological Principles

## References

[1] WHO. Global action plan on physical activity 2018–2030: more active people for a Healtier world. Geneva, Switzerland: World Health Organization; 2018. p. 104.

[2] Alsop T, Lehman E, Brauer S, Forbes R, Hanson CL, Healy G, et al. What should all health professionals know about movement behaviour change? An international Delphi-based consensus statement. Br J Sports Med. 2023;57:1419–27. 10.1136/bjsports-2023-106870. 37793699 10.1136/bjsports-2023-106870

[3] College of Kinesiologists of Ontario. Essential compentencies of practice for kinesiologists in Ontario. (2014). Available from: https://coko.ca/wp-content/uploads/2024/09/Essential-Competencies-of-Practice-for-Kinesiologists-in-Ontario-March-2018.pdf

[4] Exercise and Sports Science Australia. Accredited exercise physiologist scope of practice. (2023). Available from: https://www.essa.org.au/Common/Uploaded%20files/Standards/AEP%20Scope%20of%20Practice.pdf

[5] Dean E, Al-Obaidi S, de Andrade AD, Gosselink R, Umerah G, Al-Abdelwahab S, et al. The first physical therapy summit on global health: implications and recommendations for the 21st century. Physiother Theory Pract. 2011;27:531–47. 10.3109/09593985.2010.544052. 21612551 10.3109/09593985.2010.544052

[6] Chodzko-Zajko W, Taylor EM, Reeve TG. The American kinesiology association core content for kinesiology programs: from concept to curriculum. Kinesiol Rev. 2018;7:279–85. 10.1123/kr.2018-0050.

[7] Hopkins-Rosseel D, Yardley D, Turnnidge J, Dalgarno N, Kolomitro K. Developing a National consensus of the physiotherapy entry-level business and practice management core curriculum competencies: A Delphi study. Physiother Can. 2024;76:389–99. 10.3138/ptc-2022-0054. 10.3138/ptc-2022-0054 PMC12393230 40959479

[8] Jones H, George KP, Scott A, Buckley JP, Watson PM, Oxborough DL, et al. Charter to Establish clinical exercise physiology as a recognised allied health profession in the UK: a call to action. BMJ Open Sport Exerc Med. 2021;7:e001158. 10.1136/bmjsem-2021-001158. 34631147 10.1136/bmjsem-2021-001158 PMC8458347

[9] Albert FA, Crowe MJ, Malau-Aduli AE, Malau-Aduli BS. Physical activity promotion: A systematic review of the perceptions of healthcare professionals. Int J Environ Res Public Health. 2020;17. 10.3390/ijerph17124358. 10.3390/ijerph17124358 PMC7345303 32570715

[10] Brawner CA, Churilla JR, Keteyian SJ. Prevalence of physical activity is lower among individuals with chronic disease. Med Sci Sports Exerc. 2016;48:1062–7. 10.1249/MSS.0000000000000861. 26741117 10.1249/MSS.0000000000000861

[11] Rethorn ZD, Bezner JR, Pettitt CD. From expert to coach: health coaching to support behavior change within physical therapist practice. Physiother Theory Pract. 2022;38:2352–67. 10.1080/09593985.2021.1987601. 34620046 10.1080/09593985.2021.1987601

[12] Geidl W, Semrau J, Pfeifer K. Health behaviour change theories: contributions to an ICF-based behavioural exercise therapy for individuals with chronic diseases. Disabil Rehabil. 2014;36:2091–100. 10.3109/09638288.2014.891056. 24564358 10.3109/09638288.2014.891056

[13] Wikström-Grotell C, Broberg C, Ahonen S, Eriksson K. From Ling to the academic era – An analysis of the history of ideas in PT from a nordic perspective. Eur J Physiother. 2013;15:168–80. 10.3109/21679169.2013.833985.

[14] Lein DH, Clark D, Graham C, Perez P, Morris D. A model to integrate health promotion and wellness in physical therapist practice: development and validation. Phys Ther. 2017;97. 10.1093/ptj/pzx090. 10.1093/ptj/pzx090 29186634

[15] De Vries NK, Staal JB, Teerenstra S, Adang EM, Rikkert MG, Nijhuis-van der Sanden MW. Physiotherapy to improve physical activity in community-dwelling older adults with mobility problems (Coach2Move): study protocol for a randomized controlled trial. Trials. 2013;14. 10.1186/1745-6215-14-434. 10.1186/1745-6215-14-434 PMC3878551 24345073

[16] Elvén M, Hochwälder J, Dean E, Söderlund A. A clinical reasoning model focused on clients’ behaviour change with reference to physiotherapists: its multiphase development and validation. Physiother Theory Pract. 2015;31:231–43. 10.3109/09593985.2014.994250. 25533133 10.3109/09593985.2014.994250

[17] Lowe A, Gee M, McLean S, Littlewood C, Lindsay C, Everett S. Physical activity promotion in physiotherapy practice: a systematic scoping review of a decade of literature. Br J Sports Med. 2018;52:122–7. 10.1136/bjsports-2016-096735. 28003241 10.1136/bjsports-2016-096735

[18] Colquhoun HL, Levac D, O’Brien KK, Straus S, Tricco AC, Perrier L, et al. Scoping reviews: time for clarity in definition, methods, and reporting. J Clin Epidemiol. 2014;67:1291–4. 10.1016/j.jclinepi.2014.03.013. 25034198 10.1016/j.jclinepi.2014.03.013

[19] Tricco AC, Lillie E, Zarin W, O’Brien KK, Colquhoun H, Levac D, et al. PRISMA extension for scoping reviews (PRISMA-ScR): checklist and explanation. Ann Intern Med. 2018;169:467–73. 10.7326/M18-0850. 30178033 10.7326/M18-0850

[20] Ouzzani M, Hammady H, Fedorowicz Z, Elmagarmid A. Rayyan-a web and mobile app for systematic reviews. Syst Rev. 2016;5:210. 10.1186/s13643-016-0384-4. 27919275 10.1186/s13643-016-0384-4 PMC5139140

[21] Hoffmann TC, Glasziou PP, Boutron I, Milne R, Perera R, Moher D, et al. Better reporting of interventions: template for intervention description and replication (TIDieR) checklist and guide. BMJ. 2014;348:g1687. 10.1136/bmj.g1687. 24609605 10.1136/bmj.g1687

[22] Geidl W, Sudeck G, Wais J, Pfeifer K. Bewegungsförderliche bewegungstherapie in der medizinischen rehabilitation: Konsequenzen der bundesweiten Bestandsaufnahme für die qualitätsentwicklung [Physical Activity Promotion in Exercise Therapy in Medical rehabilitation: Consequences of the Nationwide Survey for Quality Development]. Rehabilitation (Germany). 2022;61:336–43. 10.1055/a-1693-8380. 10.1055/a-1693-8380 34933356

[23] Michie S, Richardson M, Johnston M, Abraham C, Francis J, Hardeman W, et al. The behavior change technique taxonomy (v1) of 93 hierarchically clustered techniques: Building an international consensus for the reporting of behavior change interventions. Ann Behav Med. 2013;46:81–95. 10.1007/s12160-013-9486-6. 23512568 10.1007/s12160-013-9486-6

[24] Mayring P. Qualitative inhaltsanalyse. Grundlagen und techniken [Qualitative content analysis. Basics and techniques]. Weinheim und Basel: Beltz; 2008.

[25] Fruchterman TM, Reingold EM. Graph drawing by force-directed placement. Softw Pract Exp. 1991;21:1129–64. 10.1002/spe.4380211102.

[26] Cherven K. Network graph analysis and visualization with gephi: visualize and analyze your data swiftly using dynamic network graphs built with gephi. Birmingham: Packt Publishing; 2013.

[27] Adams DJ, Remick RA, Davis JC, Vazirian S, Khan KM. Exercise as medicine—the use of group medical visits to promote physical activity and treat chronic moderate depression: a preliminary 14-week re–post study. BMJ Open Sport Exerc Med. 2015. 10.1136/bmjsem-2015-. 10.1136/bmjsem-2015-000036 PMC5117054 27900130

[28] Alanko L, Laukkanen JA, Rottensteiner M, Rasmus S, Kuha T, Valtonen M, et al. Sports and exercise medicine clinic in public hospital settings: A real-life concept and experiences of the treatment of the first 1151 patients. Postgrad Med. 2022;123:283–9. 10.1101/2022.06.13.22276140. 10.1080/00325481.2022.2135894 36254719

[29] Borg S, Öberg B, Nilsson L, Söderlund A, Bäck M. The role of a behavioural medicine intervention in physiotherapy for the effects of rehabilitation outcomes in exercise-based cardiac rehabilitation (ECRA) - the study protocol of a randomised, controlled trial. BMC Cardiovasc Disord. 2017;17:134. 10.1186/s12872-017-0557-7. 28545400 10.1186/s12872-017-0557-7 PMC5445354

[30] Brauer SG, Lamont RM, O’Sullivan JD. A physiotherapy group exercise and self-management approach to improve physical activity in people with mild-moderate Parkinson’s disease: a randomized controlled trial. Trials. 2024;25:76. 10.1186/s13063-023-07870-4. 38254229 10.1186/s13063-023-07870-4 PMC10801959

[31] Bricca A, Jäger M, Dideriksen M, Rasmussen H, Nyberg M, Pedersen JR, et al. Personalised exercise therapy and self-management support for people with Multimorbidity: development of the MOBILIZE intervention. Pilot Feasibility Stud. 2022;8:244. 10.1186/s40814-022-01204-y. 36461048 10.1186/s40814-022-01204-y PMC9717541

[32] Casey M-B, Smart K, Segurado R, Hearty C, Gopal H, Lowry D, et al. Exercise combined with acceptance and commitment therapy (ExACT) compared to a supervised exercise programme for adults with chronic pain: study protocol for a randomised controlled trial. Trials. 2018;19:194. 10.1186/s13063-018-2543-5. 29566744 10.1186/s13063-018-2543-5 PMC5865382

[33] Cheng SW, Alison J, Stamatakis E, Dennis S, McNamara R, Spencer L, et al. Six-week behaviour change intervention to reduce sedentary behaviour in people with chronic obstructive pulmonary disease: a randomised controlled trial. Thorax. 2022;77:231–8. 10.1136/thoraxjnl-2020-214885. 34226203 10.1136/thoraxjnl-2020-214885

[34] Christiansen MB, Thomas L, Master H, Schmitt LA, Pohlig R, White DK. A physical therapist–administered physical activity intervention after total knee replacement: protocol for a randomized controlled trial. Phys Ther. 2018;98:578–84. 29608733 10.1093/ptj/pzy037 PMC6692704

[35] Christie AJ, Powers-James C, Narayanan S, Chen M, Eddy C, Gomez T, et al. Multidisciplinary lifestyle modification program (IM-FIT) for cancer survivors: implementation of a reimbursable model in a cancer hospital. Support Care Cancer. 2021;29:7365–75. 10.1007/s00520-021-06305-7. 34050398 10.1007/s00520-021-06305-7

[36] Cramp F, Thomas R, Haase AM, Domaille M, Manns S, Swales C, et al. Promoting engagement in physical activity in early rheumatoid arthritis: A proof-of-concept intervention study. Musculoskelet Care. 2020;18:487–500. 10.1002/msc.1493. 10.1002/msc.1493 32666652

[37] Daley AJ, Mutrie N, Crank H, Coleman R, Saxton J. Exercise therapy in women who have had breast cancer: design of the Sheffield women’s exercise and well-being project. Health Educ Res. 2004;19:686–97. 10.1093/her/cyg094. 15198998 10.1093/her/cyg094

[38] Elsman EB, Leerlooijer JN, Beek J ter, Duijzer G, Jansen SC, Hiddink GJ et al. Using the intervention mapping protocol to develop a maintenance programme for the SLIMMER diabetes prevention intervention. *BMC Public Health* (2014) 12. 10.1186/1471-2458-14-1108 10.1186/1471-2458-14-1108 PMC4286928 25346512

[39] Focht BC, Brawley LR, Rejeski WJ, Ambrosius WT. Group-mediated activity counseling and traditional exercise therapy programs: effects on health-related quality of life among older adults in cardiac rehabilitation. Ann Behav Med. 2004;28:52–61. 15249259 10.1207/s15324796abm2801_7

[40] Foster J, Thompson K, Harkin J. *Lets Get Moving - Commissioning guidance. A physical activtity care pathway* (2012) [cited 2025 Jan 16]. Available from: https://assets.publishing.service.gov.uk/media/5a7ba2a440f0b62826a04d73/dh_133101.pdf

[41] Groen WG, ten Tusscher MR, Verbeek R, Geleijn E, Sonke GS, Konings IR, et al. Feasibility and outcomes of a goal-directed physical therapy program for patients with metastatic breast cancer. Support Care Cancer. 2021;29:3287–98. 10.1007/s00520-020-05852-9. 10.1007/s00520-020-05852-9 33104921

[42] Hassett L, Jennings M, Brady B, Pinheiro M, Haynes A, Sidhu B, et al. Brief physical activity counselling by physiotherapists (BEHAVIOUR): protocol for an effectiveness-implementation hybrid type II cluster randomised controlled trial. Implement Sci Commun. 2022;3:39. 10.1186/s43058-022-00291-5. 35395933 10.1186/s43058-022-00291-5 PMC8991667

[43] Helmink JH, Meis JJ, de Weerdt I, Visser FN, De Vries NK, Kremers SP. Development and implementation of a lifestyle intervention to promote physical activity and healthy diet in the Dutch general practice setting: the BeweegKuur programme. Int J Behav Nutr Phys Act. 2010;7. 10.1186/1479-5868-7-49. 10.1186/1479-5868-7-49 PMC3224934 20504352

[44] Hilberdink B, van der Giesen F, Vliet Vlieland T, Nijkamp M, van Weely S. How to optimize exercise behavior in axial spondyloarthritis? Results of an intervention mapping study. Patient Educ Couns. 2020;103:952–9. 10.1016/j.pec.2019.12.017. 31926668 10.1016/j.pec.2019.12.017

[45] Johannesen G, Damlund AR, Grundtvig Vinter S, Spuur HS, Sarkez-Knudsen M, Thomsen TG. First step to empowering change: enhancing self-efficacy, energy management, and physical activity in patients with sleep apnea. Front Rehabil Sci. 2024;5:1359371. 10.3389/fresc.2024.1359371. 39071773 10.3389/fresc.2024.1359371 PMC11272648

[46] Johnson M-C, Judah G, Cunningham D, Olander EK. Individualised physical activity and physiotherapy behaviour change intervention tool for breast cancer survivors using self-efficacy and COM-B: feasibility study. Europ J Physiother. 2022;24:119–28. 10.1080/21679169.2020.1804616.

[47] Jones TM, Dear BF, Hush JM, Titov N, Dean CM. Application of intervention mapping to the development of a complex physical therapist intervention. Phys Ther. 1994;96. 10.2522/ptj.20150387. 10.2522/ptj.20150387 27256070

[48] Knittle K, de Gucht V, Hurkmans E, Peeters A, Ronday K, Maes S, et al. Targeting motivation and self-regulation to increase physical activity among patients with rheumatoid arthritis: a randomised controlled trial. Clin Rheumatol. 2015;34:231–8. 10.1007/s10067-013-2425-x. 24213780 10.1007/s10067-013-2425-x

[49] Labie C, Runge N, Mairesse O, Nijs J, Malfliet A, Verschueren S, et al. Integration of cognitive behavioral therapy for insomnia in best-practice care for patients with knee osteoarthritis and insomnia: a randomized controlled trial protocol. Phys Ther. 2024;104. 10.1093/ptj/pzad181. 10.1093/ptj/pzad181 38157312

[50] Laitakari J, Asikainen T-M. How to promote physical activity through individual counseling — A proposal for a practical model of counseling on health-related physical activity. Patient Educ Couns. 1998;33:S13–24. 10889742 10.1016/s0738-3991(98)00005-6

[51] Larkin L, Gallagher S, Fraser A, Kennedy N. Community-based intervention to promote physical activity in rheumatoid arthritis (CIPPA-RA): a study protocol for a pilot randomised control trial. Rheumatol Int. 2017;37:2095–103. 10.1007/s00296-017-3850-y. 29043493 10.1007/s00296-017-3850-y

[52] Larkin L, McKenna S, Pyne T, Comerford P, Moses A, Folan A, et al. Promoting physical activity in rheumatoid arthritis through a physiotherapist led behaviour change-based intervention (PIPPRA): a feasibility randomised trial. Rheumatol Int. 2024;44:779–93. 10.1007/s00296-024-05544-1. 38438576 10.1007/s00296-024-05544-1 PMC10980645

[53] Louie J, Baquie K, Offerman J, Granger CL, Khan F, Bower KJ. Maximising abilities, negotiating and generating exercise options (MANAGE) in people with multiple sclerosis: a feasibility randomised controlled trial. Clin Rehabil. 2022;36:498–510. 10.1177/02692155211064949. 34881669 10.1177/02692155211064949

[54] Ma JK, West CR, Martin Ginis KA. The effects of a patient and provider co-developed, behavioral physical activity intervention on physical activity, psychosocial predictors, and fitness in individuals with spinal cord injury: a randomized controlled trial. Sports Med. 2019;49:1117–31. 10.1007/s40279-019-01118-5. 31119717 10.1007/s40279-019-01118-5

[55] Manning VL, Hurley MV, Scott DL, Coker B, Choy E, Bearne LM. Education, self-management, and upper extremity exercise training in people with rheumatoid arthritis: a randomized controlled trial. Arthritis Care Res (Hoboken). 2014;66:217–27. 10.1002/acr.22102. 23925924 10.1002/acr.22102

[56] Moore CL, Kaplan SL. A framework and resources for shared decision making: opportunities for improved physical therapy outcomes. Phys Ther. 2018;98. 10.1093/ptj/pzy095. 10.1093/ptj/pzy095 30452721

[57] Moore SA, Avery L, Price CI, Flynn D. A feasibility, acceptability and fidelity study of a multifaceted behaviour change intervention targeting free-living physical activity and sedentary behaviour in community dwelling adult stroke survivors. Pilot Feasibility Stud. 2020;6:58. 10.1186/s40814-020-00603-3. 32368348 10.1186/s40814-020-00603-3 PMC7189695

[58] Morris JH, Irvine LA, Dombrowski SU, McCormack B, van Wijck F, Lawrence M. We walk: a person-centred, dyadic behaviour change intervention to promote physical activity through outdoor walking after stroke-an intervention development study. BMJ Open. 2022;12:e058563. 10.1136/bmjopen-2021-058563. 35701066 10.1136/bmjopen-2021-058563 PMC9198706

[59] Motl RW, Lein DH, Morris DM, Lowman JD, Perez P, Bullard C. Behavior change interventions for health promotion in physical therapist research and practice: an integrative approach. Phys Ther. 2022;102. 10.1093/ptj/pzab266. 10.1093/ptj/pzab266 34935964

[60] O’Dwyer T, Monaghan A, Moran J, O’Shea F, Wilson F. Behaviour change intervention increases physical activity, spinal mobility and quality of life in adults with ankylosing spondylitis: a randomised trial. J Physiotherapy. 2017;63:30–9. 10.1016/j.jphys.2016.11.009. 10.1016/j.jphys.2016.11.009 27989730

[61] Pollock KM. Exercise in treating depression: broadening the psychotherapist’s role. J Clin Psychol. 2001;57:1289–300. 10.1002/jclp.1097. 11590615 10.1002/jclp.1097

[62] Quinn L, Trubey R, Gobat N, Dawes H, Edwards RT, Jones C, et al. Development and delivery of a physical activity intervention for people with huntington disease: facilitating translation to clinical practice. J Neurol Phys Ther. 2016;40:71–80. 10.1097/NPT.0000000000000119. 26863152 10.1097/NPT.0000000000000119 PMC4795097

[63] Reid RD, Morrin LI, Higginson LA, Wielgosz A, Blanchard C, Beaton LJ, et al. Motivational counselling for physical activity in patients with coronary artery disease not participating in cardiac rehabilitation. Eur J Prev Cardiol. 2012;19:161–6. 10.1177/1741826711400519. 21450579 10.1177/1741826711400519

[64] Richmond H, Lait C, Srikesavan C, Williamson E, Moser J, Newman M, et al. Development of an exercise intervention for the prevention of musculoskeletal shoulder problems after breast cancer treatment: the prevention of shoulder problems trial (UK PROSPER). BMC Health Serv Res. 2018;18:463. 10.1186/s12913-018-3280-x. 29914494 10.1186/s12913-018-3280-x PMC6006920

[65] Ryan JM, Fortune J, Stennett A, Kilbride C, Anokye N, Victor C, et al. Changing physical activity behaviour for people with multiple sclerosis: protocol of a randomised controlled feasibility trial (iStep-MS). BMJ Open. 2017;7:e018875. 10.1136/bmjopen-2017-018875. 29146660 10.1136/bmjopen-2017-018875 PMC5695400

[66] Salmon VE, Hewlett S, Walsh NE, Kirwan JR, Morris M, Urban M, et al. Developing a group intervention to manage fatigue in rheumatoid arthritis through modifying physical activity. BMC Musculoskelet Disord. 2019;20:194. 10.1186/s12891-019-2558-4. 31054567 10.1186/s12891-019-2558-4 PMC6500086

[67] Saxton JM, Carter A, Daley AJ, Snowdon N, Woodroofe MN, Petty J, et al. Pragmatic exercise intervention for people with multiple sclerosis (ExIMS trial): study protocol for a randomised controlled trial. Contemp Clin Trials. 2013;34:205–11. 10.1016/j.cct.2012.10.011. 23123791 10.1016/j.cct.2012.10.011

[68] Stevens A, Köke A, van der Weijden T, Beurskens A. The development of a patient-specific method for physiotherapy goal setting: a user-centered design. Disabil Rehab. 2018;40:2048–55. 10.1080/09638288.2017.1325943. 10.1080/09638288.2017.1325943 28504014

[69] Sudeck G, Höner O. Volitional interventions within cardiac exercise therapy (VIN-CET): long-term effects on physical activity and health-related quality of life. Appl Psychol Health Well-Being. 2011;3:151–71. 10.1111/j.1758-0854.2010.01047.x.

[70] Tatta J, Willgens AM, Palombaro KM. Mindfulness and acceptance-based interventions in physical therapist practice: the time is now. Phys Ther. 2022;102. 10.1093/ptj/pzab293. 10.1093/ptj/pzab293 35079796

[71] Teuwen MM, van Weely SF, Vliet Vlieland TP, van Wissen MA, Peter WF, Broeder AA den, et al. Effectiveness of longstanding exercise therapy compared with usual care for people with rheumatoid arthritis and severe functional limitations: a randomised controlled trial. Ann Rheum Dis. 2024;83:437–45. 10.1136/ard-2023-224912. 10.1136/ard-2023-224912 38171602

[72] van der Ploeg HP, Streppel KR, van der Beek AJ, van der Woude LH, Vollenbroek-Hutten MM, van Harten WH, et al. Counselling increases physical activity behaviour nine weeks after rehabilitation. Br J Sports Med. 2006;40:223–9. 10.1136/bjsm.2005.021139. 16505078 10.1136/bjsm.2005.021139 PMC2491980

[73] van Grootel J, Bor P, Veenhof C, Valkenet K. Development of a goal-directed movement intervention (GOAL) using a movement sensor for hospitalized patients: an intervention mapping approach. Clin Rehabil. 2024;38:251–62. 10.1177/02692155231198173. 37644843 10.1177/02692155231198173 PMC10725127

[74] van Nimwegen M, Speelman AD, Smulders K, Overeem S, Borm GF, Backx FJ, et al. Design and baseline characteristics of the parkfit study, a randomized controlled trial evaluating the effectiveness of a multifaceted behavioral program to increase physical activity in Parkinson patients. BMC Neurol. 2010. 10.1186/1471-2377-10-70. 20723221 10.1186/1471-2377-10-70 PMC2936282

[75] Willett M, Greig C, Fenton S, Rogers D, Duda J, Rushton A. Utilising the perspectives of patients with lower-limb osteoarthritis on prescribed physical activity to develop a theoretically informed physiotherapy intervention. BMC Musculoskelet Disord. 2021;22:155. 10.1186/s12891-021-04036-8. 33557821 10.1186/s12891-021-04036-8 PMC7871381

[76] Willett M, Rushton A, Stephens G, Fenton S, Rich S, Greig C, et al. Feasibility of a theoretically grounded, multicomponent, physiotherapy intervention aiming to promote autonomous motivation to adopt and maintain physical activity in patients with lower-limb osteoarthritis: protocol for a single-arm trial. Pilot Feasibility Stud. 2023;9:54. 10.1186/s40814-023-01274-6. 37004124 10.1186/s40814-023-01274-6 PMC10064730

[77] Wittink H, van Gessel C, Outermans J, Blatter T, Punt M, van der Lugt R. Co-design of a walking activity intervention for stroke survivors. Front Rehabil Sci. 2024;5:1369559. 10.3389/fresc.2024.1369559. 38894717 10.3389/fresc.2024.1369559 PMC11183812

[78] Wolf S, Seiffer B, Zeibig J-M, Welkerling J, Bauer LL, Frei AK, et al. Efficacy and cost-effectiveness of a transdiagnostic group-based exercise intervention: study protocol for a pragmatic multi-site randomized controlled trial. BMC Psychiatry. 2021;21:540. 10.1186/s12888-021-03541-3. 34717567 10.1186/s12888-021-03541-3 PMC8556805

[79] Jung A, Geidl W, Matting L, Hoessel L-M, Siemens W, Sudeck G, et al. Efficacy of physical activity promoting interventions in physical therapy and exercise therapy for persons with noncommunicable diseases: an overview of systematic reviews. Phys Ther. 2024;104. 10.1093/ptj/pzae053. 10.1093/ptj/pzae053 38564265

[80] Ekkekakis P, Zenko Z, Vazou S. Do you find exercise pleasant or unpleasant? The affective exercise experiences (AFFEXX) questionnaire. Psychol Sport Exerc. 2021;55:101930. 10.1016/j.psychsport.2021.101930.

[81] Ekkekakis P, Brand R. Affective responses to and automatic affective valuations of physical activity: Fifty years of progress on the seminal question in exercise psychology. Psychol Sport Exerc. 2019;42:130–7. 10.1016/j.psychsport.2018.12.018.

[82] Brand R, Ekkekakis P. Affective–Reflective theory of physical inactivity and exercise. Ger J Exerc Sport Res. 2018;48:48–58. 10.1007/s12662-017-0477-9.

[83] Sudeck G, Pfeifer K. Physical activity-related health competence as an integrative objective in exercise therapy and health sports – conception and validation of a short questionnaire. Sportwiss. 2016;46:74–87. 10.1007/s12662-016-0405-4.

[84] Carl J, Sudeck G, Pfeifer K. Competencies for a healthy physically active lifestyle-reflections on the model of physical Activity-Related health competence. J Phys Act Health. 2020;17:688–97. 10.1123/jpah.2019-0442. 32473589 10.1123/jpah.2019-0442

