# Correction: Physical activity promotion in physical therapy, exercise therapy and other movement-based therapies: a scoping review and content analysis of intervention studies and theoretical works

**DOI:** 10.1186/s12966-025-01803-x

**Published:** 2025-07-25

**Authors:** Leon Matting, Klaus Pfeifer, Gorden Sudeck, Andrés Jung, Florian Langhirt, Wolfgang Geidl

**Affiliations:** 1 Education & Health Research, Institute of Sports Science, Faculty of Economics and Social Sciences, University of Tübingen, Wächterstraße 67, Tübingen, 72074 Germany; 2 Department of Sport Science and Sport, Friedrich-Alexander-Universität Erlangen-Nürnberg, Gebbertstraße 123b, 91058 Erlangen, Germany

**Correction to: Matting et al. International Journal of Behavioral Nutrition and Physical Activity (2025) 22:72**

10.1186/s12966-025-01772-1

Following the publication of the original article, the authors reported that the legend of Fig. 3 was mistakenly processed as part of the main text during typesetting. The correct legend for Fig. 3 should read:

*The network visualization of behavior change techniques (BCTs) and didactic-methodological principles (DMPs; highlighted with a black border) was generated via the Fruchterman-Reingold algorithm*,* which clusters techniques based on their connectivity. Node size reflects degree centrality*,* with larger nodes indicating greater interconnectedness. Node color ranges from red (low weighted degree) to blue (high weighted degree). To highlight stronger or multiple associations*,* single (simple) connections between techniques were excluded from the visualization. Note: For improved readability*,* some BCTs and DMPs have been abbreviated. The corresponding full terms are as follows: Action = Action planning; Adding objects = Adding objects to the environment; Contract = Behavioral contract; Demonstration = Demonstration of the behavior; Discrepancy = Discrepancy between current behavior and goal; Experiences = Facilitating positive movement experiences*,* enjoyment of physical activity; Feedback = Feedback on behavior; Feedback (outcomes) = Feedback on outcome(s) of behavior; Framing = Framing/reframing; Generalization = Generalization of target behavior; Goal setting = Goal setting (behavior); Info (antecedents) = Information about antecedents; Info (health) = Information about health consequences; Info (social & environment) = Information about social and environmental consequences; Instruction = Instruction on how to perform the behavior; Interaction = consideration of interaction and language / collaborative communication; Material reward = Material reward (behavior); Participation = Active participation in shaping the therapy process; Past success = Focus on past success; Pharmacological = Pharmacological support; Practice = Behavioral practice/rehearsal; Problem = Problem solving; Reduce = Reduce negative emotions; Restructuring (physical) = Restructuring the physical environment; Restructuring (social) = Restructuring the social environment; Review goals = Review behavior goal(s); Review goals (outcome) = Review outcome goal(s); Salience = Salience of consequences; Self-monitoring: Self-monitoring of behavior; Self-monitoring (outcomes) = Self-monitoring of outcome(s) of behavior; Self-responsibility = Patients’ self-responsibility and independence; Support (emo) = Social support (emotional); Support (unspec) = Social Support unspecified; Substitution = Behavior Substitution; Tailoring = Tailoring and individualization; Utilizing group settings = Utilizing group settings.*

In addition, the citations in Table 3 had not yet been updated to reflect the Reference List. The corrected version of Table 3 is as follows:

**Table 3:** Summary of assessment categories and related measuring instruments

(Sub-)Category and definition	Number of PAP concepts reporting	Measuring instruments (if reported)
**Physical Activity Behavior (Outcome)** This category includes all measurements related to current movement behavior, encompassing the frequency, intensity, duration, and types of physical activities performed by an individual (e.g. questionnaire or accelerometer/pedometer). This also includes all forms of self-monitoring related to physical activity.	*n* = 34	Device-based measurement ActiGraph GT1M (67); ActiGraph GT3 × (29, 34); ActiGraph GT9 × (54); ActivPAL3 micro (33); Fitbit Zip Pedometer (27, 32); FitBit Charge 5 (45); Garmin Vivosmart fitness tracker (30); Physical Activity Monitor (PAM AM 400) (73); Onmood Pedometer (71); Yamax Pedometer (78); Yamax SW-200 Digi-walker Pedometer (65); Digi-walkers (New Lifestyles, Inc.) (39); TracmorD Triaxial Accelerometer (74) Questionnaires General Practitioner Physical Activity Questionnaire (GPPAQ) (40); Godin-Shepard Leisure-Time Exercise Questionnaire (GLTEQ) (46); LASA Physical Activity Questionnaire (LAPAQ) (15); Leisure Time PA Questionnaire (LTPAQ) (54); Sedentary Behaviour Questionnaire (33); Short Questionnaire to Assess Health-Enhancing Physical Activity (48) Others Diary (29); Interview (based on COM-B) (42)
**Previous physical activity experiences** This category includes all measurements related to the sport or exercise history.	*n* = 4	Informal survey through conversation (11, 62, 63, 72)
**Other health-related behaviors** This category includes all measurements related to other health-related behaviors (e.g. dietary behavior or smoking status).	*n* = 6	Lifestyle Questionnaire (28); Informal survey through conversation (50, 63)
**Physical capabilities & skills** This category includes all measurements related to physical capabilities and skills to engage in the activity-related behavior (e.g. strength, mobility or balance).	*n* = 13	Six-minute walk test (15, 28, 34, 41, 45, 53); Stress ECG-test (28); Sit-ups form a chair (28); Timed Up & Go Test (TUG) (15, 41); 5 times sit to stand (5TSTST) (41); Squats (28); Hand-grip Strength (15, 28); One repetition maximum—lower extremities (1RM-LE) (41); One repetition maximum—upper extremities (1RM-UE) (41); One-leg stance (28); Berg Balance Scale (BBS) (15); Short physical performance battery (SPPB) (41); Range Of Motion (ROM) (15, 64); Functional Reach (53); Rivermead Mobility Index (57); Interview (based on COM-B)(33); Activity of daily living (ADL) (45)
**Psychological capabilities & skills** This category includes all measurements related to psychological capability and skills to engage in the activity-related behavior (e.g. cognitions, beliefs, expectations, fear, self-efficacy etc.).	*n* = 18	Barrier and Task Self Efficacy Scale (46); Exercise Benefits Barriers Scale (53); Confidence scale (64); Fear of Movement Scale for Osteoarthritis questionnaire (FMSO) (34); Chronic Pain Acceptance Questionnaire (CPAQ) (70); Interview (based on COM-B) (33)
**Additional psychological factors** This category includes all measurements related to additional psychological factors that are not directly related to the activity-related behavior (e.g. depression, well-being, mindfulness etc.)	*n* = 5	Warwick-Edinburgh Mental Well-Being Scale (57); Generalized Anxiety Disorder Questionnaire (GAD-7) (35); Distress Thermometer (35); Patient Health Questionnaire (PHQ-9) (35); Five Facets of Mindfulness (70); State Mindfulness Scale for Physical Activity (70); Valued living questionnaire (VLQ) (70)
**Biomedical influencing factors** This category includes all measurements related to biomedical deficits, such as disease and tissue pathology, as well as health status indicators such as BMI and overall health metrics.	*n* = 19	Numerical pain rating scale (NPRS) (41); Multidimensional fatigue inventory (MFI) (41); Visual analog scale for fatigue (VASF) (41); Abbreviated fatigue questionnaire (AFQ) (41); Fatigue Severity Scale (53); Multiple Sclerosis Impact Scale (MSIS-29) (53); Rheumatoid Arthritis Disease Activity Index (RADAI) (48); Electrocardiogram (ECG) (28); Knee Outcome Scale (KOS) (34); Disability of Arm Shoulder and Hand (DASH) (46); Hip Disability and Osteoarthritis Out-come Score (HOOS) (56); Western Ontario & McMaster Universities Osteoarthritis Index (WOMAC) (46); Medical Outcomes Study (MOS) (34); 12-item Short Form Survey (SF-12) (56); Tanita^®^ scale (Body weight; percent body fat) (35)
**Social and physical opportunities** This category includes both physical and social opportunities, encompassing the environmental and social factors that influence an individual’s behavior.	*n* = 9	Interview (based on COM-B) (33)
**Others** This category includes all factors that could not be covered by other categories, e.g., quality of life.	*n* = 9	Quality of life Questionnaire (RAND-36) (28); Patient-Specific Complaints (PSC) (15); Canadian Occupational Performance Measure (COPM) (45)

The original article has been updated accordingly.

