# Supplementary material for: Physical activity promotion in physical therapy, exercise therapy and other movement-based therapies: a scoping review and content analysis of intervention studies and theoretical works
Source: Int J Behav Nutr Phys Act. 2025 Jun 10;22:72. doi: 10.1186/s12966-025-01772-1 (PMC12153217; doi:10.1186/s12966-025-01772-1)
Supplement: Supplementary file 1 — Supplementary Material 1: Additional file 1 provides details on deviations from the preregistered protocol, eligibility criteria search strategies, and a list of excluded studies with reasons for exclusion. [file 12966_2025_1772_MOESM1_ESM.docx]

**Physical activity promotion in physical therapy, exercise therapy and other movement-based therapies: A scoping review and content analysis of interventional concepts**

**Additional file 1**

| **Table or Figure** | **Page** |
| --- | --- |
| Table S1: Deviations from preregistered protocol | 1 |
| Table S2: Eligibility criteria | 2 |
| Table S3: Search strategies | 3-4 |
| Table S4: Excluded studies and reasons for exclusion | 5-26 |

**Table S1**

| **Deviations from preregistered protocol** | |
| --- | --- |
| Eligibility criteria | During data extraction, we found that some reports (RCTs in most cases) did not describe the intervention concept in great detail, nor did they cite other publications that described the intervention concept. Therefore, there was not enough information to extract and perform the mapping and content analysis. We used the TIDIER checklist (Hoffmann et al. 2016) to assess whether a report (as described above) contained sufficient information about the intervention concept. Two reviewers assessed each of these reports individually and after consensus was reached, a decision was made to exclude or include the respective report. If the report did not provide sufficient information on the intervention concept (and thus no relevant information could be extracted according to the categories described below for the content analysis), the report was excluded from analysis. Additionally, there were concepts that included elements of promoting physical activity, but physical activity was not the primary focus of these concepts. Rather, physical activity was only used as a means to address pain management or disease-specific treatment. These concepts, which primarily focused on pain reduction, symptom management, or specific disease treatments rather than on promoting physical activity, were also excluded from the analysis. Concepts that focused exclusively on remote or e-health interventions were not included as well. |
| Mapping synthesis | It was planned to extract and map the Data using a deductive content analysis method. The categories to be mapped refer to the therapeutic core elements according to the kybernetic model (assessment; therapy goals; contents and methodology; method of operation; therapy implementation; and therapy monitoring) and 2) to quality categories of a movement-promoting movement therapy following Geidl et al. 2022, e.g. interdisciplinarity, standardisation and manualisation, patient orientation, and theory-based. During the inductive process, we realized that it makes sense to expand methods of operation as didactic-methodical principles and to integrate therapy monitoring into the area of assessment. In addition, due to the poor reporting of the existing concepts, we decided against mapping the quality categories of physical activity promoting movement therapy. |

**References**

Geidl, W., Sudeck, G., Wais, J., & Pfeifer, K. (2022). Bewegungsförderliche Bewegungstherapie in der medizinischen Rehabilitation: Konsequenzen der bundesweiten Bestandsaufnahme für die Qualitätsentwicklung. [Physical Activity Promotion in Exercise Therapy in Medical Rehabilitation: Consequences of the Nationwide Survey for Quality Development]. *Die Rehabilitation*, *61*(5), 336–343. https://doi.org/10.1055/a-1693-8380

Hoffmann, T. C., Glasziou, P. P., Boutron, I., Milne, R., Perera, R., Moher, D., Altman, D. G., Barbour, V., Macdonald, H., Johnston, M., Lamb, S. E., Dixon-Woods, M., McCulloch, P., Wyatt, J. C., Chan, A. W., & Michie, S. (2016). Die TIDieR Checkliste und Anleitung – ein Instrument für eine verbesserte Interventionsbeschreibung und Replikation [Better Reporting of Interventions: Template for Intervention Description and Replication (TIDieR) Checklist and Guide]. *Gesundheitswesen (Bundesverband der Arzte des Offentlichen Gesundheitsdienstes (Germany))*, *78*(3), e174. https://doi.org/10.1055/s-0037-1600948

**Table S2 Eligibility criteria**

| Criteria | Inclusion criteria | Exclusion criteria |
| --- | --- | --- |
| Target population | - Adult population with non-communicable diseases. | - Patients with communicable diseases.  - non-adults (age < 18 years). |
| Physical activity promotion concept | - Concepts, which aim to promote physical activity in movement-based therapy. | - exercise therapy or physical therapy that is directed towards improving body functions and structures.  - not providing enough information (TIDIER).  - being focused on pain issues or on specific disease treatments.  - remote or e-health intervention. |
| Context | - Our main setting of interest is the health care setting with a focus on rehabilitation setting.  - Other settings will be considered if they are applicable to the main setting of interest. | Settings not applicable to the main setting of interest. |

**Table S3 Search strategies**

**Pubmed**

| Search strategy for Pubmed | |
| --- | --- |
| #1 | (physiotherap*[tiab] OR "physical therap*"[tiab] OR "exercise therap*"[tiab] OR "movement therap*"[tiab] OR "sport therap*"[tiab] OR "sports therap*"[tiab] OR “training therapy”[tiab] OR "Physical Therapists"[Mesh]) |
| #2 | ("Health Education"[Mesh] OR "Health Promotion"[Mesh] OR "Health Literacy"[Mesh] OR "Behavior Therapy"[Mesh] OR "Cognitive Behavioral Therapy"[Mesh] OR "Motivational Interviewing"[Mesh] OR "motivational therap*"[tiab] OR "behaviour change*"[tiab] OR "behavior change*"[tiab] OR promoti*[tiab]) |
| #3 | ("physical activit*"[tiab] OR "healthy life-style"[tiab] OR "active lifestyle"[tiab] OR "Healthy Lifestyle"[Mesh] OR "Health Behavior"[Mesh] OR "Physical Fitness"[Mesh] OR "Treatment Adherence and Compliance"[Mesh] OR "Exercise Test"[Mesh]) |
| #4 | ("Child"[Mesh] OR "Infant"[Mesh] OR "Adolescent"[Mesh] OR school[tiab] OR “address”[Publication Type] OR “biography”[Publication Type] OR “case reports”[Publication Type] OR “comment”[Publication Type] OR “directory” [Publication Type] OR “editorial”[Publication Type] OR “festschrift”[Publication Type] OR “interview”[Publication Type] OR “lecture”[Publication Type] OR “legal case”[Publication Type] OR “legislation”[Publication Type] OR “letter”[Publication Type] OR “news”[Publication Type] OR “newspaper article”[Publication Type] OR “patient education handout”[Publication Type] OR “congress”[Publication Type] OR “consensus development conference”[Publication Type]) |
| #5 | (#1 AND #2 AND #3) NOT #4 |

**Web of Science**

| #1 | TI = ( physiotherap* OR "physical therap*" OR "exercise therap*" OR "movement therap*" OR "sports therap*" OR "sport therap*" OR "training therap*" ) |
| --- | --- |
| #2 | AB = ( physiotherap* OR "physical therap*" OR "exercise therap*" OR "movement therap*" OR "sports therap*" OR "sport therap*" OR "training therap*" ) |
| #3 | TS = ( "health promoti*" OR coaching OR "health educati*" OR "health literac*" OR "Motivational Interview*" OR "Behavioral Therap*" OR "Behavioural Therap*" OR "Behaviour Therap*" OR "Behavior Therap*" OR "Behavior change*" OR "Behaviour change*" OR "motivational therap*" OR promot*) |
| #4 | TS = ("physical activit*" OR "healthy life-style" OR "healthy lifestyle" OR “health behavior” OR “health behaviour" OR “active lifestyle" OR “active life-style" OR "Healthy Lifestyle*" OR "Physical Fitness") |
| #5 | TS = (child* OR minor OR adolescent OR school ) |
| #6 | ((#1 OR #2) AND #3 AND #4) NOT #5 |

**PsycINFO**

| #1 | ((exp *Health Care Delivery/ OR exp *Rehabilitation/ OR exp *Therapists/) AND exp *Exercise/ ) OR exp *Physical Therapists/ or exp *Movement Therapy/ OR  exp *Physical Therapy/ |
| --- | --- |
| #2 | (physiotherap* OR "physical therap*" OR "exercise therap*" OR "movement therap*" OR "sports therap*" OR "sport therap*" OR "training therap*" OR "trainings therap*").ti,ab |
| #3 | #1 OR #2 |
| #4 | exp *Health Education/ or exp *Health Promotion/ or exp *Behavior Therapy/ or exp *Behavior Change/ or exp *Motivational Interviewing/ or exp *Health Literacy/ |
| #5 | ("motivational therap*" OR "Motivational Interview*" OR "behaviour change*" OR "behavior change*" OR promoti*).ti,ab |
| #6 | #4 OR #5 |
| #7 | exp *Physical Activity/ or exp Treatment Compliance/ or exp *Health Behavior/ or exp *Physical Fitness/ or ("physical activity" or "healthy lifestyle" OR "active lifestyle" ).ti,ab |
| #8 | #3 AND #6 AND #7 |

**Scopus**

| ( TITLE-ABS ( physiotherap* OR "physical therap*" OR "exercise therap*" OR "movement therap*" OR "sports therap*" OR "sport therap*" OR "training therap*" ) AND TITLE-ABS-KEY ( ( "health promoti*" OR coaching OR "health educati*" OR "health literac*" OR "Motivational Interview*" OR "Behavioral Therap*" OR "Behavioural Therap*" OR "Behaviour Therap*" OR "Behavior Therap*" OR "Behavior change*" OR "Behaviour change*" OR "motivational therap*" OR promoti* ) AND ( "physical activit*" OR "healthy life-style" OR "healthy lifestyle" OR "health behavior" OR "health behaviour" OR "active lifestyle" OR "active life-style" OR "Physical Fitness" ) ) ) AND NOT TITLE-ABS-KEY ( child* OR minor OR adolescent OR school ) |
| --- |

**Table S4: Excluded studies and reasons for exclusion**

| **Studies** | **Reason(s) for exclusion** |
| --- | --- |

|  | Achten, J. P. J., Mooren-van der Meer, S., Pisters, M. F., Veenhof, C., Koppenaal, T., & Kloek, C. J. J. (2022). Self-management behaviour after a physiotherapist guided blended self-management intervention in patients with chronic low back pain: A qualitative study. Musculoskeletal Science and Practice, 62. https://doi.org/10.1016/j.msksp.2022.102675 | Exclusively e-health intervention |
| --- | --- | --- |
|  | Aittasalo, M., Raitanen, J., Kinnunen, T. I., Ojala, K., Kolu, P., & Luoto, R. (2012). Is intensive counseling in maternity care feasible and effective in promoting physical activity among women at risk for gestational diabetes? Secondary analysis of a cluster randomized NELLI study in Finland. The International Journal of Behavioral Nutrition and Physical Activity, 9(1). https://dx.doi.org/10.1186/1479-5868-9-104 | Concept already included |
|  | Akylbekov, A., Orme, M. W., Jones, A. V., Mademilov, M., Muratbekova, A., Aidaralieva, S., Mirzalieva, G., Oleinik, A., Magdieva, K., Taalaibekova, A., Rysbek Kyzy, A., Yusuf, Z. K., Rupert, J., Barton, A., Miah, R. B., Manise, A., Matheson, J. A., Malcolm, D., Free, R. C., … Singh, S. J. (2022). Culturally adapted pulmonary rehabilitation for adults living with post-tuberculosis lung disease in Kyrgyzstan: Protocol for a randomised controlled trial with blinded outcome measures. BMJ Open, 12(2). https://doi.org/10.1136/bmjopen-2021-048664 | TIDier (insufficient reporting) |
|  | Albaladejo, C., Kovacs, F. M., Royuela, A., del Pino, R., & Zamora, J. (2010). The efficacy of a short education program and a short physiotherapy program for treating low back pain in primary care: A cluster randomized trial. Spine, 35(5), 483–496. https://doi.org/10.1097/BRS.0b013e3181b9c9a7 | No PAP-concept |
|  | Albertini, S., Ciocca, A., Opasich, C., Pinna, G. D., & Cobelli, F. (2011). [Third phase of cardiac rehabilitation: A nurse-based „home-control“ model]. Monaldi archives for chest disease = Archivio Monaldi per le malattie del torace, 76(4), 168–174. https://doi.org/10.4081/monaldi.2011.173 | Wrong language |
|  | Alewijnse, D., Mesters, I. E. P. E., Metsemakers, J. F. M., & van den Borne Ilse E. P. E.; ORCID: https://orcid.org/0000-0001-8940-5271, B. H. W. A. I.-M. (2002). Program development for promoting adherence during and after exercise therapy for urinary incontinence. Patient Education and Counseling, 48(2), 147–160. https://dx.doi.org/10.1016/S0738-3991%2802%2900021-6 | No PAP-concept |
|  | Alewijnse, D., Metsemakers, J. F. M., Mesters, I. E. P. E., & van den Borne, B. (2003). Effectiveness of pelvic floor muscle exercise therapy supplemented with a health education program to promote long-term adherence among women with urinary incontinence. Neurourology and Urodynamics, 22(4), 284–295. https://doi.org/10.1002/nau.10122 | No PAP-concept |
|  | Ali, R., Siddiqi, M. H., Lee, S., & Kang, B. H. (2015). KARE: A hybrid reasoning approach for promoting active lifestyle. ACM IMCOM 2015 - Proceedings. https://doi.org/10.1145/2701126.2701156 | Exclusively e-health intervention |
|  | Allen, K. D., Bosworth, H. B., Brock, D. S., Chapman, J. G., Chatterjee, R., Coffman, C. J., Datta, S. K., Dolor, R. J., Jeffreys, A. S., Juntilla, K. A., Kruszewski, J., Marbrey, L. E., McDuffie, J., Oddone, E. Z., Sperber, N., Sochacki, M. P., Stanwyck, C., Strauss, J. L., & Yancy, W. S. J. (2012). Patient and provider interventions for managing osteoarthritis in primary care: Protocols for two randomized controlled trials. BMC Musculoskeletal Disorders, 13, 60–60. https://doi.org/10.1186/1471-2474-13-60 | Exclusively e-health intervention |
|  | Alston, S. D., & O’Sullivan, T. J. (2005). Patient education in physiotherapy of low back pain: Acute outcomes of group instruction. Irish Journal of Medical Science, 174(3), 64–69. https://doi.org/10.1007/BF03169151 | No PAP-concept |
|  | Amalakuhan, B., & Adams, S. G. (2015). Improving outcomes in chronic obstructive pulmonary disease: The role of the interprofessional approach. International Journal of Chronic Obstructive Pulmonary Disease, 10, 1225–1232. https://doi.org/10.2147/COPD.S71450 | No PAP-concept |
|  | Aminian, S., Motl, R. W., Rowley, J., & Manns, P. J. (2019). Management of multiple sclerosis symptoms through reductions in sedentary behaviour: Protocol for a feasibility study. BMJ OPEN, 9(4). https://doi.org/10.1136/bmjopen-2018-026622 | TIDier (insufficient reporting) |
|  | Andersen, M. R., Clausen, A., Sternhagen Nielsen, A. B., & Hjort Svendsen, A. L. (2021). Experiences with basic body awareness therapy as an add-on to cognitive behavioural therapy among Danish military veterans with PTSD: An interview study. Journal of Bodywork and Movement Therapies, 27, 550–559. https://doi.org/10.1016/j.jbmt.2021.03.023 | No PAP-concept |
|  | Andersen, R. M., Skou, S. T., Clausen, M. B., Jager, M., Zangger, G., Grontved, A., Brond, J. C., Soja, A. M. B., & Tang, L. H. (2022). Maintenance of physical activity after cardiac rehabilitation (FAIR): Study protocol for a feasibility trial. BMJ OPEN, 12(4). https://doi.org/10.1136/bmjopen-2021-060157 | Exclusively e-health intervention |
|  | Araya-Quintanilla, F., Gutierrez-Espinoza, H., Munoz-Yanez, M. J., Cavero-Redondo, I., Alvarez-Bueno, C., & Martinez-Vizcaino, V. (2020). Effectiveness of a multicomponent treatment versus conventional treatment in patients with fibromyalgia Study protocol. MEDICINE, 99(4). https://doi.org/10.1097/MD.0000000000018833 | No PAP-concept |
|  | Archer, K. R., Coronado, R. A., Haug, C. M., Vanston, S. W., Devin, C. J., Fonnesbeck, C. J., Aaronson, O. S., Cheng, J. S., Skolasky, R. L., Riley, L. H., & Wegener, S. T. (2014). A comparative effectiveness trial of postoperative management for lumbar spine surgery: Changing behavior through physical therapy (CBPT) study protocol. BMC MUSCULOSKELETAL DISORDERS, 15. https://doi.org/10.1186/1471-2474-15-325 | Focus mainly on pain and not on PAP |
|  | Arena, R., Ozemek, C., Laddu, D., Campbell, T., Rouleau, C. R., Standley, R., Bond, S., Abril, E. P., Hills, A. P., & Lavie, C. J. (2018). Applying Precision Medicine to Healthy Living for the Prevention and Treatment of Cardiovascular Disease. Current problems in cardiology, 43(12), 448–483. https://doi.org/10.1016/j.cpcardiol.2018.06.001 | Discussion paper |
|  | Arida, R. M. (2014). Impact of physical exercise therapy on behavioral and psychosocial aspects of epilepsy. Epilepsy and Behavior, 40, 90–91. https://doi.org/10.1016/j.yebeh.2014.08.031 | No PAP-concept |
|  | Arkkukangas, M., & Hultgren, S. (2019). Implementation of motivational interviewing in a fall prevention exercise program: Experiences from a randomized controlled trial. BMC Research Notes, 12(1). https://doi.org/10.1186/s13104-019-4309-x | Wrong setting |
|  | Asenlöf, P., Denison, E., & Lindberg, P. (2005). Individually tailored treatment targeting activity, motor behavior, and cognition reduces pain-related disability: A randomized controlled trial in patients with musculoskeletal pain. The Journal of Pain, 6(9), 588–603. https://doi.org/10.1016/j.jpain.2005.03.008 | Focus mainly on pain and not on PAP |
|  | Asenlöf, P., Denison, E., & Lindberg, P. (2005). Individually tailored treatment targeting motor behavior, cognition, and disability: 2 experimental single-case studies of patients with recurrent and persistent musculoskeletal pain in primary health care. Physical therapy, 85(10), 1061–1077. | Focus mainly on pain and not on PAP |
|  | Asenlöf, P., Denison, E., & Lindberg, P. (2006). Idiographic outcome analyses of the clinical significance of two interventions for patients with musculoskeletal pain. Behaviour Research and Therapy, 44(7), 947–965. https://doi.org/10.1016/j.brat.2005.07.005 | Focus mainly on pain and not on PAP |
|  | Askim, T., Langhammer, B., Ihle-Hansen, H., Magnussen, J., Engstad, T., & Indredavik, B. (2012). A long-term follow-up programme for maintenance of motor function after stroke: Protocol of the life after stroke—The last study. Stroke Research and Treatment. https://doi.org/10.1155/2012/392101 | No PAP-concept |
|  | Atkins CJ, Kaplan RM, Timms RM, Reinsch S, Lofback K. Behavioral exercise programs in the management of chronic obstructive pulmonary disease. J Consult Clin Psychol. 1984 Aug;52(4):591-603. doi: 10.1037//0022-006x.52.4.591. PMID: 6470285. | TIDier (insufficient reporting) |
|  | Baadjou, V. A. E., Verbunt, J. A. M. C. F., van Eijsden-Besseling, M. D. F., de Bie, R. A., Girard, O., Twisk, J. W. R., & Smeets V. A. E.; ORCID: https://orcid.org/0000-0002-7864-1938, R. J. E. M. A. I.-B. (2018). Preventing musculoskeletal complaints in music students: A randomized controlled trial. Occupational Medicine, 68(7), 469–477. https://dx.doi.org/10.1093/occmed/kqy105 | No PAP-concept |
|  | Bach, E., Beissner, K., Murtaugh, C., Trachtenberg, M., & Reid, M. C. (2013). Implementing a cognitive-behavioral pain self-management program in home health care, part 2: Feasibility and acceptability cohort study. Journal of Geriatric Physical Therapy (2001), 36(3), 130–137. https://doi.org/10.1519/JPT.0b013e31826ef84d | No PAP-concept |
|  | Bajwa, R. K., Goldberg, S. E., Van der Wardt, V., Burgon, C., Di Lorito, C., Godfrey, M., Dunlop, M., Logan, P., Masud, T., Gladman, J., Smith, H., Hood-Moore, V., Booth, V., Das Nair, R., Pollock, K., Vedhara, K., Edwards, R. T., Jones, C., Hoare, Z., Brand, A., … Harwood, R. H. (2019). A randomised controlled trial of an exercise intervention promoting activity, independence and stability in older adults with mild cognitive impairment and early dementia (PrAISED) - A Protocol. Trials, 20(1), 815. https://doi.org/10.1186/s13063-019-3871-9 | Wrong setting |
|  | Baldus, A., Bruggemann, S., Geidl, W., Gohner, W., Heimsoth, J., Huber, G., Kinkel, S., Klassen, O., Messner, T., Pfeifer, K., Probst, A., Rauch, A., Schulte, R., Schupp, W., Sudeck, G., & Bewegungstherapie, D.-A. (2021). Exercise Therapy and Exercise Demands in the Rehabilitation—Tasks and Goals for Research and Development DGRW Working Group on Exercise Therapy. REHABILITATION, 60(2), 152–158. | No PAP-concept |
|  | Bandura A. (1977). Self-efficacy: toward a unifying theory of behavioral change. Psychological review, 84(2), 191–215. https://doi.org/10.1037//0033-295x.84.2.191 | No PAP-concept |
|  | Barrett, E. M., Hussey, J. & Darker, C. D. (2017). Feasibility of a physical activity pathway for Irish primary care physiotherapy services. Physiotherapy, 103(1), 106–112. https://doi.org/10.1016/j.physio.2016.02.001 | Concept already included |
|  | Basler, H. D., Bertalanffy, H., Quint, S., Wilke, A., & Wolf, U. (2007). TTM-based counselling in physiotherapy does not contribute to an increase of adherence to activity recommendations in older adults with chronic low back pain—A randomised controlled trial. EUROPEAN JOURNAL OF PAIN, 11(1), 31–37. https://doi.org/10.1016/j.ejpain.2005.12.009 | Focus mainly on pain and not on PAP |
|  | Bassett, S. F., & Prapavessis, H. (2011). A test of an adherence-enhancing adjunct to physiotherapy steeped in the protection motivation theory. Physiotherapy Theory and Practice, 27(5), 360–372. https://doi.org/10.3109/09593985.2010.507238 | No PAP-concept |
|  | Bearne, L. M., Manning, V. L., Choy, E., Scott, D. L., & Hurley, M. V. (2017). Participants’ experiences of an Education, self-management and upper extremity eXercise Training for people with Rheumatoid Arthritis programme (EXTRA). PHYSIOTHERAPY, 103(4), 430–438. https://doi.org/10.1016/j.physio.2016.12.002 | Focus mainly on disease and not on PAP |
|  | Bearne, L. M., Volkmer, B., Peacock, J., Sekhon, M., Fisher, G., Holmes, M. N. G., Douiri, A., Amirova, A., Farran, D., Quirke-McFarlane, S., Modarai, B., Sackley, C., Weinman, J., Bieles, J., French, D., Williamson, E., Lowe, C. M., Longford, N., Patel, S., . . . May, P. (2022). Effect of a Home-Based, Walking Exercise Behavior Change Intervention vs Usual Care on Walking in Adults With Peripheral Artery Disease. *JAMA*, *327*(14),1344. https://doi.org/10.1001/jama.2022.3391 | No PAP-concept |
|  | Bearne, L., Galea Holmes, M., Bieles, J., Eddy, S., Fisher, G., Modarai, B., Patel, S., Peacock, J. L., Sackley, C., Volkmer, B., & Weinman, J. (2019). Motivating Structured walking Activity in people with Intermittent Claudication (MOSAIC): protocol for a randomised controlled trial of a physiotherapist-led, behavioural change intervention versus usual care in adults with intermittent claudication. BMJ open, 9(8), e030002. https://doi.org/10.1136/bmjopen-2019-030002 | Focus mainly on disease and not on PAP |
|  | Beissner K, Bach E, Murtaugh C, Parker SJ, Trachtenberg M, Reid MC. Implementing a cognitive-behavioral pain self-management program in home health care, part 1: program adaptation. J Geriatr Phys Ther. 2013 Jul-Sep;36(3):123-9. doi: 10.1519/JPT.0b013e31826ef67b. PMID: 22976814; PMCID: PMC4098704. | Focus mainly on pain and not on PAP |
|  | Bell, E. C., O’Halloran, P., Wallis, J. A., Crossley, K. M., Gibbs, A. J., Lee, A., Jennings, S. & Barton, C. J. (2023). Using SUpported Motivational InTerviewing (SUMIT) to increase physical activity for people with knee osteoarthritis: a pilot, feasibility randomised controlled trial. *BMJ Open*, *13*(11), e075014. https://doi.org/10.1136/bmjopen-2023-075014 | Exclusively e-health intervention |
|  | Ben-Ami, N., Chodick, G., Mirovsky, Y., Pincus, T., & Shapiro, Y. (2017). Increasing Recreational Physical Activity in Patients With Chronic Low Back Pain: A Pragmatic Controlled Clinical Trial. JOURNAL OF ORTHOPAEDIC & SPORTS PHYSICAL THERAPY, 47(2), 57–66. https://doi.org/10.2519/jospt.2017.7057 | Focus mainly on pain and not on PAP |
|  | Beneciuk, J. M., Robinson, M. E., & George, S. Z. (2012). Low back pain subgroups using fear-avoidance model measures: Results of a cluster analysis. The Clinical Journal of Pain, 28(8), 658–666. https://dx.doi.org/10.1097/AJP.0b013e31824306ed | No PAP-concept |
|  | Benedetti, Tânia Rosane Bertoldo; Schwingel, Andiara; Gomez, Luiz Salomão Ribas; Chodzko-Zajko, Wojtek . (2012). Programa “VAMOS” (Vida Ativa Melhorando a Saúde): da concepção aos primeiros resultados.. Revista Brasileira de Cineantropometria e Desempenho Humano, 14(6), –. doi:10.5007/1980-0037.2012v14n6p723 | Wrong setting |
|  | Berendsen, B. A. J., Hendriks, M. R. C., Rutten, G. M., Kremers, S. P. J., Savelberg, H., & Schaper, N. C. (2020). The added value of frequent physical activity group sessions in a combined lifestyle intervention: A cluster randomised trial in primary care. PREVENTIVE MEDICINE REPORTS, 20. https://doi.org/10.1016/j.pmedr.2020.101204 | No PAP-concept |
|  | Berg, S. K., Svendsen, J. H., Zwisler, A.-D., Pedersen, B. D., Preisler, P., Siersbæk-Hansen, L., Hansen, M. B., Nielsen, R. H., & Pedersen, P. U. (2011). COPE-ICD: a randomised clinical trial studying the effects and meaning of a comprehensive rehabilitation programme for ICD recipients -design, intervention and population. BMC Cardiovascular Disorders, 11, 33–33. https://doi.org/10.1186/1471-2261-11-33 | No PAP-concept |
|  | Bergström, C., Jensen, I., Hagberg, J., Busch, H., & Bergström, G. (2012). Effectiveness of different interventions using a psychosocial subgroup assignment in chronic neck and back pain patients: A 10-year follow-up. Disability and Rehabilitation, 34(2), 110–118. https://doi.org/10.3109/09638288.2011.607218 | No PAP-concept |
|  | Berner, P., Bezner, J. R., Morris, D., & Lein, D. H. (2021). Nutrition in Physical Therapist Practice: Tools and Strategies to Act Now. Physical Therapy, 101(5). https://doi.org/10.1093/ptj/pzab061 | No PAP-concept |
|  | Berra, K., Rippe, J., & Manson, J. E. (2015). Making Physical Activity Counseling a Priority in Clinical Practice: The Time for Action Is Now. JAMA: Journal of the American Medical Association, 314(24), 2617–2618. https://dx.doi.org/10.1001/jama.2015.16244 | No PAP-concept |
|  | Berry, M. J., Rejeski, W. J., Miller, M. E., Adair, N. E., Lang, W., Foy, C. G., & Katula, J. A. (2010). A lifestyle activity intervention in patients with chronic obstructive pulmonary disease. RESPIRATORY MEDICINE, 104(6), 829–839. https://doi.org/10.1016/j.rmed.2010.02.015 | TIDier (insufficient reporting) |
|  | Betschart, M., Rezek, S., Unger, I., Beyer, S., Gisi, D., Shannon, H., & Sieber, C. (2021). Feasibility of an Outpatient Training Program after COVID-19. INTERNATIONAL JOURNAL OF ENVIRONMENTAL RESEARCH AND PUBLIC HEALTH, 18(8). https://doi.org/10.3390/ijerph18083978 | No PAP-concept |
|  | Birch, S., Stilling, M., Mechlenburg, I., & Hansen, T. B. (2017). Effectiveness of a physiotherapist delivered cognitive-behavioral patient education for patients who undergoes operation for total knee arthroplasty: A protocol of a randomized controlled trial. BMC Musculoskeletal Disorders, 18(1). https://doi.org/10.1186/s12891-017-1476-6 | No PAP-concept |
|  | Blair, S. N., Sallis, R. E., Hutber, A., & Archer, E. (2012). Exercise therapy—The public health message. SCANDINAVIAN JOURNAL OF MEDICINE & SCIENCE IN SPORTS, 22(4), E24–E28. https://doi.org/10.1111/j.1600-0838.2012.01462.x | No PAP-concept |
|  | Bland, M. D., Birkenmeier, R. L., Barco, P., Lenard, E., Lang, C. E., & Lenze, E. J. (2016). Enhanced Medical Rehabilitation: Effectiveness of a clinical training model. | No PAP-concept |
|  | Blánquez Moreno, C., Colungo Francia, C., Alvira Balada, M. C., Kostov, B., González-de Paz, L., & Sisó-Almirall, A. (2018). [Effectiveness of an educational program for respiratory rehabilitation of Chronic Obstructive Pulmonary Disease patients in Primary Care in improving the quality of life, symptoms, and clinical risk]. Atencion primaria, 50(9), 539–546. https://doi.org/10.1016/j.aprim.2017.03.019 | Wrong language |
|  | Booth, V., Harwood, R. H., Hood-Moore, V., Bramley, T., Hancox, J. E., Robertson, K., Hall, J., Van Der Wardt, V., & Logan, P. A. (2018). Promoting activity, independence and stability in early dementia and mild cognitive impairment (PrAISED): development of an intervention for people with mild cognitive impairment and dementia. Clinical rehabilitation, 32(7), 855–864. https://doi.org/10.1177/0269215518758149 | Wrong setting |
|  | Brauer, S. G., Kuys, S. S., Paratz, J. D., & Ada, L. (2018). Improving physical activity after stroke via treadmill training and self management (IMPACT): A protocol for a randomised controlled trial. BMC Neurology, 18(1), 13–13. https://doi.org/10.1186/s12883-018-1015-6 | TIDier (insufficient reporting) |
|  | Brawley, L. R., Rejeski, W. J., & Lutes, L. (2000). A Group‐Mediated Cognitive‐Behavioral intervention for Increasing Adherence to Physical Activity in Older Adults. Journal of Applied Biobehavioral Research, 5(1), 47–65. https://doi.org/10.1111/j.1751-9861.2000.tb00063.x | No PAP-concept |
|  | Brawley, L. R., Rejeski, W. J., & Lutes, L. (2000). A Group‐Mediated Cognitive‐Behavioral intervention for Increasing Adherence to Physical Activity in Older Adults. Journal of Applied Biobehavioral Research, 5(1), 47–65. https://doi.org/10.1111/j.1751-9861.2000.tb00063.x | Concept already included |
|  | Briguglio, M., Cordani, C., Langella, F., Perazzo, P., Pregliasco, F., Banfi, G. & Wainwright, T. (2023). Why Treat Patients with a Major Orthopaedic Surgery Only to Send Them Back to the Vulnerable Conditions That Made Them Sick in the First Place? A Conceptual Scenario to Improve Patient’s Journey. *International Journal Of General Medicine*, *Volume 16*, 4729–4735. https://doi.org/10.2147/ijgm.s431055 | No PAP-concept |
|  | Brodin, N., Eurenius, E., Jensen, I., Nisell, R., Opava, C. H., & Grp, P. S. (2008). Coaching patients with early rheumatoid arthritis to healthy physical activity: A multicenter, randomized, controlled study. ARTHRITIS & RHEUMATISM-ARTHRITIS CARE & RESEARCH, 59(3), 325–331. https://doi.org/10.1002/art.23327 | No PAP-concept |
|  | Brosseau, L. (2011). Ottawa panel evidence-based clinical practice guidelines for patient education programmes in the management of osteoarthritis. Health Education Journal, 70(3), 318–358. https://doi.org/10.1177/0017896910394335 | No PAP-concept |
|  | Bruce, J., Mazuquin, B., Mistry, P., Rees, S., Canaway, A., Hossain, A., Williamson, E., Padfield, E. J., Lall, R., Richmond, H., Chowdhury, L., Lait, C., Petrou, S., Booth, K., Lamb, S. E., Vidya, R., & Thompson, A. M. (2022). Exercise to prevent shoulder problems after breast cancer surgery: The PROSPER RCT. Health Technology Assessment, 26(15). https://doi.org/10.3310/JKNZ2003 | Concept already included |
|  | Bury, T., & Moffat, M. (2014). Physiotherapists have a vital part to play in combatting the burden of noncommunicable diseases. Physiotherapy (United Kingdom), 100(2), 94–96. https://doi.org/10.1016/j.physio.2014.03.004 | Exclusively e-health intervention |
|  | Calbimonte, J.-P., Calvaresi, D., & Schumacher, M. (2021). Towards Collaborative Creativity in Persuasive Multi-agent Systems. Lecture Notes in Computer Science (including subseries Lecture Notes in Artificial Intelligence and Lecture Notes in Bioinformatics), 12946, 40–51. https://doi.org/10.1007/978-3-030-85739-4_4 | No PAP-concept |
|  | Calugi, S., Taricco, M., Rucci, P., Fugazzaro, S., Stuart, M., Dallolio, L., Pillastrini, P., & Fantini, M. P. (2016). Effectiveness of adaptive physical activity combined with therapeutic patient education in stroke survivors at twelve months: A non-randomized parallel group study. European Journal of Physical and Rehabilitation Medicine, 52(1), 72–80. | No PAP-concept |
|  | Canaway, A., Pincus, T., Underwood, M., Shapiro, Y., Chodick, G., & Ben-Ami, N. (2018). Is an enhanced behaviour change intervention cost-effective compared with physiotherapy for patients with chronic low back pain? Results from a multicentre trial in Israel. BMJ OPEN, 8(4). https://doi.org/10.1136/bmjopen-2017-019928 | Focus mainly on pain and not on PAP |
|  | Cederbom, S., Leveille, S. G., & Bergland, A. (2019). Effects of a behavioral medicine intervention on pain, health, and behavior among communitydwelling older adults: A randomized controlled trial. CLINICAL INTERVENTIONS IN AGING, 14, 1207–1220. https://doi.org/10.2147/CIA.S208102 | Wrong setting |
|  | Cederbom, S., Rydwik, E., Soderlund, A., Denison, E., Frandin, K., & Wagert, P. V. (2014). A behavioral medicine intervention for older women living alone with chronic pain—A feasibility study. CLINICAL INTERVENTIONS IN AGING, 9, 1383–1397. https://doi.org/10.2147/CIA.S66943 | Wrong setting |
|  | Chaléat-Valayer, E., Denis, A., Abelin-Genevois, K., Zelmar, A., Siani-Trebern, F., Touzet, S., Bergeret, A., Colin, C., & Fassier, J.-B. (2016). Long-term effectiveness of an educational and physical intervention for preventing low-back pain recurrence: A randomized controlled trial. Scandinavian Journal of Work, Environment and Health, 42(6), 510–519. https://doi.org/10.5271/sjweh.3597 | No PAP-concept |
|  | Champagne, E. R. (2023). Caregiver Resilience and Dance/Movement Therapy: A Theoretical Review and Conceptual Model. *Journal Of Applied Gerontology*, *43*(3), 319–327. https://doi.org/10.1177/07334648231210679 | No PAP-concept |
|  | Chang, L. P. (2013). Development of motivation to exercise in patients with Parkinson’s disease: An application of self determination theory. Dissertation Abstracts International: Section B: The Sciences and Engineering, 73(9), No-Specified. | No PAP-concept |
|  | Chevalley, T., Hoffmeyer, P., Bonjour, J.-P., & Rizzoli, R. (2002). An osteoporosis clinical pathway for the medical management of patients with low-trauma fracture. Osteoporosis International : A Journal Established as Result of Cooperation between the European Foundation for Osteoporosis and the National Osteoporosis Foundation of the USA, 13(6), 450–455. https://doi.org/10.1007/s001980200053 | No PAP-concept |
|  | Chevan J, Barrett M, Nowakowski K, Pappas K, Murphy H, Erck E, Weisner S. Responding to stakeholder needs to engage rehabilitation professionals in the delivery of evidence-based health programming for adults with osteoarthritis. Front Rehabil Sci. 2022 Aug 2;3:907477. doi: 10.3389/fresc.2022.907477. PMID: 36188917; PMCID: PMC9397695. | No PAP-concept |
|  | Cinciripini, P. M., & Floreen, A. (1982). An evaluation of a behavioral program for chronic pain. Journal of Behavioral Medicine, 5(3), 375–389. https://doi.org/10.1007/BF00846164 | Focus mainly on pain and not on PAP |
|  | Coca-Martinez, M., Lopez-Hernandez, A., Montane-Muntane, M., Arguis, M. J., Gimeno-Santos, E., Navarro-Ripoll, R., Perdomo, J., Lopez-Baamonde, M., Rios, J., Moises, J., de la Garza, M. S., Sandoval, E., Romano, B., Sebio, R., Dana, F., & Martinez-Palli, G. (2020). Multimodal prehabilitation as strategy for reduction of postoperative complications after cardiac surgery: A randomised controlled trial protocol. BMJ OPEN, 10(12). https://doi.org/10.1136/bmjopen-2020-039885 | No PAP-concept |
|  | Courtney, M. D., Edwards, H. E., Chang, A. M., Parker, A. W., Finlayson, K., Bradbury, C., & Nielsen Helen E.; ORCID: https://orcid.org/0000-0002-4451-0577 AI - Finlayson, Z. A. I.-E., Kathleen; ORCID: https://orcid. org/0000-0002-5743-2731. (2012). Improved functional ability and independence in activities of daily living for older adults at high risk of hospital readmission: A randomized controlled trial. Journal of Evaluation in Clinical Practice, 18(1), 128–134. https://dx.doi.org/10.1111/j.1365-2753.2010.01547.x | No PAP-concept |
|  | Courtney, M., Edwards, H., Chang, A., Parker, A., Finlayson, K., & Hamilton, K. (2009). Fewer emergency readmissions and better quality of life for older adults at risk of hospital readmission: a randomized controlled trial to determine the effectiveness of a 24-week exercise and telephone follow-up program. Journal of the American Geriatrics Society, 57(3), 395–402. https://doi.org/10.1111/j.1532-5415.2009.02138.x | No PAP-concept |
|  | Cowley, A., Booth, V., Di Lorito, C., Chandria, P., Chadwick, O., Stanislas, C., Dunlop, M., Howe, L., Harwood, R. H., & Logan, P. A. (2022). A Qualitative Study on the Experiences of Therapists Delivering the Promoting Activity, Independence and Stability in Early Dementia (PrAISED) Intervention During the COVID-19 Pandemic. Journal of Alzheimer’s Disease : JAD. https://doi.org/10.3233/JAD-220424 | No PAP-concept |
|  | Creamer, P., Singh, B. B., Hochberg, M. C., & Berman, B. M. (2000). Sustained improvement produced by nonpharmacologic intervention in fibromyalgia: Results of a pilot study. ARTHRITIS CARE AND RESEARCH, 13(4), 198–204. https://doi.org/10.1002/1529-0131(200008)13:4<198::AID-ANR4>3.0.CO;2-P | No PAP-concept |
|  | Crozier, A (2023) Exploration of embedding registered Clinical Exercise Physiologists into clinical exercise service provision in the UK. Doctoral thesis, Liverpool John Moores University.10.24377/LJMU.t.00019191 | No PAP-concept |
|  | Da Vico, L., Ciompi, M., Schininà, F., Sogaro, E., Mannelli, W., & Cortini, S. (2014). [Multidisciplinary team in cardiac rehabilitation and secondary prevention, from the assessment to the education: An educational project]. Monaldi archives for chest disease = Archivio Monaldi per le malattie del torace, 82(1), 35–42. https://doi.org/10.4081/monaldi.2014.41 | Wrong language |
|  | Danzl, M. M., Harrison, A., Hunter, E. G., Kuperstein, J., Sylvia, V., Maddy, K., & Campbell, S. (2016). „A Lot of Things Passed Me by“: Rural Stroke Survivors’ and Caregivers’ Experience of Receiving Education From Health Care Providers. JOURNAL OF RURAL HEALTH, 32(1), 13–24. https://doi.org/10.1111/jrh.12124 | No PAP-concept |
|  | Dapp, U., Anders, J., Von Renteln-Kruse, W., & Meier-Baumgartner, H. P. (2005). Active health promotion in old age: Methodology of a preventive intervention programme provided by an interdisciplinary health advisory team for independent older people. Journal of Public Health, 13(3), 122–127. https://doi.org/10.1007/s10389-004-0097-3 | Wrong setting |
|  | Daun, J. T., Capozzi, L. C., Roldan Urgoiti, G., McDonough, M. H., Easaw, J. C., McNeely, M. L., Francis, G. J., Williamson, T., Danyluk, J., McLaughlin, E., Ospina, P. A., de Guzman Wilding, M., Radke, L., Driga, A., Lesiuk, C., & Culos-Reed, S. N. (2022). ACE-Neuro: A tailored exercise oncology program for neuro-oncology patients – Study protocol. Contemporary Clinical Trials Communications, 28. https://doi.org/10.1016/j.conctc.2022.100925 | Exclusively e-health intervention |
|  | Davenport, T. E., Stevens, S. R., VanNess, M. J., Snell, C. R., & Little, T. (2010). Conceptual model for physical therapist management of chronic fatigue syndrome/myalgic encephalomyelitis. Physical Therapy, 90(4), 602–614. https://doi.org/10.2522/ptj.20090047 | No PAP-concept |
|  | Davidson, D. M., & Maloney, C. A. (1985). Recovery after cardiac events. Physical Therapy, 65(12), 1820–1827. https://doi.org/10.1093/ptj/65.12.1820 | Focus mainly on disease and not on PAP |
|  | de Dios-Rodríguez E, Patino-Alonso C, González-Sánchez S, Tamayo-Morales O, Ripoll J, Mora-Simón S, Unzueta-Arce J, Gómez-Marcos MA, García-Ortiz L, Rodríguez-Sánchez E. Promoting Physical Activity in a Primary Care Practice in People Living with Dementia and Their Family Caregivers. Healthcare (Basel). 2023 Apr 27;11(9):1255. doi: 10.3390/healthcare11091255. PMID: 37174797; PMCID: PMC10178700 | Wrong setting |
|  | De Janssen, V., Gucht, V., van Exel, H., & Maes, S. (2014). A self-regulation lifestyle program for post-cardiac rehabilitation patients has long-term effects on exercise adherence. Journal of Behavioral Medicine, 37(2), 308–321. https://dx.doi.org/10.1007/s10865-012-9489-y | TIDier (insufficient reporting) |
|  | De Leeuwerk, M., De Groot, V., Dam, S. T., Kruizenga, H., Weijs, P., Geleijn, E., Van der Leeden, M., Van der Schaaf, M., Dickhoff, C., Besselink, M. G., Tuynman, J. B., Van Berge Henegouwen, M. I., Erdmann, J. I., Huijsmans, R. J., Van der Ploeg, H. P., Eskes, A. M., Pijnappels, M. A. G. M., Van Leeuwen, L. S., Smits, A. B., . . . Grimbergen, E. (2023). The efficacy of a blended intervention to improve physical activity and protein intake for optimal physical recovery after oncological gastrointestinal and lung cancer surgery, the Optimal Physical Recovery After Hospitalization (OPRAH) trial: study protocol for a randomized controlled multicenter trial. *Trials*, *24*(1). https://doi.org/10.1186/s13063-023-07705-2 | Exclusively e-health intervention |
|  | de Melo Ghisi, G. L., Grace, S. L., Thomas, S., & Oh Sherry L.; ORCID: https://orcid.org/0000-0001-7063-3610 AI - Oh, P. A. I.-G., Paul; ORCID: https://orcid. org/0000-0002-0603-6958. (2015). Behavior determinants among cardiac rehabilitation patients receiving educational interventions: An application of the health action process approach. Patient Education and Counseling, 98(5), 612–621. https://dx.doi.org/10.1016/j.pec.2015.01.006 | TIDier (insufficient reporting) |
|  | de Vos, B. C., Runhaar, J., Van Middelkoop, M., Krul, M., & Bierma-Zeinstra, S. M. A. (2016). Long-term effects of a randomized, controlled, tailor-made weight-loss intervention in primary care on the health and lifestyle of overweight and obese women. American Journal of Clinical Nutrition, 104(1), 33–40. https://doi.org/10.3945/ajcn.116.133512 | Focus mainly on disease and not on PAP |
|  | de Vos, B.C., Runhaar, J. & Bierma-Zeinstra, S.M.A. Effectiveness of a tailor-made weight loss intervention in primary care. European Journal of Nutrition 53, 95–104 (2014). https://doi.org/10.1007/s00394-013-0505-y | Wrong setting |
|  | de Vries, N. M., van Ravensberg, C. D., Hobbelen, J. S. M., van der Wees, P. J., Rikkert, M., Staal, J. B., & Nijhuis-van der Sanden, M. W. G. (2015). The Coach2Move Approach: Development and Acceptability of an Individually Tailored Physical Therapy Strategy to Increase Activity Levels in Older Adults With Mobility Problems. JOURNAL OF GERIATRIC PHYSICAL THERAPY, 38(4), 169–182. https://doi.org/10.1519/JPT.0000000000000038 | Concept already included |
|  | Dean, E. (2009a). Physical therapy in the 21st century (Part I): Toward practice informed by epidemiology and the crisis of lifestyle conditions. Physiotherapy Theory and Practice, 25(5), 330–353. https://doi.org/10.1080/09593980802668027 | No PAP-concept |
|  | Dean, E. (2009b). Physical therapy in the 21st century (Part II): Evidence-based practice within the context of evidence-informed practice. Physiotherapy Theory and Practice, 25(5), 354–368. https://doi.org/10.1080/09593980902813416 | No PAP-concept |
|  | Dean, E., Al-Obaidi, S., De Andrade, A. D., Gosselink, R., Umerah, G., Al-Abdelwahab, S., Anthony, J., Bhise, A. R., Bruno, S., Butcher, S., Olsén, M. F., Frownfelter, D., Gappmaier, E., Gylfadttir, S., Habibi, M., Hanekom, S., Hasson, S., Jones, A., Lapier, T., … Wong, A. W. P. (2011). The First Physical Therapy Summit on Global Health: Implications and Recommendations for the 21st century. Physiotherapy Theory and Practice, 27(8), 531–547. https://doi.org/10.3109/09593985.2010.544052 | No PAP-concept |
|  | Dean, E., Dornelas De Andrade, A., O’Donoghue, G., Skinner, M., Umereh, G., Beenen, P., Cleaver, S., Afzalzada, D., Fran Delaune, M., Footer, C., Gannotti, M., Gappmaier, E., Figl-Hertlein, A., Henderson, B., Hudson, M. K., Spiteri, K., King, J., Klug, J. L., Laakso, E.-L., … Wong, W. P. (2014). The second physical therapy summit on global health: Developing an action plan to promote health in daily practice and reduce the burden of non-communicable diseases. Physiotherapy Theory and Practice, 30(4), 261–275. https://doi.org/10.3109/09593985.2013.856977 | No PAP-concept |
|  | Dean, E., Skinner, M., Myezwa, H., Mkumbuzi, V., Mostert, K., Parra, D. C., Shirley, D., Söderlund, A., De Andrade, A. D., Abaraogu, U. O., Bruno, S., Clark, D., Gylfadóttir, S., Jones, A., Veluswamy, S. K., Lomi, C., Moffat, M., Morris, D., Stensdotter, A.-K., & Wong, W. P. (2019). Health Competency Standards in Physical Therapist Practice. Physical Therapy, 99(9), 1242–1254. https://doi.org/10.1093/ptj/pzz087 | No PAP-concept |
|  | Deguchi, N., Izawa, S., Hirakawa, Y., & Higaki, Y. (2017). Effects of pre-exercise patient education for promoting physical activity among knee pain patients visiting a clinic: A pilot study. Rigakuryoho Kagaku, 32(6), 861–867. https://doi.org/10.1589/rika.32.861 | Wrong language |
|  | Denis, A., Zelmar, A., Le Pogam, M.-A., Chaleat-Valayer, E., Bergeret, A., & Colin, C. (2012). The PRESLO study: Evaluation of a global secondary low back pain prevention program for health care personnel in a hospital setting. Multicenter, randomized intervention trial. BMC Musculoskeletal Disorders, 13, 234–234. https://doi.org/10.1186/1471-2474-13-234 | No PAP-concept |
|  | Dennett, A. M., Shields, N., Peiris, C. L., Prendergast, L. A., O’Halloran, P. D., Parente, P., & Taylor, N. F. (2018). Motivational interviewing added to oncology rehabilitation did not improve moderate-intensity physical activity in cancer survivors: A randomised trial. JOURNAL OF PHYSIOTHERAPY, 64(4), 255–263. https://doi.org/10.1016/j.jphys.2018.08.003 | Focus mainly on disease and not on PAP |
|  | Dias, K. J., Shoemaker, M. J., Lefebvre, K. M., & Heick, J. D. (2021). A Knowledge Translation Framework for Optimizing Physical Therapy in Patients With Heart Failure. Physical Therapy, 101(6). https://doi.org/10.1093/ptj/pzab079 | Focus mainly on disease and not on PAP |
|  | Driver, S., Rees, K., O’Connor, J., & Lox Simon; ORCID: https://orcid.org/0000-0003-2356-432X, C. A. I.-D. (2006). Aquatics, health-promoting self-care behaviours and adults with brain injuries. Brain Injury, 20(2), 133–141. https://dx.doi.org/10.1080/02699050500443822 | No PAP-concept |
|  | Dufour, S. P., Graham, S., Friesen, J., Rosenblat, M., Rous, C., & Richardson, J. (2015). Physiotherapists supporting self-management through health coaching: A mixed methods program evaluation. PHYSIOTHERAPY THEORY AND PRACTICE, 31(1), 29–38. https://doi.org/10.3109/09593985.2014.930769 | TIDier (insufficient reporting) |
|  | Duijzer, G., Jansen, S. C., Haveman-Nies, A., van Bruggen, R., ter Beek, J., Hiddink, G. J., & Feskens, E. J. M. (2012). Translating the SLIM diabetes prevention intervention into SLIMMER: Implications for the dutch primary health care. Family Practice, 29, i145–i152. https://doi.org/10.1093/fampra/cmr096 | TIDier (insufficient reporting) |
|  | Duong, V., Dennis, S., Ferreira, M. L., Nicolson, P., O’Connell, R., Robbins, S. R., Wang, X., & Hunter, D. J. (2022). Correlations between objective and self-reported step count adherence following total knee replacement: A longitudinal repeated-measures cohort study. Physiotherapy Research International. https://doi.org/10.1002/pri.1966 | Exclusively e-health intervention |
|  | Effering, T., Zielhuis, G., Kerstjens, H., van der Valk, P., & van der Palen, J. (2011). Community based physiotherapeutic exercise in COPD self-management: a randomised controlled trial. Respiratory medicine, 105(3), 418–426. https://doi.org/10.1016/j.rmed.2010.09.017 | No PAP-concept |
|  | Eisele, A., Schagg, D., Kramer, L. V., Kuffner, R., Reusch, A., & Gohner, W. (2022). Supporting patient adherence to physical activity and exercise: Evaluation of a behavior change counseling training program for physiotherapists. PHYSIOTHERAPY THEORY AND PRACTICE, 38(10), 1426–1437. https://doi.org/10.1080/09593985.2021.1872128 | No PAP-concept |
|  | Ellis, T., & Motl, R. W. (2013). Physical Activity Behavior Change in Persons With Neurologic Disorders: Overview and Examples From Parkinson Disease and Multiple Sclerosis. JOURNAL OF NEUROLOGIC PHYSICAL THERAPY, 37(2), 85–90. https://doi.org/10.1097/NPT.0b013e31829157c0 | No PAP-concept |
|  | Elvén, M., Hochwälder, J., Dean, E., & Söderlund, A. (2018). Development and initial evaluation of an instrument to assess physiotherapists’ clinical reasoning focused on clients’ behavior change. Physiotherapy Theory and Practice, 34(5), 367–383. https://doi.org/10.1080/09593985.2017.1419521 | Concept already included |
|  | Ennis, K., Hawthorne, K., & Frownfelter, D. (2012). How Physical Therapists Can Strategically Effect Health Outcomes for Older Adults With Limited Health Literacy. JOURNAL OF GERIATRIC PHYSICAL THERAPY, 35(3), 148–154. https://doi.org/10.1519/JPT.0b013e31823ae6d1 | No PAP-concept |
|  | Epstein, L. H. (1998). Integrating theoretical approaches to promote physical therapy. American Journal of Preventive Medicine, 15(4), 257–265. https://dx.doi.org/10.1016/S0749-3797%2898%2900083-X | No PAP-concept |
|  | Eriksson, K. M., Westborg, C.-J., & Eliasson, M. C. E. (2006). A randomized trial of lifestyle intervention in primary healthcare for the modification of cardiovascular risk factors The Björknäs study. Scandinavian Journal of Public Health, 34(5), 453–461. https://doi.org/10.1080/14034940500489826 | No PAP-concept |
|  | Errickson, S. P., Kolotkin, R. L., Skidmore, M. S., Endress, G., Østbye, T., Crosby, R., & Eisenson, H. (2016). Improvements in Functional Exercise Capacity after a Residential Behavioural Change, Diet and Fitness Program for Obese Adults. Physiotherapy Research International, 21(2), 84–90. https://doi.org/10.1002/pri.1623 | No PAP-concept |
|  | Fasczewski, K. S., Garner, L. M., Clark, L. A., Michels, H. S., & Migliarese, S. J. (2022). Medical Therapeutic Yoga for multiple sclerosis: Examining self-efficacy for physical activity, motivation for physical activity, and quality of life outcomes. Disability and Rehabilitation: An International, Multidisciplinary Journal, 44(1), 106–113. https://dx.doi.org/10.1080/09638288.2020.1760364 | No PAP-concept |
|  | Fortier, M., Guérin, E., & Segar, M. L. (2016). Words matter: Reframing exercise is medicine for the general population to optimize motivation and create sustainable behaviour change. Applied Physiology, Nutrition, and Metabolism = Physiologie Appliquee, Nutrition et Metabolisme, 41(11), 1212–1215. https://doi.org/10.1139/apnm-2016-0125 | No PAP-concept |
|  | Fowler, B., Jamrozik, K., Norman, P., Allen, Y., & Wilkinson, E. (2002). Improving maximum walking distance in early peripheral arterial disease: Randomised controlled trial. The Australian Journal of Physiotherapy, 48(4), 269–275. https://doi.org/10.1016/s0004-9514(14)60166-5 | No PAP-concept |
|  | Fox, L., Wiseman, T., Cahill, D., Fleure, L., Kinsella, J., & Van Hemelrijck, M. (2018). Brief behavioural intervention, delivered as standard care, to support physical activity engagement in men with prostate cancer: A pilot study protocol. BMJ Open Sport and Exercise Medicine, 4(1). https://doi.org/10.1136/bmjsem-2018-000469 | TIDier (insufficient reporting) |
|  | Foy, C. G., Wickley, K. L., Adair, N. N., Lang, W., Miller, M. E., Rejeski, W. J., Woodard, C. M., & Berry, M. J. (2006). The Reconditioning Exercise and Chronic Obstructive Pulmonary Disease Trial II (REACT II): Rationale and study design for a clinical trial of physical activity among individuals with chronic obstructive pulmonary disease. CONTEMPORARY CLINICAL TRIALS, 27(2), 135–146. https://doi.org/10.1016/j.cct.2005.11.011 | TIDier (insufficient reporting) |
|  | Freene, N., Davey, R., & McPhail, S. M. (2019). Frequency of a very brief intervention by physiotherapists to increase physical activity levels in adults: Apilot randomised controlled trial. BMC SPORTS SCIENCE MEDICINE AND REHABILITATION, 11. https://doi.org/10.1186/s13102-019-0118-8 | TIDier (insufficient reporting) |
|  | Freene, N., Waddington, G., Chesworth, W., Davey, R., & Goss, J. (2011). „Physical activity at home (PAAH)“, evaluation of a group versus home based physical activity program in community dwelling middle aged adults: Rationale and study design. BMC PUBLIC HEALTH, 11. https://doi.org/10.1186/1471-2458-11-883 | No PAP-concept |
|  | Gallagher, K. M. (2016). Helping Older Adults Sustain Their Physical Therapy Gains: A Theory-Based Intervention to Promote Adherence to Home Exercise Following Rehabilitation. Journal of Geriatric Physical Therapy (2001), 39(1), 20–29. https://doi.org/10.1519/JPT.0000000000000040 | TIDier (insufficient reporting) |
|  | Garner, S., Fenton, T., Martin, L., Creaser, C., Johns, C., & Barnabe, C. (2018). Personalized diet and exercise recommendations in early rheumatoid arthritis: A feasibility trial. Musculoskeletal Care, 16(1), 167–172. https://doi.org/10.1002/msc.1214 | No PAP-concept |
|  | Geidl, W., Hofmann, J., Gohner, W., Sudeck, G., & Pfeifer, K. (2012). Behaviour-Orientated Exercise Therapy—Initiating and Maintaining a Physically Active Lifestyle. REHABILITATION, 51(4), 259–268. https://doi.org/10.1055/s-0031-1280803 | Concept already included |
|  | George, S. Z., Fritz, J. M., Bialosky, J. E., & Donald, D. A. (2003). The effect of a fear-avoidance-based physical therapy intervention for patients with acute low back pain: results of a randomized clinical trial. Spine, 28(23), 2551–2560. https://doi.org/10.1097/01.BRS.0000096677.84605.A2 | Focus mainly on pain and not on PAP |
|  | George, S. Z., Zeppieri, G. Jr., Cere, A. L., Cere, M. R., Borut, M. S., Hodges, M. J., Reed, D. M., Valencia, C., & Robinson, M. E. (2008). A randomized trial of behavioral physical therapy interventions for acute and sub-acute low back pain (NCT00373867). Pain, 140(1), 145–157. https://dx.doi.org/10.1016/j.pain.2008.07.029 | No PAP-concept |
|  | Gerage, A. M., Benedetti, T. R. B., Cavalcante, B. R., Farah, B. Q., & Ritti-Dias, R. M. (2020). Efficacy of a behavior change program on cardiovascular parameters in patients with hypertension: A randomized controlled trial. Einstein (Sao Paulo, Brazil), 18, eAO5227–eAO5227. https://doi.org/10.31744/einstein_journal/2020AO5227 | Focus mainly on disease and not on PAP |
|  | Gerage, A. M., Benedetti, T. R. B., Ritti-Dias, R. M., Dos Santos, A. C. O., de Souza, B. C. C., & Almeida, F. A. (2017). Effectiveness of a Behavior Change Program on Physical Activity and Eating Habits in Patients With Hypertension: A Randomized Controlled Trial. Journal of physical activity & health, 14(12), 943–952. https://doi.org/10.1123/jpah.2016-0268 | TIDier (insufficient reporting) |
|  | Gervais-Hupe, J., Filleul, A., Perreault, K., & Hudon, A. (o. J.). Implementation of a biopsychosocial approach into physiotherapists’ practice: A review of systematic reviews to map barriers and facilitators and identify specific behavior change techniques. DISABILITY AND REHABILITATION. https://doi.org/10.1080/09638288.2022.2094479 | No PAP-concept |
|  | Giangregorio, L. M., Thabane, L., Adachi, J. D., Ashe, M. C., Bleakney, R. R., Braun, E. A., Cheung, A. M., Fraser, L. A., Gibbs, J. C., Hill, K. D., Hodsman, A. B., Kendler, D. L., Mittmann, N., Prasad, S., Scherer, S. C., Wark, J. D., & Papaioannou, A. (2014). Build Better Bones With Exercise: Protocol for a Feasibility Study of a Multicenter Randomized Controlled Trial of 12 Months of Home Exercise in Women With a Vertebral Fracture. PHYSICAL THERAPY, 94(9), 1337–1352. https://doi.org/10.2522/ptj.20130625 | TIDier (insufficient reporting) |
|  | Gnagnarella P, Dragà D, Raja S, Baggi F, Simoncini MC, Sabbatini A, Mazzocco K, Masiero M, Bassi FD, Peradze N, Zorzino L, Latella M, Pravettoni G, Maisonneuve P. Physical activity and/or dietary intervention in overweight or obese breast cancer survivors: results of the InForma randomized trial. J Cancer Surviv. 2023 Jul 7. doi: 10.1007/s11764-023-01415-z. Epub ahead of print. PMID: 37418169. | TIDier (insufficient reporting) |
|  | Gnagnarella, P., Marvaso, G., Jereczek-Fossa, B. A., de Cobelli, O., Simoncini, M. C., Teixeira, L. F. N., Sabbatini, A., Pravettoni, G., Johansson, H., Nezi, L., Muto, P., Borzillo, V., Celentano, E., Crispo, A., Pinto, M., Cavalcanti, E., Gandini, S., & Grp, M. C. (2022). Life style and interaction with microbiota in prostate cancer patients undergoing radiotherapy: Study protocol for a randomized controlled trial. BMC CANCER, 22(1). https://doi.org/10.1186/s12885-022-09521-4 | No PAP-concept |
|  | Göhner, W., Schagg, D., Küffner, R., & Reusch, A. (2018). Psychologische Strategien zur Bewegungsförderung: Entwicklung von Fortbildungen für die Bewegungstherapie (BeFo). B&G Bewegungstherapie Und Gesundheitssport, 34(04), 168–177. https://doi.org/10.1055/a-0641-8776 | No PAP-concept |
|  | Goldberg, S. E., van der Wardt, V., Brand, A., Burgon, C., Bajwa, R., Hoare, Z., Logan, P. L., Harwood, R. H., Gladman, J., Edwards, R. T., Masud, T., Vedhara, K., Pollock, K., Booth, V., Das Nair, R., Kearney, F., Orrell, M., Hood, V., Robertson, K., … Grp, P. S. (2019). Promoting activity, Independence and stability in early dementia (PrAISED): A, multisite, randomised controlled, feasibility trial. BMC GERIATRICS, 19(1). https://doi.org/10.1186/s12877-019-1379-5 | TIDier (insufficient reporting) |
|  | Gunnes, M., Langhammer, B., Aamot, I. L., Lydersen, S., Ihle-Hansen, H., Indredavik, B., Reneflot, K. H., Schroeter, W., Askim, T., & Grp, L. C. (2019). Adherence to a Long-Term Physical Activity and Exercise Program After Stroke Applied in a Randomized Controlled Trial. PHYSICAL THERAPY, 99(1), 74–85. https://doi.org/10.1093/ptj/pzy126 | Concept already included |
|  | Haldorsen, E. M. H., Kronholm, K., Skouen, J. S., & Ursin, H. (1998). Predictors for outcome of a multi-modal cognitive behavioural treatment program for low back pain patients—A 12-month follow-up study. European Journal of Pain, 2(4), 293–307. https://doi.org/10.1016/S1090-3801(98)90028-3 | Focus mainly on pain and not on PAP |
|  | Hale, L. A., Mulligan, H. F., Treharne, G. J., & Smith, C. M. (2013). The feasibility and short-term benefits of Blue Prescription: a novel intervention to enable physical activity for people with multiple sclerosis. Disability and rehabilitation, 35(14), 1213–1220. https://doi.org/10.3109/09638288.2012.723787 | Wrong setting |
|  | Hale, L. A., Smith, C., Mulligan, H., & Treharne, G. J. (2012). „Tell me what you want, what you really really want….“: Asking people with multiple sclerosis about enhancing their participation in physical activity. Disability and Rehabilitation, 34(22), 1887–1893. https://doi.org/10.3109/09638288.2012.670037 | No PAP-concept |
|  | Harman, K., MacRae, M., & Vallis, M. (2014a). The Development and Testing of a Checklist to Study Behaviour Change Techniques used in a Treatment Programme for Canadian Armed Forces Members with Chronic Non-specific Low Back Pain. PHYSIOTHERAPY CANADA, 66(3), 313–321. https://doi.org/10.3138/ptc.2013-55BC | Focus mainly on pain and not on PAP |
|  | Harman, K., MacRae, M., Vallis, M., & Bassett, R. (2014b). Working with People to Make Changes: A Behavioural Change Approach Used in Chronic Low Back Pain Rehabilitation. PHYSIOTHERAPY CANADA, 66(1), 82–90. https://doi.org/10.3138/ptc.2012-56BC | Focus mainly on pain and not on PAP |
|  | Harrold, S. A., Libet, J., Pope, C., Lauerer, J. A., Johnson, E., & Edlund, B. J. (2018). Increasing physical activity for veterans in the Mental Health Intensive Case Management Program: A community-based intervention. PERSPECTIVES IN PSYCHIATRIC CARE, 54(2), 266–273. https://doi.org/10.1111/ppc.12233 | No PAP-concept |
|  | Hatlova, B., Chalupova, E., & Wedlichova, I. (2023). Long term effects of sport activation programme on mental health of psychiatric outpatients. Dlouhodobý vliv sportovního aktivačního programu na duševní zdraví ambulantních psychiatrických pacientů, 119(2), 53-61. | Wrong language |
|  | Haugmark, T., Hagen, K. B., Provan, S. A., Bærheim, E., & Zangi, H. A. (2018). Effects of a community-based multicomponent rehabilitation programme for patients with fibromyalgia: Protocol for a randomised controlled trial. BMJ Open, 8(6), e021004–e021004. https://doi.org/10.1136/bmjopen-2017-021004 | TIDier (insufficient reporting) |
|  | Hay-Smith, E. J. C., McClurg, D., Frawley, H., & Dean, S. G. (2016). Exercise adherence: Integrating theory, evidence and behaviour change techniques. Physiotherapy, 102(1), 7–9. https://doi.org/10.1016/j.physio.2015.08.006 | No PAP-concept |
|  | Heinrich, K. M., Crawford, D. A., Langford, C. R., Kehler, A., & Andrews, V. (2021). High-Intensity Functional Training Shows Promise for Improving Physical Functioning and Activity in Community-Dwelling Older Adults: A Pilot Study. JOURNAL OF GERIATRIC PHYSICAL THERAPY, 44(1), 9–17. https://doi.org/10.1519/JPT.0000000000000251 | No PAP-concept |
|  | Hesselink, A. E., Bilo, H. J. G., Jonkers, R., Martens, M., de Weerdt, I., & Rutten, G. E. H. (2013). A cluster-randomized controlled trial to study the effectiveness of a protocol-based lifestyle program to prevent type 2 diabetes in people with impaired fasting glucose. BMC FAMILY PRACTICE, 14. https://doi.org/10.1186/1471-2296-14-184 | TIDier (insufficient reporting) |
|  | Higgs, C., Skinner, M., & Hale, L. (2016). Outcomes of a community-based lifestyle programme for adults with diabetes or pre-diabetes. Journal of Primary Health Care, 8(2), 130–139. https://doi.org/10.1071/HC15038 | No PAP-concept |
|  | Hisham, H., Justine, M., Hasnan, N., & Manaf, H. (2022). Effects of Paraplegia Fitness Integrated Training on Physical Function and Exercise Self-Efficacy and Adherence Among Individuals With Spinal Cord Injury. Annals of Rehabilitation Medicine, 46(1), 33–44. https://doi.org/10.5535/arm.21127 | TIDier (insufficient reporting) |
|  | Ho, E. K., Ferreira, M. L., Bauman, A., Hodges, P. W., Maher, C. G., Simic, M., Morton, R. L., Lonsdale, C., Li, Q., Baysari, M. T., Amorim, A. B., Ceprnja, D., Clavisi, O., Halliday, M., Jennings, M., Kongsted, A., Maka, K., Reid, K., Reynolds, T., & Ferreira, P. H. (2021). Effectiveness of a coordinated support system linking public hospitals to a health coaching service compared with usual care at discharge for patients with chronic low back pain: Protocol for a randomised controlled trial. BMC MUSCULOSKELETAL DISORDERS, 22(1). https://doi.org/10.1186/s12891-021-04479-z | Exclusively e-health intervention |
|  | Hoekstra, F., van Offenbeek, M. A. G., Dekker, R., Hettinga, F. J., Hoekstra, T., van der Woude, L. H. V., van der Schans, C. P., & Grp, R. (2017). Implementation fidelity trajectories of a health promotion program in multidisciplinary settings: Managing tensions in rehabilitation care. IMPLEMENTATION SCIENCE, 12. https://doi.org/10.1186/s13012-017-0667-8 | No PAP-concept |
|  | Hoffmann, T., Bakhit, M., & Michaleff, Z. (2022). Shared decision making and physical therapy: What, when, how, and why? Brazilian Journal of Physical Therapy, 26(1), 100382–100382. https://doi.org/10.1016/j.bjpt.2021.100382 | No PAP-concept |
|  | Holland, S. K., Greenberg, J., Tidwell, L., Malone, J., Mullan, J., & Newcomer, R. (2005). Community-Based Health Coaching, Exercise, and Health Service Utilization. Journal of Aging and Health, 17(6), 697–716. https://dx.doi.org/10.1177/0898264305277959 | No PAP-concept |
|  | Horstmannshoff C, Skudlik S, Petermann J, Kiesel T, Döringer T, Crispin A, Hermsdörfer J, Köberlein-Neu J, Jahn K, Schädler S, Bauer P, Voigt K, Müller M. Effectiveness of an evidence-based care pathway to improve mobility and participation in older patients with vertigo and balance disorders in primary care (MobilE-PHY2): study protocol for a multicentre cluster-randomised controlled trial. Trials. 2023 Feb 6;24(1):91. doi: 10.1186/s13063-022-07017-x. PMID: 36747256; PMCID: PMC9902065. | Wrong setting |
|  | Hughes, S. L., Seymour, R. B., Campbell, R. T., Huber, G., Pollak, N., Sharma, L., & Desai, P. (2006). Long-term impact of Fit and Strong! on older adults with osteoarthritis. The Gerontologist, 46(6), 801–814. https://doi.org/10.1093/geront/46.6.801 | No PAP-concept |
|  | Hughes, S. L., Seymour, R. B., Campbell, R., Pollak, N., Huber, G., & Sharma, L. (2004). Impact of the fit and strong intervention on older adults with osteoarthritis. The Gerontologist, 44(2), 217–228. https://doi.org/10.1093/geront/44.2.217 | No PAP-concept |
|  | Hui, K. L., Hagger, M. S., Goh, V. H. H., Hart, W. G., Gucciardi, D. F., Koh, L. H., Hagger, M. S., Goh, V. H. H., Hart, W. G., & Gucciardi Daniel F.; ORCID: https://orcid.org/0000-0001-5448-3990, D. F. A. I.-G. (2017). Effects of a brief action and coping planning intervention on completion of preventive exercises prescribed by a physiotherapist among people with knee pain. JOURNAL OF SCIENCE AND MEDICINE IN SPORT, 20(8), 723–728. https://dx.doi.org/10.1016/j.jsams.2017.02.008 | TIDier (insufficient reporting) |
|  | Hurley, D. A., Murphy, L. C., Hayes, D., Hall, A. M., Toomey, E., McDonough, S. M., Lonsdale, C., Walsh, N. E., Guerin, S., & Matthews, J. (2016). Using intervention mapping to develop a theory-driven, group-based complex intervention to support self-management of osteoarthritis and low back pain (SOLAS). IMPLEMENTATION SCIENCE, 11. https://doi.org/10.1186/s13012-016-0418-2 | Focus mainly on pain and not on PAP |
|  | Irwin, M. L. (2009). Physical activity interventions for cancer survivors. British Journal of Sports Medicine, 43(1), 32–38. https://doi.org/10.1136/bjsm.2008.053843 | No PAP-concept |
|  | Javanainen-Levonen, T., Karki, A., & Makitalo, E. (2012). Case study (1997-2011) from Finnish physiotherapy education as a regional promoter of physical activity in special populations. EUROPEAN JOURNAL OF PUBLIC HEALTH, 22, 133–133. | No PAP-concept |
|  | Jensen IB, Nygren A, Lundin A. Cognitive-behavioural treatment for workers with chronic spinal pain: a matched and controlled cohort study in Sweden. Occup Environ Med. 1994 Mar;51(3):145-51. doi: 10.1136/oem.51.3.145. PMID: 8130841; PMCID: PMC1127931. | Focus mainly on pain and not on PAP |
|  | Johansson, A.-C., Linton, S. J., Bergkvist, L., Nilsson, O., & Cornefjord, M. (2009). Clinic-based training in comparison to home-based training after first-time lumbar disc surgery: A randomised controlled trial. European Spine Journal, 18(3), 398–409. https://doi.org/10.1007/s00586-008-0826-3 | No PAP-concept |
|  | Jones, J., Alexander, L., Hancock, E., & Cooper, K. (2021). A collaborative approach to exercise provision for people with Parkinson’s—A feasibility and acceptability study of the PDConnect programme. AMRC Open Research, 2021(2), 29–29. https://doi.org/10.12688/amrcopenres.12936.2 | Exclusively e-health intervention |
|  | Jönsson, T., Ekvall Hansson, E., Thorstensson, C. A., Eek, F., Bergman, P., & Dahlberg, L. E. (2018). The effect of education and supervised exercise on physical activity, pain, quality of life and self-efficacy—An intervention study with a reference group. BMC Musculoskeletal Disorders, 19(1). https://doi.org/10.1186/s12891-018-2098-3 | Focus mainly on pain and not on PAP |
|  | Kanai, M., Izawa, K. P., Kobayashi, M., Onishi, A., Kubo, H., Nozoe, M., Mase, K., & Shimada, S. (2018). Effect of accelerometer-based feedback on physical activity in hospitalized patients with ischemic stroke: A randomized controlled trial. CLINICAL REHABILITATION, 32(8), 1047–1056. https://doi.org/10.1177/0269215518755841 | Focus mainly on disease and not on PAP |
|  | Keel, P. J., Wittig, R., Deutschmann, R., Diethelm, U., Knüsel, O., Löschmann, C., Matathia, R., Rudolf, T., & Spring, H. (1998). Effectiveness of in-patient rehabilitation for sub-chronic and chronic low back pain by an integrative group treatment program (Swiss multicentre study). Scandinavian Journal of Rehabilitation Medicine, 30(4), 211–219. https://doi.org/10.1080/003655098443959 | Focus mainly on pain and not on PAP |
|  | Kennedy N, Larkin L, McKenna S, Pyne T, Gallagher S, Glynn L, Fraser A, Esbensen B. Feasibility of a physiotherapist-led behaviour change intervention to improve physical activity in people with rheumatoid arthritis. Rural Remote Health. 2023 Jan;23(1):8103. doi: 10.22605/RRH8103. Epub 2023 Jan 10. PMID: 36802760. | No PAP-concept |
|  | Kent, K., Johnson, J. D., Simeon, K., & Frates, E. P. (2016). Case Series in Lifestyle Medicine: A Team Approach to Behavior Changes. American Journal of Lifestyle Medicine, 10(6), 388–397. https://doi.org/10.1177/1559827616638288 | Focus mainly on disease and not on PAP |
|  | Kersten, P., McPherson, K. M., Kayes, N. M., Theadom, A., & McCambridge, A. (2015). Bridging the goal intention-action gap in rehabilitation: A study of if-then implementation intentions in neurorehabilitation. Disability and Rehabilitation, 37(12), 1073–1081. https://doi.org/10.3109/09638288.2014.955137 | No PAP-concept |
|  | Kiltz, U., Kiefer, D., Braun, J., Rausch-Osthoff, A. K., Herbold, S., Klinger, M., Kocher, A., Nell-Duxneuner, V., Reichenbach, S., Stamm, T., Steffens-Korbanka, P., & Niedermann, K. (o. J.). Translation of the 2018 EULAR recommendations for physical activity in people with inflammatory arthritis and osteoarthritis and linguistic validation in German-speaking countries with healthcare professionals. ZEITSCHRIFT FUR RHEUMATOLOGIE. https://doi.org/10.1007/s00393-021-01078-0 | No PAP-concept |
|  | Kjaer, P., Kongsted, A., Ris, I., Abbott, A., Rasmussen, C. D. N., Roos, E. M., Skou, S. T., Andersen, T. E., & Hartvigsen, J. (2018). GLA:D-(R) Back group-based patient education integrated with exercises to support self-management of back pain- development, theories and scientific evidence-. BMC MUSCULOSKELETAL DISORDERS, 19. https://doi.org/10.1186/s12891-018-2334-x | Focus mainly on pain and not on PAP |
|  | Koenders, N., van den Heuvel, S., Bloemen, S., van der Wees, P. J., & Hoogeboom, T. J. (2019). Development of a longlist of healthcare quality indicators for physical activity of patients during hospital stay: A modified RAND Delphi study. BMJ OPEN, 9(11). https://doi.org/10.1136/bmjopen-2019-032208 | No PAP-concept |
|  | Kohei, K., Toyohiro, H., Jun, T., Toshiyuki, I., Hiromi, N.-T., Yuji, K., Naoki, N., Michiko, K., & Shigeru, M. (2021). Efficacy of attention bias modification combined with cognitive behavioral therapy for reducing anxiety in patients with hematopoietic malignancies: A quasi-randomized controlled trial. Journal of Affective Disorders Reports, 4. https://doi.org/10.1016/j.jadr.2021.100122 | No PAP-concept |
|  | Koopman, F. S., Beelen, A., Gerrits, K. H., Bleijenberg, G., Abma, T. A., de Visser, M., & Nollet, F. (2010). Exercise therapy and cognitive behavioural therapy to improve fatigue, daily activity performance and quality of life in postpoliomyelitis syndrome: The protocol of the FACTS-2-PPS trial. BMC Neurology, 10, 8–8. https://doi.org/10.1186/1471-2377-10-8 | Focus mainly on disease and not on PAP |
|  | Larkin, L., Mckenna, S., Pyne, T., Gallagher, S., Glynn, L., Fraser, A., Esbensen, B. A., & Kennedy, N. (2022). FEASIBILITY OF A PHYSIOTHERAPIST LED, BEHAVIOUR CHANGE INTERVENTION TO IMPROVE PHYSICAL ACTIVITY IN PEOPLE WITH RHEUMATOID ARTHRITIS. ANNALS OF THE RHEUMATIC DISEASES, 81, 1093–1094. https://doi.org/10.1136/annrheumdis-2022-eular.407 | Conference paper |
|  | Latimer, A. E., Ginis, K. A. M., & Arbour, K. P. (2006). The efficacy of an implementation intention intervention for promoting physical activity among individuals with spinal cord injury: A randomized controlled trial. Rehabilitation Psychology, 51(4), 273–280. https://dx.doi.org/10.1037/0090-5550.51.4.273 | Focus mainly on disease and not on PAP |
|  | Lecat CSY, Fisher A, Atta M, Camilleri M, McCourt O, Land J, Worthington S, Hart A, Daniel A, Uddin I, Roche C, Auner HW, Yong K. High patient satisfaction and increased physical activity following a remote multidisciplinary team multiple myeloma clinic. Support Care Cancer. 2023 Jan 21;31(2):127. doi: 10.1007/s00520-023-07587-9. PMID: 36680643; PMCID: PMC9860216. | Exclusively e-health intervention |
|  | Ledford, C. J. W., Ledford, C. C., & Childress, M. A. (2013). Extending physician ReACH: Influencing patient activation and behavior through multichannel physician communication. Patient Education and Counseling, 91(1), 72–78. https://dx.doi.org/10.1016/j.pec.2012.11.011 | Focus mainly on disease and not on PAP |
|  | Lentz, T. A., Coffman, C. J., Cope, T., Stearns, Z., Simon, C. B., Choate, A., Gladney, M., France, C., Hastings, S. N. & George, S. Z. (2023). If you Build it, Will they Come? Patient and Provider Use of a Novel Hybrid Telehealth Care Pathway for Low Back Pain. *Physical Therapy*, *104*(2). https://doi.org/10.1093/ptj/pzad127 | No PAP-concept |
|  | Lindström, I., Ohlund, C., Eek, C., Wallin, L., Peterson, L. E., Fordyce, W. E., & Nachemson, A. L. (1992). The effect of graded activity on patients with subacute low back pain: a randomized prospective clinical study with an operant-conditioning behavioral approach. Physical therapy, 72(4), 279–293. https://doi.org/10.1093/ptj/72.4.279 | Focus mainly on pain and not on PAP |
|  | Lindström, I., Ohlund, C., Eek, C., Wallin, L., Peterson, L. E., Fordyce, W. E., & Nachemson, A. L. (1992). The effect of graded activity on patients with subacute low back pain: a randomized prospective clinical study with an operant-conditioning behavioral approach. Physical therapy, 72(4), 279–293. https://doi.org/10.1093/ptj/72.4.279 | Focus mainly on pain and not on PAP |
|  | Linmans, J. J., van Rossem, C., Knottnerus, J. A., & Spigt, M. (2015). Exploring the process when developing a lifestyle intervention in primary care for type 2 diabetes: A longitudinal process evaluation. Public Health, 129(1), 52–59. https://dx.doi.org/10.1016/j.puhe.2014.11.004 | No PAP-concept |
|  | Liu, J. Y.-W., Lai, C. K. Y., Siu, P. M., Kwong, E., & Tse Justina Y.-W.; ORCID: https://orcid.org/0000-0003-1931-0159, M. M. Y. A. I.-L. (2017). An individualized exercise programme with and without behavioural change enhancement strategies for managing fatigue among frail older people: A quasi-experimental pilot study. Clinical Rehabilitation, 31(4), 521–531. https://dx.doi.org/10.1177/0269215516649226 | Focus mainly on disease and not on PAP |
|  | Lonsdale, C., Hall, A. M., Murray, A., Williams, G. C., McDonough, S. M., Ntoumanis, N., Owen, K., Schwarzer, R., Parker, P., Kolt, G. S., & Hurley, D. A. (2017). Communication Skills Training for Practitioners to Increase Patient Adherence to Home-Based Rehabilitation for Chronic Low Back Pain: Results of a Cluster Randomized Controlled Trial. Archives of Physical Medicine and Rehabilitation, 98(9), 1732-1743.e7. https://doi.org/10.1016/j.apmr.2017.02.025 | Focus mainly on pain and not on PAP |
|  | Lonsdale, C., Hall, A. M., Williams, G. C., McDonough, S. M., Ntoumanis, N., Murray, A., & Hurley, D. A. (2012). Communication style and exercise compliance in physiotherapy (CONNECT). A cluster randomized controlled trial to test a theory-based intervention to increase chronic low back pain patients’ adherence to physiotherapists’ recommendations: Study rationale, design, and methods. BMC MUSCULOSKELETAL DISORDERS, 13. https://doi.org/10.1186/1471-2474-13-104 | Focus mainly on pain and not on PAP |
|  | Lontano, A., Marziali, E., Galletti, C., Mazza, E., Gambioli, S., Galasso, V., Mingarelli, A., D'Ambrosio, F., Tamburrano, A., Paolini, M., Bande, A., Damiani, G., de Waure, C., Laurenti, P. A real opportunity to modify cardiovascular risk through primary care and prevention: A pilot study. Front Public Health. 2023 Jan 10;10:1009246. doi: 10.3389/fpubh.2022.1009246. PMID: 36703856; PMCID: PMC9871452 | TIDier (insufficient reporting) |
|  | Ma, J. K., Cheifetz, O., Todd, K. R., Chebaro, C., Phang, S., Shaw, R. B., Whaley, K. J., & Ginis, K. A. M. (2020). Co-development of a physiotherapist-delivered physical activity intervention for adults with spinal cord injury. SPINAL CORD, 58(7), 778–786. https://doi.org/10.1038/s41393-020-0422-x | Concept already included |
|  | Ma, J. K., Walden, K., McBride, C. B., Levett, C. L., Colistro, R., Plashkes, T., Thorson, T., Shu, H. T., & Ginis, K. A. M. (2022). Implementation of the spinal cord injury exercise guidelines in the hospital and community settings: Protocol for a type II hybrid trial. SPINAL CORD, 60(1), 53–57. https://doi.org/10.1038/s41393-021-00685-7 | Concept already included |
|  | Ma, Jasmin K.; West, Christopher R.; Martin Ginis, Kathleen A. . (2019). The Effects of a Patient and Provider Co-Developed, Behavioral Physical Activity Intervention on Physical Activity, Psychosocial Predictors, and Fitness in Individuals with Spinal Cord Injury: A Randomized Controlled Trial. Sports Medicine, (), –. doi:10.1007/s40279-019-01118-5 | Concept already included |
|  | MacKay-Lyons, M., Gubitz, G., Giacomantonio, N., Wightman, H., Marsters, D., Thompson, K., Blanchard, C., Eskes, G., & Thornton, M. (2010). Program of rehabilitative exercise and education to avert vascular events after non-disabling stroke or transient ischemic attack (PREVENT Trial): A multi-centred, randomised controlled trial. BMC Neurology, 10. https://dx.doi.org/10.1186/1471-2377-10-122 | No PAP-concept |
|  | Malfliet, A., Bilterys, T., Van Looveren, E., Meeus, M., Danneels, L., Ickmans, K., Cagnie, B., Mairesse, O., Neu, D., Moens, M., Goubert, D., Kamper, S. J., & Nijs, J. (2019). The added value of cognitive behavioral therapy for insomnia to current best evidence physical therapy for chronic spinal pain: Protocol of a randomized controlled clinical trial. BRAZILIAN JOURNAL OF PHYSICAL THERAPY, 23(1), 62–70. https://doi.org/10.1016/j.bjpt.2018.10.007 | No PAP-concept |
|  | Mansfield, A., Brooks, D., Tang, A., Taylor, D., Inness, E. L., Kiss, A., Middleton, L., Biasin, L., Fleck, R., French, E., LeBlanc, K., Aqui, A., & Danells, C. (2017). Promoting Optimal Physical Exercise for Life (PROPEL): Aerobic exercise and self-management early after stroke to increase daily physical activity-study protocol for a stepped-wedge randomised trial. BMJ Open, 7(6), e015843–e015843. https://doi.org/10.1136/bmjopen-2017-015843 | Focus mainly on disease and not on PAP |
|  | Mansfield, A., Knorr, S., Poon, V., Inness, E. L., Middleton, L., Biasin, L., Brunton, K., Howe, J. A., & Brooks, D. (2016). Promoting Optimal Physical Exercise for Life: An Exercise and Self-Management Program to Encourage Participation in Physical Activity after Discharge from Stroke Rehabilitation-A Feasibility Study. Stroke research and treatment, 2016, 9476541. https://doi.org/10.1155/2016/9476541 | TIDier (insufficient reporting) |
|  | Martini, D. N., Zeeboer, E., Hildebrand, A., Fling, B. W., Hugos, C. L., & Cameron, M. H. (2018). ADSTEP: Preliminary Investigation of a Multicomponent Walking Aid Program in People With Multiple Sclerosis. Archives of Physical Medicine and Rehabilitation, 99(10), 2050–2058. https://doi.org/10.1016/j.apmr.2018.05.023 | Focus mainly on disease and not on PAP |
|  | Maruthur NM, Wang NY, Appel LJ. (2009). Lifestyle interventions reduce coronary heart disease risk: results from the PREMIER Trial. Circulation. 2009 Apr 21;119(15):2026-31. doi: 10.1161/CIRCULATIONAHA.108.809491. Epub 2009 Apr 6. PMID: 19349322; PMCID: PMC2995494. | No PAP-concept |
|  | Master, H., Coronado, R. A., Whitaker, S., Block, S., Vanston, S. W., Pennings, J. S., Gupta, R., Robinette, P., Stephens, B., Abtahi, A., Schwarz, J. & Archer, K. R. (2023). Combining Wearable Technology and Telehealth Counseling for Rehabilitation after Lumbar Spine Surgery: Feasibility and Acceptability of a Physical Activity Intervention. *Physical Therapy*, *104*(2). https://doi.org/10.1093/ptj/pzad096 | TIDier (insufficient reporting) |
|  | McCourt, O., Fisher, A., Ramdharry, G., Roberts, A. L., Land, J., Rabin, N., & Yong, K. (2020). PERCEPT myeloma: A protocol for a pilot randomised controlled trial of exercise prehabilitation before and during autologous stem cell transplantation in patients with multiple myeloma. BMJ OPEN, 10(1). https://doi.org/10.1136/bmjopen-2019-033176 | No PAP-concept |
|  | McGrane, N., Cusack, T., O’Donoghue, G., & Stokes, E. (2014). Motivational strategies for physiotherapists. Physical Therapy Reviews, 19(2), 136–142. https://doi.org/10.1179/1743288X13Y.0000000117 | No PAP-concept |
|  | Michie, S. , Ashford, S. , Sniehotta, F.F. , Dombrowski, S.U. , Bishop, A. and French, D.P. (2011). A refined taxonomy of behaviour change techniques to help people change their physical activity and healthy eating behaviours: The CALO-RE taxonomy. Psychology & Health, volume 26 (11): 1479-1498. http://dx.doi.org/10.1080/08870446.2010.540664 | No PAP-concept |
|  | Miller, W. R., & Rollnick, S. (2009). Ten things that motivational interviewing is not. Behavioural and cognitive psychotherapy, 37(2), 129–140. https://doi.org/10.1017/S1352465809005128 | No PAP-concept |
|  | Moore SA, Flynn D, Jones S, Price CIM, Avery L. (2022) Feasibility, acceptability, and fidelity of Physical Activity Routines After Stroke (PARAS): a multifaceted behaviour change intervention targeting free-living physical activity and sedentary behaviour in community-dwelling adult stroke survivors. Pilot Feasibility Stud. 2022 Sep 3;8(1):197. doi: 10.1186/s40814-022-01139-4. PMID: 36057723; PMCID: PMC9440503. | Concept already included |
|  | Morris, J. H., Oliver, T., Kroll, T., Joice, S., & Williams, B. (2017). Physical activity participation in community dwelling stroke survivors: Synergy and dissonance between motivation and capability. A qualitative study. PHYSIOTHERAPY, 103(3), 311–321. https://doi.org/10.1016/j.physio.2016.05.001 | No PAP-concept |
|  | Mulderij, L. S. (2022). Care to move! A mixed-methods evaluation of care–physical activity initiatives for citizens with a low socioeconomic status. [internal PhD, WU, Wageningen University]. Wageningen University. https://doi.org/10.18174/567262 | Wrong setting |
|  | Mulligan, H., Treharne, G. J., Hale, L. A., & Smith, C. (2013). Combining Self-help and Professional Help to Minimize Barriers to Physical Activity in Persons With Multiple Sclerosis: A Trial of the „Blue Prescription“ Approach in New Zealand. JOURNAL OF NEUROLOGIC PHYSICAL THERAPY, 37(2), 51–57. https://doi.org/10.1097/NPT.0b013e318292799e | Focus mainly on disease and not on PAP |
|  | Ngouali, T. M., Borjesson, M., Cider, A., & Lundqvist, S. (2021). Nonresponders of Physical Activity on Prescription (PAP) Can Increase Their Exercise Capacity with Enhanced Physiotherapist Support. INTERNATIONAL JOURNAL OF ENVIRONMENTAL RESEARCH AND PUBLIC HEALTH, 18(9). https://doi.org/10.3390/ijerph18094795 | No PAP-concept |
|  | Noland M. P. (1989). The effects of self-monitoring and reinforcement on exercise adherence. Research quarterly for exercise and sport, 60(3), 216–224. https://doi.org/10.1080/02701367.1989.10607443 | No PAP-concept |
|  | Nooijen, C. F. J., Stam, H. J., Bergen, M. P., Bongers-Janssen, H. M. H., Valent, L., van Langeveld, S., Twisk, J., van den Berg-Emons, R. J. G., & Grp, A.-A. R. (2016). A behavioural intervention increases physical activity in people with subacute spinal cord injury: A randomised trial. JOURNAL OF PHYSIOTHERAPY, 62(1), 35–41. https://doi.org/10.1016/j.jphys.2015.11.003 | Focus mainly on disease and not on PAP |
|  | Nordbrandt, M. S., Sonne, C., Mortensen, E. L., & Carlsson, J. (2020). Trauma-affected refugees treated with basic body awareness therapy or mixed physical activity as augmentation to treatment as usual-A pragmatic randomised controlled trial. PloS one, 15(3), e0230300. https://doi.org/10.1371/journal.pone.0230300 | No PAP-concept |
|  | Okkersen, K., Jimenez-Moreno, C., Wenninger, S., Daidj, F., Glennon, J., Cumming, S., Littleford, R., Monckton, D. G., Lochmuller, H., Catt, M., Faber, C. G., Hapca, A., Donnan, P. T., Gorman, G., Bassez, G., Schoser, B., Knoop, H., Treweek, S., van Engelen, B. G. M., & Consortium, O. (2018). Cognitive behavioural therapy with optional graded exercise therapy in patients with severe fatigue with myotonic dystrophy type 1: A multicentre, single-blind, randomised trial. LANCET NEUROLOGY, 17(8), 671–680. https://doi.org/10.1016/S1474-4422(18)30203-5 | Focus mainly on disease and not on PAP |
|  | Oliveira, C. B., Christofaro, D. G. D., Maher, C. G., Franco, M. R., Tiedemann, A., Silva, F. G., Damato, T. M., Nicholas, M. K., & Pinto, R. Z. (2022). Adding Physical Activity Coaching and an Activity Monitor Was No More Effective Than Adding an Attention Control Intervention to Group Exercise for Patients With Chronic Nonspecific Low Back Pain (PAYBACK Trial): A Randomized Trial. JOURNAL OF ORTHOPAEDIC & SPORTS PHYSICAL THERAPY, 52(5), 287–299. https://doi.org/10.2519/jospt.2022.10874 | Focus mainly on pain and not on PAP |
|  | Oliveira, C. B., Franco, M. R., Maher, C. G., Tiedemann, A., Silva, F. G., Damato, T. M., Nicholas, M. K., Christofaro, D. G. D., & Pinto, R. Z. (2018). The efficacy of a multimodal physical activity intervention with supervised exercises, health coaching and an activity monitor on physical activity levels of patients with chronic, nonspecific low back pain (Physical Activity for Back Pain (PAyBACK) trial): Study protocol for a randomised controlled trial. TRIALS, 19. https://doi.org/10.1186/s13063-017-2436-z | No PAP-concept |
|  | Orme, M. W., Clague-Baker, N. J., Richardson, M., Drewry, S., Robinson, T. G., & Singh, S. J. (2020). Does cardiac rehabilitation for people with stroke in the sub-acute phase of recovery lead to physical behaviour change? Results from compositional analysis of accelerometry-derived data. PHYSIOTHERAPY, 107, 234–242. https://doi.org/10.1016/j.physio.2019.10.003 | No PAP-concept |
|  | Ory, M. G., Lee, S., Zollinger, A., Bhurtyal, K., Jiang, L., & Smith, M. L. (2015). Translation of Fit & Strong! For middle-aged and older adults: Examining implementation and effectiveness of a lay-led model in Central Texas. Frontiers in Public Health, 2, 187–187. https://doi.org/10.3389/fpubh.2014.00187 | No PAP-concept |
|  | Ostelo, R. W., de Vet, H. C., Waddell, G., Kerckhoffs, M. R., Leffers, P., & van Tulder, M. (2003). Rehabilitation following first-time lumbar disc surgery: a systematic review within the framework of the cochrane collaboration. Spine, 28(3), 209–218. https://doi.org/10.1097/01.BRS.0000042520.62951.28 | Focus mainly on pain and not on PAP |
|  | Østerås, N., van Bodegom-Vos, L., Dziedzic, K., Moseng, T., Aas, E., Andreassen, Ø., Mdala, I., Natvig, B., Røtterud, J. H., Schjervheim, U.-B., Vlieland, T. V., & Hagen, K. B. (2015). Implementing international osteoarthritis treatment guidelines in primary health care: Study protocol for the SAMBA stepped wedge cluster randomized controlled trial. Implementation Science : IS, 10, 165–165. https://doi.org/10.1186/s13012-015-0353-7 | TIDier (insufficient reporting) |
|  | Osthoff, A. K. R., Beyer, S., Gisi, D., Rezek, S., Schwank, A., Meichtry, A., Sievi, N. A., Hess, T., Wirz, M., Rausch Osthoff, A.-K., Beyer, S., Gisi, D., Rezek, S., Schwank, A., Meichtry, A., Sievi, N. A., Hess, T., & Wirz, M. (2021). Effect of counselling during pulmonary rehabilitation on self-determined motivation to be physically active for people with chronic obstructive pulmonary disease: A pragmatic RCT. BMC PULMONARY MEDICINE, 21(1). https://doi.org/10.1186/s12890-021-01685-2 | Focus mainly on disease and not on PAP |
|  | Osthoff, A. K., Greco, N., Schwank, A., Beyer, S., Gisi, D., Scheermesser, M., Meichtry, A., Sievi, N., Hess, T., & Wirz, M. (2017). Effect of counselling during pulmonary rehabilitation on self-determined motivation towards physical activity in people with chronic obstructive pulmonary disease - protocol of a mixed methods study. BMC pulmonary medicine, 17(1), 115. https://doi.org/10.1186/s12890-017-0457-8 | Focus mainly on disease and not on PAP |
|  | Paiva, L. G., De Oliveira, T. M. D., De Souza, N. B., Alberto, K. C., Almeida, D. P., Oliveira, C. C., José, A. & Malaguti, C. (2024). Exploring the impact of the environment on physical activity in patients with chronic obstructive pulmonary disease (EPCOT)—A comparative analysis between suggested and free walking: Protocol study. *PLoS ONE*, *19*(8), e0306045. https://doi.org/10.1371/journal.pone.0306045 | Wrong setting |
|  | Pang, M. Y. C. (2012). The role of physiotherapy in prevention and management of lifestyle-related conditions. Hong Kong Physiotherapy Journal, 30(2), 43–43. https://doi.org/10.1016/j.hkpj.2012.08.001 | No PAP-concept |
|  | Parchment A, Lawrence W, Rahman E, Townsend N, Wainwright E, Wainwright D. 'I can feel myself coming out of the rut': a brief intervention for supporting behaviour change is acceptable to patients with chronic musculoskeletal conditions. BMC Musculoskelet Disord. 2023 Mar 29;24(1):241. doi: 10.1186/s12891-023-06336-7. PMID: 36991425; PMCID: PMC10050805. | No PAP-concept |
|  | Parra, D. C., Bradford, E. C. H., Clark, B. R., Racette, S. B., & Deusinger, S. S. (2017). Population and Community-Based Promotion of Physical Activity: A Priority for Physical Therapy. PHYSICAL THERAPY, 97(2), 159–160. https://doi.org/10.1093/ptj/pzw006 | No PAP-concept |
|  | Pellegrini, C. A., Brown, D., DeVivo, K. E., Lee, J., & Wilcox, S. (2022). Promoting Physical Activity Via Physical Therapist Following Knee Replacement: A Pilot Randomized Controlled Trial. PM & R : The Journal of Injury, Function, and Rehabilitation. https://doi.org/10.1002/pmrj.12895 | No PAP-concept |
|  | Peolsson, A., Ludvigsson, M. L., Overmeer, T., Dedering, A., Bernfort, L., Johansson, G., Kammerlind, A. S., & Peterson, G. (2013). Effects of neck-specific exercise with or without a behavioural approach in addition to prescribed physical activity for individuals with chronic whiplash-associated disorders: A prospective randomised study. BMC MUSCULOSKELETAL DISORDERS, 14. https://doi.org/10.1186/1471-2474-14-311 | No PAP-concept |
|  | Peterson, G. E., Landén Ludvigsson, M. H., O’Leary, S. P., Dedering, Å. M., Wallman, T., Jönsson, M. I. N., & Peolsson, A. L. C. (2015). The effect of 3 different exercise approaches on neck muscle endurance, kinesiophobia, exercise compliance, and patient satisfaction in chronic whiplash. Journal of Manipulative and Physiological Therapeutics, 38(7), 465-476.e4. https://doi.org/10.1016/j.jmpt.2015.06.011 | No PAP-concept |
|  | Petrusevski, C., MacDermid, J. C., Wilson, M. G. & Richardson, J. (2024). Framing Physical Literacy for Adults Through a Rehabilitation Lens: An Expert Consensus Study. *Journal Of Aging And Physical Activity*, 1–8. https://doi.org/10.1123/japa.2023-0095 | No PAP-concept |
|  | Pfeifer K, Hofmann J, Buchmann J, Meng K, Vogel H, Faller H, Bork (2013). H:Langfristige Wirksamkeit einer integrativen Patientenschulung zurOptimierung der stationären Rehabilitation bei chronischemRückenschmerz (PASTOR)–eine multizentrische, prospektiveKontrollgruppenstudie (Long-term effectiveness of an integrative patienteducation to the optimisation of inpatient rehabilitation in chronic low-back pain (PASTOR)–a prospective study with a control group).InTagungsband des 21.Edited by Deutsche Rentenversicherung Bund.Hamburg: Rehabilitationswissenschaftlichen Kolloquiums: 5.-7. März 2012;2012:370–373. | Focus mainly on pain and not on PAP |
|  | Pfeifer, K., Huber, G., Baldus, A., Pothig, D., & Schule, K. (2012). Resource management: ICF-oriented exercise programs for patients with diabetes mellitus type 2. Chronic illnesses and biopsychosocial status. Ressourcenmanagement: ICF-orientierte bewegungsprogramme fur personen mit diabetes mellitus typ 2: Chronische erkrankungen und biopsychosozialer status., 45(2), 119–127. *Zeitschrift für Gerontologie und Geriatrie*. https://dx.doi.org/10.1007/s00391-011-0276-0 | Focus mainly on disease and not on PAP |
|  | Pfeifer, K., Sudeck, G., Brüggemann, S., & Huber, G. (2010). DGRW-Update: Bewegungstherapie in der medizinischen Rehabilitation – Wirkungen, Qualität, Perspektiven. Die Rehabilitation, 49(04), 224–236. https://doi.org/10.1055/s-0030-1261909 | No PAP-concept |
|  | Porserud, A., Karlsson, P., Rydwik, E., Aly, M., Henningsohn, L., Nygren-Bonnier, M., & Hagstromer, M. (2020). The CanMoRe trial—Evaluating the effects of an exercise intervention after robotic-assisted radical cystectomy for urinary bladder cancer: The study protocol of a randomised controlled trial. BMC CANCER, 20(1). https://doi.org/10.1186/s12885-020-07140-5 | TIDier (insufficient reporting) |
|  | Primdahl, J., Bremander, A., Hendricks, O., Østergaard, M., Latocha, K. M., Andersen, L., Jensen, K. V. & Esbensen, B. A. (2024). Development of a complex Interdisciplinary Nurse-coordinated SELf-MAnagement (INSELMA) intervention for patients with inflammatory arthritis. *BMC Health Services Research*, *24*(1). https://doi.org/10.1186/s12913-023-10463-1 | Wrong setting |
|  | ProACTIVESCI Team. (2019). A Physiotherapist’s Guide to Promoting Physical Activity to Clients who have Spinal Cord. University of British Columbia. https://fhsd-sciactioncanada-2019.sites.olt.ubc.ca/files/2019/12/ProacTive_SCI_Toolkit_Nov.pdf | Concept already included |
|  | Quinn, A., Doody, C., & O’Shea, D. (2008). The effect of a physical activity education programme on physical activity, fitness, quality of life and attitudes to exercise in obese females. Journal of Science and Medicine in Sport, 11(5), 469–472. https://dx.doi.org/10.1016/j.jsams.2007.07.011 | No PAP-concept |
|  | Rafferty MR, Held Bradford EC, Fritz S, Hutchinson KJ, Miczak K, Resnick A, Billinger SA. Health Promotion and Wellness in Neurologic Physical Therapy: Strategies to Advance Practice. J Neurol Phys Ther. 2022 Apr 1;46(2):103-117. doi: 10.1097/NPT.0000000000000376. PMID: 34507339; PMCID: PMC8904651. | No PAP-concept |
|  | Rafferty, M. R., MacDonald, J., Byskosh, A., Sloan, L., Toledo, S., Marciniak, C., & Simuni, T. (2019). Using Implementation Frameworks to Provide Proactive Physical Therapy for People With Parkinson Disease: Case Report. PHYSICAL THERAPY, 99(12), 1644–1655. https://doi.org/10.1093/ptj/pzz129 | Focus mainly on disease and not on PAP |
|  | Ramaswamy, B. (2012). Adherence to physical activity through the development of a physiotherapy-led „exercise community“. JOURNAL OF AGING AND PHYSICAL ACTIVITY, 20, S223–S223. | No PAP-concept |
|  | Rasson, S. (2022). Reducing Eating disorders with a multidisciplinary intervention containing cognitive therapy, nutrition and physical exercise in overweighed adults between years 2016 and 2018. Annales Medico-Psychologiques, 180(6), 495–502. https://doi.org/10.1016/j.amp.2020.12.015 | Wrong language |
|  | Rausch-Osthoff, A. K., Greco, N., Schwank, A., Beyer, S., Gisi, D., Scheermesser, M., Meichtry, A., Sievi, N., Hess, T., & Wirz, M. (2017). Effect of counselling during pulmonary rehabilitation on self-determined motivation towards physical activity in people with chronic obstructive pulmonary disease - protocol of a mixed methods study. BMC pulmonary medicine, 17(1), 115. https://doi.org/10.1186/s12890-017-0457-8 | Focus mainly on disease and not on PAP |
|  | Rejeski, W. J., Brawley, L. R., Ambrosius, W. T., Brubaker, P. H., Focht, B. C., Foy, C. G., & Fox, L. D. (2003). Older adults with chronic disease: Benefits of group-mediated counseling in the promotion of physically active lifestyles. Health Psychology, 22(4), 414–423. https://dx.doi.org/10.1037/0278-6133.22.4.414 | Concept already included |
|  | Research Unit for Musculoskeletal Science and Clinical Biomechanics. (2015). GLAD annual report 2015: GLA registry. https://www.glaid.dk | Focus mainly on disease and not on PAP |
|  | Rhodes S, Waters D, Brockway B, Skinner M. Exercise and motivational text messaging to support physical activity behaviour change in a population with obstructive sleep apnoea: a feasibility study. J Prim Health Care. 2022 Dec;14(4):318-325. doi: 10.1071/HC22033. PMID: 36592765. | Focus mainly on disease and not on PAP |
|  | Ridgel, A. L., Walter, B. L., Tatsuoka, C., Walter, E. M., Colon-Zimmermann, K., Welter, E., & Sajatovic, M. (2016). Enhanced Exercise Therapy in Parkinson’s disease: A comparative effectiveness trial. JOURNAL OF SCIENCE AND MEDICINE IN SPORT, 19(1), 12–17. https://doi.org/10.1016/j.jsams.2015.01.005 | No PAP-concept |
|  | Robson, E. K., Kamper, S. J., Davidson, S., Viana Da Silva, P., Williams, A., Hodder, R. K., Lee, H., Hall, A., Gleadhill, C., & Williams, C. M. (2019). Healthy Lifestyle Program (HeLP) for low back pain: Protocol for a randomised controlled trial. BMJ Open, 9(9), e029290. https://doi.org/10.1136/bmjopen-2019-029290 | Focus mainly on pain and not on PAP |
|  | Rollnick, S., Miller, W. R., & Butler, C. C. (2023). Motivational interviewing in health care: Helping patients change behavior. Guilford Press | No PAP-concept |
|  | Rundell, S. D., & Davenport, T. E. (2010). Patient Education Based on Principles of Cognitive Behavioral Therapy for a Patient With Persistent Low Back Pain: A Case Report. JOURNAL OF ORTHOPAEDIC & SPORTS PHYSICAL THERAPY, 40(8), 494–501. https://doi.org/10.2519/jospt.2010.3264 | Focus mainly on pain and not on PAP |
|  | Ryan JM, Fortune J, Stennett A, et al. (2017). Changing physical activity behaviour for people with multiple sclerosis: protocol of a randomised controlled feasibility trial (iStep-MS). BMJ Open2017;7:e018875. doi:10.1136/bmjopen-2017-018875 | Concept already included |
|  | Ryan, J. M., Fortune, J., Stennett, A., Kilbride, C., Lavelle, G., Hendrie, W., DeSouza, L., Abdul, M., Brewin, D., David, L., Anokye, N., Victor, C., & Norris, M. (2020). Safety, feasibility, acceptability and effects of a behaviour-change intervention to change physical activity behaviour among people with multiple sclerosis: Results from the iStep-MS randomised controlled trial. MULTIPLE SCLEROSIS JOURNAL, 26(14), 1907–1918. https://doi.org/10.1177/1352458519886231 | Concept already included |
|  | Salinas C., J., Bello S., S., Chamorro R., H., & Gonzalez G., C. G. (2016). Counseling in feeding, physical activity and tobacco. Basic instrument in professional practice. Revista Chilena de Nutricion, 43(4), 434–442. https://doi.org/10.4067/S0717-75182016000400015 | Wrong language |
|  | Sarah, J. E., Osiac, L. R., Quevedo, T. P., Martin, M. A., & Samur, E. A. (2011). IMPACT OF A NATIONAL TREATMENT PROGRAM IN OVERWEIGHT ADULTS WOMEN IN PRIMARY CARE CENTERS. NUTRICION HOSPITALARIA, 26(6), 1372–1377. https://doi.org/10.3305/nh.2011.26.6.5272 | Wrong language |
|  | Schlegel, S., Hafner, D., Hartmann, A., Fuchs, R., & Zeeck Armin; ORCID: https://orcid.org/0000-0002-4158-6576, A. A. I.-H. (2012). Sports therapy for outpatients with eating disorders: A pilot project. Ambulante Sporttherapie fur Patientinnen mit Essstorungen: Ein Pilotprojekt., 62(12), 456–462. https://dx.doi.org/10.1055/s-0032-1316370 | No PAP-concept |
|  | Scholz, U., Knoll, N., Sniehotta, F. F., & Schwarzer, R. (2006). Physical activity and depressive symptoms in cardiac rehabilitation: long-term effects of a self-management intervention. *Social Science & Medicine (1982)*, *62*(12), 3109–3120. doi:10.1016/j.socscimed.2005.11.035 | Focus mainly on disease and not on PAP |
|  | Schreurs, K. M. G., Veehof, M. M., Passade, L., & Vollenbroek-Hutten, M. M. R. (2011). Cognitive behavioural treatment for chronic fatigue syndrome in a rehabilitation setting: Effectiveness and predictors of outcome. Behaviour Research and Therapy, 49(12), 908–913. https://doi.org/10.1016/j.brat.2011.09.004 | No PAP-concept |
|  | Schroé H, Van Dyck D, De Paepe A, Poppe L, Loh WW, Verloigne M, Loeys T, De Bourdeaudhuij I, Crombez G. (2020). Which behaviour change techniques are effective to promote physical activity and reduce sedentary behaviour in adults: a factorial randomized trial of an e- and m-health intervention. Int J Behav Nutr Phys Act. 2020 Oct 7;17(1):127. doi: 10.1186/s12966-020-01001-x. PMID: 33028335; PMCID: PMC7539442. | Exclusively e-health intervention |
|  | Schwaab B, Henke N, Guha M, Schlitt A, Müller-Werdan U, Edelmann F, von Haehling S, Landmesser U, Pauschinger M. (2023). Kardiologische Rehabilitation bei Patienten mit Herzinsuffizienz: Gemeinsame Empfehlungen der Deutschen Gesellschaft für Kardiologie (DGK) und der Deutschen Gesellschaft für Prävention und Rehabilitation von Herz-Kreislauferkrankungen (DGPR) [Cardiac rehabilitation in patients with heart failure: Joint recommendations of the German Cardiac Society (DGK) and the German Society for Prevention and Rehabilitation of Cardiovascular Diseases (DGPR)]. Kardiologie. 2023;17(3):161–72. German. doi: 10.1007/s12181-023-00611-6. Epub 2023 May 17. PMCID: PMC10191098. | No PAP-concept |
|  | Semrau, J., Hentschke, C., Peters, S., & Pfeifer, K. (2021). Effects of behavioural exercise therapy on the effectiveness of multidisciplinary rehabilitation for chronic non-specific low back pain: A randomised controlled trial. BMC MUSCULOSKELETAL DISORDERS, 22(1). https://doi.org/10.1186/s12891-021-04353-y | Focus mainly on pain and not on PAP |
|  | Serón, P., Oliveros, M. J., Marzuca-Nassr, G. N., Lanas, F., Morales, G., Román, C., Muñoz, S. R., Saavedra, N., & Grace, S. L. (2019). Hybrid cardiac rehabilitation trial (HYCARET): Protocol of a randomised, multicentre, non-inferiority trial in South America. BMJ Open, 9(10), e031213–e031213. https://doi.org/10.1136/bmjopen-2019-031213 | Exclusively e-health intervention |
|  | Severin, R., Sabbahi, A., Arena, R., & Phillips, S. A. (2022). Precision Medicine and Physical Therapy: A Healthy Living Medicine Approach for the Next Century. Physical Therapy, 102(1). https://doi.org/10.1093/ptj/pzab253 | No PAP-concept |
|  | Seymour, R. B., Hughes, S. L., Campbell, R. T., Huber, G. M., & Desai, P. (2009). Comparison of Two Methods of Conducting the Fit and Strong! Program. ARTHRITIS & RHEUMATISM-ARTHRITIS CARE & RESEARCH, 61(7), 876–884. https://doi.org/10.1002/art.24517 | Focus mainly on disease and not on PAP |
|  | Sheedy, J., Smith, B., Bauman, A., Barnett, A., Calderan, A., Culbert, J., & Jacka, J. (2000). A controlled trial of behavioural education to promote exercise among physiotherapy outpatients. AUSTRALIAN JOURNAL OF PHYSIOTHERAPY, 46(4), 281–289. https://doi.org/10.1016/S0004-9514(14)60289-0 | TIDier (insufficient reporting) |
|  | Sherrington, C., Fairhall, N., Kirkham, C., Clemson, L., Howard, K., Vogler, C., Close, J. C. T., Moseley, A. M., Cameron, I. D., Mak, J., Sonnabend, D., & Lord, S. R. (2016). Exercise and fall prevention self-management to reduce mobility-related disability and falls after fall-related lower limb fracture in older people: Protocol for the RESTORE (Recovery Exercises and STepping On afteR fracturE) randomised controlled trial. BMC GERIATRICS, 16. https://doi.org/10.1186/s12877-016-0206-5 | Focus mainly on disease and not on PAP |
|  | Silva, I., Moreira, C. S., Pedras, S., Oliveira, R., Veiga, C., Moreira, L., Santarém, D., Guedes, D. & Paredes, H. (2023). Effect of a monitored home-based exercise program combined with a behavior change intervention and a smartphone app on walking distances and quality of life in adults with peripheral arterial disease: the WalkingPad randomized clinical trial. *Frontiers in Cardiovascular Medicine*, *10*. https://doi.org/10.3389/fcvm.2023.1272897 | TIDier (insufficient reporting) |
|  | Sjöquist, E. S., Brodin, N., Lampa, J., Jensen, I., & Opava, C. H. (2011). Physical activity coaching of patients with rheumatoid arthritis in everyday practice: A long-term follow-up. Musculoskeletal Care, 9(2), 75–85. https://doi.org/10.1002/msc.199 | Focus mainly on disease and not on PAP |
|  | Skolasky, R. L., Riley, L. H., Maggard, A. M., Bedi, S., & Wegener, S. T. (2013). Functional recovery in lumbar spine surgery: A controlled trial of health behavior change counseling to improve outcomes. CONTEMPORARY CLINICAL TRIALS, 36(1), 207–217. https://doi.org/10.1016/j.cct.2013.06.018 | No PAP-concept |
|  | Skou, S. T., & Roos, E. M. (2017). Good Life with osteoArthritis in Denmark (GLA:D (TM)): Evidence-based education and supervised neuromuscular exercise delivered by certified physiotherapists nationwide. BMC MUSCULOSKELETAL DISORDERS, 18. https://doi.org/10.1186/s12891-017-1439-y | Focus mainly on disease and not on PAP |
|  | Smith, O. T, Scott Parsons, Beth Fordham, Alexander Ooms, Susan Dutton, Caroline Hing, Vicki S Barber, May Ee Png, & Sarah Lamb. (2020). Behaviour change physiotherapy intervention to increase physical activity following hip and knee replacement (PEP-TALK): Study protocol for a pragmatic randomised controlled trial. BMJ Open, 10(7), e035014. https://doi.org/10.1136/bmjopen-2019-035014 | TIDier (insufficient reporting) |
|  | Smith, T. O., Parsons, S., Ooms, A., Dutton, S., Fordham, B., Garrett, A., Hing, C., Lamb, S., & Collaborators, P.-T. T. (2022). Randomised controlled trial of a behaviour change physiotherapy intervention to increase physical activity following hip and knee replacement: The PEP-TALK trial. BMJ OPEN, 12(5). https://doi.org/10.1136/bmjopen-2022-061373 | TIDier (insufficient reporting) |
|  | Sniehotta, F. F., Schwarzer, R., Scholz, U., & Schüz, B. (2005). Action planning and coping planning for long-term lifestyle change: theory and assessment. *European Journal of Social Psychology*, *35*(4), 565–576. doi:10.1002/ejsp.258 | No PAP-concept |
|  | Söderlund, A., & Lindberg, P. (2001). An integrated physiotherapy/cognitive-behavioural approach to the analysis and treatment of chronic Whiplash Associated Disorders, WAD. Disability and Rehabilitation, 23(10), 436–447. https://doi.org/10.1080/09638280010008870 | No PAP-concept |
|  | Speelman, A. D., van Nimwegen, M., Bloem, B. R., & Munneke, M. (2014). Evaluation of implementation of the Park Fit program: A multifaceted intervention aimed to promote physical activity in patients with Parkinson’s disease. PHYSIOTHERAPY, 100(2), 134–141. https://doi.org/10.1016/j.physio.2013.05.003 | Concept already included |
|  | Stevens, A., Köke, A., van der Weijden, T., & Beurskens, A. (2017). Ready for goal setting? Process evaluation of a patient-specific goal-setting method in physiotherapy. BMC Health Services Research, 17(1), 618–618. https://doi.org/10.1186/s12913-017-2557-9 | Concept already included |
|  | Sullivan, M. B., Hill, K., Ballengee, L. A., Knoblach, D., Fowler, C., Haun, J. & Saenger, M. (2023). Remotely Delivered Psychologically Informed Mindful Movement Physical Therapy for Pain Care: A Framework for Operationalization. *Global Advances in Integrative Medicine And Health*, *12*. https://doi.org/10.1177/27536130231209751 | Focus mainly on pain and not on PAP |
|  | Taricco, M., Dallolio, L., Calugi, S., Rucci, P., Fugazzaro, S., Stuart, M., Pillastrini, P., Fantini, M. P., & Esercizio Fisico di Gruppo/2009 Investigators (2014). Impact of adapted physical activity and therapeutic patient education on functioning and quality of life in patients with postacute strokes. Neurorehabilitation and neural repair, 28(8), 719–728. https://doi.org/10.1177/1545968314523837 | No PAP-concept |
|  | Taylor, S., Barker, K., Stephensen, D. & Williamson, E. (2024). Using evidence‐based co‐design to develop a hybrid delivered exercise intervention that aims to increase confidence to exercise in people with haemophilia. *Haemophilia*. https://doi.org/10.1111/hae.14972 | Focus mainly on disease and not on PAP |
|  | Tegner, H., Esbensen, B. A., Henriksen, M., Bech-Azeddine, R., Lundberg, M., Nielsen, L., & Rolving, N. (2020). The effect of graded activity and pain education (GAPE): An early post-surgical rehabilitation programme after lumbar spinal fusion—Study protocol for a randomized controlled trial. Trials, 21(1). https://doi.org/10.1186/s13063-020-04719-y | Focus mainly on pain and not on PAP |
|  | Tenbult, N., Kraal, J., Brouwers, R., Spee, R., Eijsbouts, S., & Kemps, H. (2022). Adherence to a Multidisciplinary Lifestyle Program for Patients With Atrial Fibrillation and Obesity: Feasibility Study. JMIR Formative Research, 6(4). https://doi.org/10.2196/32625 | No PAP-concept |
|  | The Writing Group for the Activity Counseling Trial Research Group, . (2001). Effects of Physical Activity Counseling in Primary Care: The Activity Counseling Trial: A Randomized Controlled Trial. JAMA: The Journal of the American Medical Association, 286(6), 677–687. doi:10.1001/jama.286.6.677 | TIDier (insufficient reporting) |
|  | Thorstensson, C. A., Garellick, G., Rystedt, H., & Dahlberg, L. E. (2015). Better Management of Patients with Osteoarthritis: Development and Nationwide Implementation of an Evidence-Based Supported Osteoarthritis Self-Management Programme. Musculoskeletal care, 13(2), 67–75. https://doi.org/10.1002/msc.1085 | Focus mainly on disease and not on PAP |
|  | Thurston C, Bezuidenhout L, Humphries S, Johansson S, von Koch L, Häger CK, Holmlund L, Sundberg CJ, Garcia-Ptacek S, Kwak L, Nilsson M, English C, Conradsson DM. Mobile health to promote physical activity in people post stroke or transient ischemic attack - study protocol for a feasibility randomised controlled trial. BMC Neurol. 2023 Mar 28;23(1):124. doi: 10.1186/s12883-023-03163-0 | Concept already included |
|  | Tóth-Zsámboki, E., Horváth, Z., Hajtman, L., Leé, S., Pállinger, É., Kuklis, E., Tahy, Á., Fekete, G., Kohut, L., & Kiss, R. G. (2017). Cardiac rehabilitation programme as a non-pharmacological platelet inhibitory tool in acute coronary syndrome survivors. European Journal of Preventive Cardiology, 24(11), 1148–1156. https://doi.org/10.1177/2047487317704937 | No PAP-concept |
|  | Tse, M. M. Y., Vong, S. K. S., & Tang, S. K. (2013). Motivational interviewing and exercise programme for community-dwelling older persons with chronic pain: A randomised controlled study. Journal of Clinical Nursing, 22(13), 1843–1856. https://dx.doi.org/10.1111/j.1365-2702.2012.04317.x | Focus mainly on pain and not on PAP |
|  | Tuvemo Johnson, S., Anens, E., Johansson, A.-C., & Hellstrom, K. (2021). The Otago Exercise Program with or without motivational interviewing for community-dwelling older adults: A 12-month follow-up of a randomized, controlled trial. Journal of Applied Gerontology, 40(3), 289–299. https://dx.doi.org/10.1177/0733464820902652 | No PAP-concept |
|  | Twisk, F. N. M., & Arnoldus, R. J. W. (2012). Graded Exercise Therapy (GET)/Cognitive Behavioural Therapy (CBT) is often counterproductive in Myalgic Encephalomyelitis (ME) and Chronic Fatigue Syndrome (CFS). European Journal of Clinical Investigation, 42(11), 1255–1256. https://doi.org/10.1111/j.1365-2362.2012.02718.x | No PAP-concept |
|  | van Bysterveldt E, Davey S, Douglas N, Liu R, Robertson L, Conroy J, Higgs C, Hale L (2014) A group exercise programme for people at risk from type II diabetes run as a physiotherapy student clinical placement is beneficial: a qualitative study New Zealand Journal of Physiotherapy 42(2): 81-88. | No PAP-concept |
|  | van Engelen, B., & Consortium, O. (2015). Cognitive behaviour therapy plus aerobic exercise training to increase activity in patients with myotonic dystrophy type 1 (DM1) compared to usual care (OPTIMISTIC): Study protocol for randomised controlled trial. TRIALS, 16. https://doi.org/10.1186/s13063-015-0737-7 | Focus mainly on disease and not on PAP |
|  | van Erp, R. M. A., Huijnen, I. P. J., Köke, A. J. A., Abbink, F. E., den Hollander, M., & Smeets, R. J. E. M. (2017). Development and content of the biopsychosocial primary care intervention „Back on Track“ for a subgroup of people with chronic low back pain. Physiotherapy, 103(2), 160–166. https://doi.org/10.1016/j.physio.2016.04.004 | Focus mainly on pain and not on PAP |
|  | van Grootel J, Bor P, Veenhof C, Valkenet K. Development of a goal-directed movement intervention (GOAL) using a movement sensor for hospitalized patients: An intervention mapping approach. Clin Rehabil. 2024 Feb;38(2):251-262. doi: 10.1177/02692155231198173 | TIDier (insufficient reporting) |
|  | van Vulpen, J.K., Siersema, P.D., van Hillegersberg, R., Nieuwen-huijzen, G.A.P., Kouwenhoven, E.A., Groenendijk, R.P.R. et al (2017). Physical ExeRcise Following Esophageal Cancer Treatment (PERFECT) study: design of a randomized controlled trial. BMC Cancer 17(1):552 | TIDier (insufficient reporting) |
|  | van Vulpen JK, Witlox L, Methorst-de Haan AC, Hiensch AE, van Hillegersberg R, Ruurda JP, Nieuwenhuijzen GAP, Kouwenhoven EA, Siersema PD, May AM. Perceived facilitators and barriers by esophageal cancer survivors participating in a post-treatment exercise program. Support Care Cancer. 2023 May 6;31(6):320. doi: 10.1007/s00520-023-07769-5 | No PAP-concept |
|  | Vancampfort, D., De Hert, M., Skjerven, L. H., Gyllensten, A. L., Parker, A., Mulders, N., Nyboe, L., Spencer, F., & Probst, M. (2012). International Organization of Physical Therapy in Mental Health consensus on physical activity within multidisciplinary rehabilitation programmes for minimising cardio-metabolic risk in patients with schizophrenia. Disability and Rehabilitation, 34(1), 1–12. https://doi.org/10.3109/09638288.2011.587090 | No PAP-concept |
|  | Varela, A.J., Melvin, A. (2023).The theatre of depression: a role for physical therapy. Physiother Theory Pract. 2023 Jul 3;39(7):1325-1341. doi: 10.1080/09593985.2022.2041136. Epub 2022 Feb 28. PMID: 35225753. | No PAP-concept |
|  | Veenhof, C., Köke, A. J. A., Dekker, J., Oostendorp, R. A., Bijlsma, J. W. J., Van Tulder, M. W., & Van Den Ende, C. H. M. (2006). Effectiveness of behavioral graded activity in patients with osteoarthritis of the hip and/or knee: A randomized clinical trial. Arthritis Care and Research, 55(6), 925–934. https://doi.org/10.1002/art.22341 | Focus mainly on pain and not on PAP |
|  | Vlaeyen, J. W., de Jong, J., Geilen, M., Heuts, P. H., & van Breukelen, G. (2001). Graded exposure in vivo in the treatment of pain-related fear: a replicated single-case experimental design in four patients with chronic low back pain. Behaviour research and therapy, 39(2), 151–166. https://doi.org/10.1016/s0005-7967(99)00174-6 | Focus mainly on pain and not on PAP |
|  | Vlaeyen, J. W., Haazen, I. W., Schuerman, J. A., Kole-Snijders, A. M., & van Eek, H. (1995). Behavioural rehabilitation of chronic low back pain: comparison of an operant treatment, an operant-cognitive treatment and an operant-respondent treatment. The British journal of clinical psychology, 34(1), 95–118. https://doi.org/10.1111/j.2044-8260.1995.tb01443.x | Focus mainly on pain and not on PAP |
|  | Voet, N. B. M., Bleijenberg, G., Padberg, G. W., van Engelen, B. G. M., & Geurts, A. C. H. (2010). Effect of aerobic exercise training and cognitive behavioural therapy on reduction of chronic fatigue in patients with facioscapulohumeral dystrophy: Protocol of the FACTS-2-FSHD trial. BMC NEUROLOGY, 10. https://doi.org/10.1186/1471-2377-10-56 | No PAP-concept |
|  | Vonk, F., Verhagen, A. P., Twisk, J. W., Koke, A. J. A., Luiten, M. W. C. T., & Koes Bart W.; ORCID: https://orcid.org/0000-0002-0450-9969, B. W. A. I.-K. (2009). Effectiveness of a behaviour graded activity program versus conventional exercise for chronic neck pain patients. European Journal of Pain, 13(5), 533–541. https://dx.doi.org/10.1016/j.ejpain.2008.06.008 | No PAP-concept |
|  | Waite, I., Grant, D., Mayes, J., & Greenwood, S. (2020). Can a brief behavioural change intervention encourage hospital patients with low physical activity levels to engage and initiate a change in physical activity behaviour? PHYSIOTHERAPY, 108, 22–28. https://doi.org/10.1016/j.physio.2020.04.002 | TIDier (insufficient reporting) |
|  | Wang X, David J Hunter, Sarah Robbins, Sarah Capistrano, Vicky Duong, Luciano Melo, Anthony Harris, & Manuela Ferreira. (2021). Participatory health through behavioural engagement and disruptive digital technology for postoperative rehabilitation: Protocol of the PATHway trial. BMJ Open, 11(1), e041328. https://doi.org/10.1136/bmjopen-2020-041328 | Focus mainly on pain and not on PAP |
|  | Waugh, A., Crumlish, N., Kelleher, E., Forde, C., & Broderick, J. (2018). A feasibility study of a physiotherapy-led motivational programme to increase physical activity and improve cardiometabolic risk in people with major mental illness. *General Hospital Psychiatry*, *54*, 37–44. doi:10.1016/j.genhosppsych.2018.03.002 | Focus mainly on disease and not on PAP |
|  | Weber, M. B., Hennink, M. M., & Narayan, K. M. V. (2020). Tailoring lifestyle programmes for diabetes prevention for US South Asians. Family Medicine and Community Health, 8(2). https://doi.org/10.1136/fmch-2019-000295 | Focus mainly on disease and not on PAP |
|  | Weinreich, T., Filz, H.-P., Gresser, U., & Richartz, B. M. (2017). Effectiveness of a four-week diet regimen, exercise and psychological intervention for weight loss. Journal of Clinical and Diagnostic Research, 11(3), LC20–LC24. https://doi.org/10.7860/JCDR/2017/24112.9553 | No PAP-concept |
|  | White, P. D., Sharpe, M. C., Chalder, T., DeCesare, J. C., Walwyn, R., & group, P. trial. (2007). Protocol for the PACE trial: A randomised controlled trial of adaptive pacing, cognitive behaviour therapy, and graded exercise as supplements to standardised specialist medical care versus standardised specialist medical care alone for patients with the chronic fatigue syndrome/myalgic encephalomyelitis or encephalopathy. BMC NEUROLOGY, 7. https://doi.org/10.1186/1471-2377-7-6 | No PAP-concept |
|  | Williams, A., Wiggers, J., O'Brien, K. M., Wolfenden, L., Yoong, S., Campbell, E., Robson, E., McAuley, J., Haskins, R., Kamper, S. J., & Williams, C. M. (2016). A randomised controlled trial of a lifestyle behavioural intervention for patients with low back pain, who are overweight or obese: study protocol. BMC musculoskeletal disorders, 17, 70. https://doi.org/10.1186/s12891-016-0922-1 | Focus mainly on pain and not on PAP |
|  | Wisse, W., Rookhuizen, M. B., de Kruif, M. D., van Rossum, J., Jordans, I., ten Cate, H., van Loon, L. J. C., & Meesters, E. W. (2010). Prescription of physical activity is not sufficient to change sedentary behavior and improve glycemic control in type 2 diabetes patients. Diabetes Research and Clinical Practice, 88(2), e10–e13. https://doi.org/10.1016/j.diabres.2010.01.015 | No PAP-concept |
|  | Wolf S, Seiffer B, Zeibig JM, Welkerling J, Bauer LL, Frei AK, Studnitz T, Rosenstiel S, Fiedler DV, Helmhold F, Ray A, Herzog E, Takano K, Nakagawa T, Kropp S, Franke S, Peters S, El-Kurd N, Zwanzleitner L, Sundmacher L, Ramos-Murguialday A, Hautzinger M, Sudeck G, Ehring T. (2021). Efficacy and cost-effectiveness of a Transdiagnostic group-based exercise intervention: study protocol for a pragmatic multi-site randomized controlled trial. BMC Psychiatry. 2021 Oct 30;21(1):540. doi: 10.1186/s12888-021-03541-3 | Concept already included |
|  | Yamashita, M., Kamiya, K., Hamazaki, N., Uchida, S., Noda, T., Maekawa, E. & Ako, J. (2023). Effects of Acute Phase Intensive Physical Activity (ACTIVE-PA) Monitoring and Education for Cardiac Patients: Pilot Study of a Randomized Controlled Trial. *Journal Of Medical Internet Research*, *25*, e42235. https://doi.org/10.2196/42235 | TIDier (insufficient reporting) |
|  | Zhu, P., & Li, X.-H. (2011). Application of health promotion model in elderly patients with chronic obstructive pulmonary disease. Journal of Shanghai Jiaotong University (Medical Science), 31(6), 713–716. https://doi.org/10.3969/j.issn.1674-8115.2011.06.006 | Wrong language |
|  | Zwerink, M., Van Der Palen, J., Kerstjens, H. A. M., Van Der Valk, P., Brusse-Keizer, M., Zielhuis, G., & Effing, T. (2014). A community-based exercise programme in COPD self-management: Two years follow-up of the COPE-II study. Respiratory Medicine, 108(10), 1481–1490. https://doi.org/10.1016/j.rmed.2014.07.016 | No PAP-concept |

*Note:* PAP = Physical Activity Promotion.

| 1 | Ahmed, R., Bugis, B.A. (2023). Implementation and Importance of Cardiac Rehabilitation for Cardiac Patients in Saudi Arabia: A Systematic Review. Curr Vasc Pharmacol. 2023;21(4):224-233. doi: 10.2174/1570161121666230623115529. PMID: 37357522. | No full text access |
| --- | --- | --- |
| 2 | Arvinen-Barrow, M., & Clement, D. (2018). Persons with exercise injuries. In S. Razon & M. L. Sachs (Eds.), Applied exercise psychology: The challenging journey from motivation to adherence (pp. 339–355). Routledge/Taylor & Francis Group. https://doi.org/10.4324/9780203795422-26 | No full text access |
| 3 | Cabrita, B., Gonçalves, G., Cabrita, A., Pestana, É. (2021). Respiratory Physiotherapy and Pulmonary Rehabilitation. In: Esquinas, A.M. (eds) Pulmonary Function Measurement in Noninvasive Ventilatory Support. Springer, Cham. https://doi.org/10.1007/978-3-030-76197-4_22 | No full text access |
| 4 | Fifolt M, Richardson EV, Barstow EA, Motl RW. (2021). Using Quality Improvement for Refining Program Materials for Exercise Promotion in Comprehensive Multiple Sclerosis Care. J Healthc Qual. 2021 Jul-Aug 01;43(4):249-258. doi: 10.1097/JHQ.0000000000000279. PMID: 34180869. | No full text access |
| 5 | Habibi, H. (2022). Promotion of daily physical activity and exercise for adults with congenital heart disease. In S. F. Flocco, H. Habibi, F. Dellafiore, & C. Sillman (Hrsg.), Guide for advanced nursing care of the adult with congenital heart disease (Vol. 0, Issue 0, pp. 239-252). | No full text access |
| 6 | Hanson H, Wagner M, Monopoli V, Keysor J. (2007). Low back pain in physical therapists: a cultural approach to analysis and intervention. Work. 2007;28(2):145-51. PMID: 17312346. | No full text access |
| 7 | Hays, K.F. (1999). The process of change. Working it out: Using exercise in psychotherapy. - Volume 0, Issue 0, pp. 37-45. https://dx.doi.org/10.1037/10333-004. | No full text access |
| 8 | Hopman-Rock, M., Westhoff, M.H. (2000). The effects of a health educational and exercise program for older adults with osteoarthritis for the hip or knee. J Rheumatol. 2000 Aug;27(8):1947-54. PMID: 10955337. | No full text access |
| 9 | Kress, S., Behrens, M. & Borchert, P. (2023). Cornerstones of successful exercise therapy for type 2 diabetes in practice. Internistische Praxis - Volume 66, Issue 3, pp. 406-414, 10.1007/978-3-030-76197-4_22. | No full text access |
| 10 | Rethorn ZD, Covington JK, Cook CE, Bezner JR. Physical Activity Promotion: Moving From Talking the Talk to Walking the Walk. J Orthop Sports Phys Ther. 2022 May;52(5):236-242. doi: 10.2519/jospt.2022.10859. PMID: 35536250. | No full text access |
| 11 | Segev U., Schlesinger, Z. (1981). Rehabilitation of patients after acute myocardial infarction- an interdisciplinary, family-oriented program. Heart Lung (1981). Sep-Oct;10(5):841-7. PMID: 7024202. | No full text access |
| 12 | Tse, Mimi M Y, RN, PhD; Au, Eva Y M, MSc; Wong, Alex M H, RN, BSN. (2011).Total pain concept: Multisensory stimulation, exercise therapy and coping skill training for community-dwelling older persons with chronic pain  Journal of Pain Management; Hauppauge Bd. 4, Ausg. 4, (2011): 403-416. | No full text access |
