# Supplementary material for: Physical activity promotion in physical therapy, exercise therapy and other movement-based therapies: a scoping review and content analysis of intervention studies and theoretical works
Source: Int J Behav Nutr Phys Act. 2025 Jun 10;22:72. doi: 10.1186/s12966-025-01772-1 (PMC12153217; doi:10.1186/s12966-025-01772-1)
Supplement: Supplementary file 5 — Supplementary Material 5: Additional file 5 contains the codebook used for content analysis. [file 12966_2025_1772_MOESM5_ESM.docx]

**Physical activity promotion in physical therapy, exercise therapy and other movement-based therapies: A scoping review and content analysis of interventional concepts**

**Additional file 5 - Codebook**

Coding scheme

| **Main category** | **Subcategory (level 1)** | **Subcategory (level 2)** |
| --- | --- | --- |
| *Metadata* | | |
| 1. Author, year | / | / |
| 2. Country | / | / |
| 3. Name of concept | / | / |
| *Concept characteristics* | | |
| 1. Brief description of the concept | / | / |
| 2. Target patient population | / | / |
| 3. Target healthcare provider-group | / | / |
| 4. Setting | / | / |
| 5. Theoretical underpinning of the concept | / | / |
| 6. Objective of the concept | / | / |
| 7. Training of the interventionists | / | / |
| 8. Nature of concept | / | / |
| *Core therapeutic processes* | | |
| 1. Assessment | 1.1 Assessment domains | 1.1.1 Physical Activity Behavior (Outcome) |
|  |  | 1.1.2 Previous physical activity experiences |
|  |  | 1.1.3 Other health-related behaviors |
|  |  | 1.1.4 Physical capabilities & skills |
|  |  | 1.1.5 Psychological capabilities & skills |
|  |  | 1.1.6 Additional psychological factors |
|  |  | 1.1.7 Biomedical influencing factors |
|  |  | 1.1.8 Social and physical opportunities |
|  |  | 1.1.9 Others |
|  | 1.2 Purpose of Assessment | 1.2.1 Evaluation of therapy effectiveness |
|  |  | 1.2.2 Instant feedback |
|  |  | 1.2.3 Selection of therapy content |
|  |  | 1.2.4 Development of therapy goals |
| 2. Therapy goals | 2.1 Type of goals (temporal) | 2.1.1 Long-term goals |
|  |  | 2.1.2 Short-term goals |
|  | 2.2 Type of goals (content) | / |
|  | 2.3 Communication style/technique | 2.3.1 Motivational Interviewing |
|  |  | 2.3.2 Shared decision making |
|  | 2.4 Goal setting techniques | 2.4.1 SMART |
| 3. Content, methods and media | 3.1 Behavior change techniques | All 93 BCTs listed by Mitchie et al. 2011 |
|  | 3.2 Physical activity component | 3.2.1 Endurance |
|  |  | 3.2.2 Strength |
|  |  | 3.2.3 Flexibility |
|  |  | 3.2.4 Relaxation |
| 4. Therapy execution | 4.1 Intervention duration | / |
|  | 4.2 Delivery format | / |
|  | 4.3 Number of Sessions | 4.3.1 Number of Sessions (single) |
|  |  | 4.3.2 Number of Sessions (group) |
|  | 4.4 Mode of delivery | / |
| 5. Didactic-methodological principles | 5.1 Active participation in shaping the therapy process by patients | / |
|  | 5.2 Patients' self-responsibility and independence | / |
|  | 5.3 Facilitating positive movement experiences, enjoyment of physical activity | / |
|  | 5.4 Tailoring / Individualization | / |
|  | 5.5 Consideration of interaction & language / Collaborative communication | / |
|  | 5.6 Utilizing group settings | / |

Definition of the categories

**Metadata**

| **AY** | **Author, year** | |
| --- | --- | --- |
| **Description** | | Author and publication year of the concept. |
| **Reference example** | | / |

| **C** | **Country** | |
| --- | --- | --- |
| **Description** | | The country where the intervention was conducted / where the concept was published. |
| **Reference example** | | / |

| **NC** | **Name of Concept** | |
| --- | --- | --- |
| **Description** | | The name of the concept; if no name is specified, the name of the first author is used (e.g., Dean et al.'s concept). |
| **Reference example** | | *„A clinical reasoning model focused on clients’ behaviour change with reference to physiotherapists”* (Elven et al., 2015, p. 1) |

**Concept characteristics**

| **DC** | **Brief description of the concept** | |
| --- | --- | --- |
| **Description** | | A brief summary description of the concept. |
| **Reference example** | | *„a conceptual model to guide physiotherapists’ clinical reasoning*  *focused on clients’ behaviour change.“* (Elven et al., 2015, p. 1) |

| **TPP** | **Target patient population** | |
| --- | --- | --- |
| **Description** | | The patient population addressed in the concept. |
| **Reference example** | | *„Participants were 12 adults with rheumatoid arthritis (RA) diagnosed 6–24 months previously (nine females, three males; mean age 58 years, range 23–79).”* (Cramp et al., 2020, p. 487) |

| **THP** | **Target healthcare provider-group** | |
| --- | --- | --- |
| **Description** | | The group(s) of healthcare providers addressed in the concept. |
| **Reference example** | | *„physiotherapists delivering the intervention“* (Cramp et al., 2020, p. 488) |

| **SET** | **Setting** | |
| --- | --- | --- |
| **Description** | | The setting addressed in the concept. |
| **Reference example** | | *„a primary care setting for people with recently diagnosed RA“* (Cramp et al., 2020, p. 488) |

| **TUC** | **Theoretical underpinning of the concept** | |
| --- | --- | --- |
| **Description** | | Description of the rationale or theories behind the key elements of the concept. |
| **Reference example** | | *“The intervention was based upon a combination of self-determination theory (Ryan & Deci, 2000) and COM-B framework (capability, opportunity, motivation and behaviour) (Michie, van Stralen, & West, 2011) and employed motivational interviewing techniques to promote behaviour change (Miller & Rollnick, 2013).“* (Cramp et al., 2020, p. 489) |

| **OC** | **Objective of the concept (PAP)** | |
| --- | --- | --- |
| **Description** | | The aim of the concept regarding physical activity promotion, or for broader concepts, the overarching goal. |
| **Reference example** | | *„The intervention, informed by a theoretical framework for health behaviour change, aims to support long-term PA engagement to optimise mainte-*  *nance of physical function.“* (Cramp et al., 2020, p. 488) |

| **TI** | **Training of the interventionists** | |
| --- | --- | --- |
| **Description** | | All procedures, activities, and/or processes conducted as part of the intervention/concept for training interventionists. Also, any requirements, prior experiences, and specific considerations relevant to the interventionists. |
| **Reference example** | | *“Band 6 musculoskeletal physiotherapists from primary care received*  *training to deliver PEPA-RA via two half-day workshops. Training was*  *led by a musculoskeletal physiotherapist with experience of delivering*  *similar interventions (RT) and supported by a Rheumatology Clinical*  *Specialist Physiotherapist (MD), a patient research partner (CS) and*  *experts in the delivery of similar PA health behaviour change interven-*  *tions (NW and AH)“* (Cramp et al., 2020, p. 488) |

| **NaC** | **Nature of the concept** | |
| --- | --- | --- |
| **Description** | | The nature of the concept (e.g., intervention study or conceptual framework). |
| **Reference example** | | *„The intervention, informed by a theoretical framework for health behaviour change, aims to support long-term PA engagement to optimise mainte-*  *nance of physical function.“* (Cramp et al., 2020, p. 488) |

**Core therapeutic processes**

| **AS** | **1. Assessment** | |
| --- | --- | --- |
| **Description** | | All procedures, activities, and/or processes used within the intervention/concept for assessing the target patient population (e.g., standardized measurement tools, initial interviews). These include all measurements conducted before, during, and after the intervention. Excluded are assessments used for participant inclusion/exclusion. |
| **Reference example** | | *„Identification of the problem and prioritized activity-related behaviour. Collection of data through interview, observation, physical assessment, and self-monitoring“*  (Elven et al., 2015, p. 9) |

|  | **1.1 Assessment Domains** | |
| --- | --- | --- |
| **Description** | | This category encompasses all aspects related to 'what' is being measured, including the specific areas, categories, and constructs evaluated during an assessment. |
| **Reference example** | | *“The physician’s appointment is followed by two hours of detailed assessment and the evaluation of functional capacity and body composition by an experienced physiotherapist. The tests include a 6-minute walking test (occasionally, a stress-ECG test is done prior to a walking test for patient safety), body composition analysis (bioimpedance, Biospace InBody 770), sit-ups from a chair (with patients over 55 years), squats (with patients 35–55 years), hand-grip strength and one-leg stance. In the case of a specific need, either a medical or psychological or another appointment with the physiotherapist is scheduled.”* (Alanko et al., 2022, p. 6) |

|  | **1.1.1 Physical Activity Behavior (Outcome)** | |
| --- | --- | --- |
| **Description** | | This category includes all measurements related to current movement behavior, encompassing the frequency, intensity, duration, and types of physical activities performed by an individual (e.g. questionnaire or accelerometer/pedometer). This also includes all forms of self-monitoring related to physical activity. |
| **Reference example** | | *“Physical activity will be measured using a Fitbit ZipTM wearable activity tracker. The trackers provide an objective indicator of physical activity behaviour and avoid common sources of error in subjective measurement (e.g. self-report measurement).”*  (Casey et al., 2018, p. 7) |

|  | **1.1.2 Previous physical activity experiences** | |
| --- | --- | --- |
| **Description** | | This category includes all measurements related to the sport or exercise history. |
| **Reference example** | | *“Sport history, […] were identified, using a personalized registration form.”* (Van der Ploeg et al., 2006, p. 224) |

|  | **1.1.3 Other health-related behaviors** | |
| --- | --- | --- |
| **Description** | | This category includes all measurements related to other health-related behaviors (e.g. dietary behavior or smoking status). |
| **Reference example** | | *“[…] patients complete both the lifestyle questionnaire (including questions on […] dietary habits, sleep, alcohol consumption, smoking) […]”*  (Alanko et al., 2022, p. 5) |

|  | **1.1.4 Physical capabilities & skills** | |
| --- | --- | --- |
| **Description** | | This category includes all measurements related to physical capabilities and skills to engage in the activity-related behavior (e.g. strength, mobility or balance). |
| **Reference example** | | *“At 4 to 6 weeks postoperatively, participants’ shoulder strength is assessed […]”*  (Richmond et al., 2018, p. 6) |

|  | **1.1.5 Psychological capabilities & skills** | |
| --- | --- | --- |
| **Description** | | This category includes all measurements related to psychological capability and skills to engage in the activity-related behavior (e.g. cognitions, beliefs, expectations, fear, self-efficacy etc.). |
| **Reference example** | | *“Confidence scale: Participants are asked to rate their confidence to complete the prescribed exercises on a 10-point Likert scale. If a participant has low confidence (defined as < 7 out of 10 in the Health Trainers Manual), then the physiotherapist will explore reasons for this and will problem-solve solutions to improve confidence in ability to exercise.”*  (Richmond et al., 2018, p. 8) |

|  | **1.1.6 Additional psychological factors** | |
| --- | --- | --- |
| **Description** | | This category includes all measurements related to additional psychological factors that are not directly related to the activity-related behavior (e.g. depression, well-being, mindfulness etc.) |
| **Reference example** | | *“Self-reported measures assessed anxiety symptoms (Generalized Anxiety Disorder Questionnaire, GAD-7), depressive symptoms (Patient Health Questionnaire, PHQ-9, and general distress (Distress Thermometer) […]”* (Christie et al., 2021, p. 7368) |

|  | **1.1.7 Biomedical influencing factors** | |
| --- | --- | --- |
| **Description** | | This category includes all measurements related to biomedical deficits, such as disease and tissue pathology, as well as health status indicators such as BMI and overall health metrics. |
| **Reference example** | | *“Laboratory tests (including an ECG) are collected prior to the first visit to assist in the medical evaluation of the patient’s risk factors.”* (Alanko et al., 2022, p. 5) |

|  | **1.1.8 Social and physical opportunities** | |
| --- | --- | --- |
| **Description** | | This category includes both physical and social opportunities, encompassing the environmental and social factors that influence an individual's behavior. |
| **Reference example** | | *“Identifying preferences regarding social context of physical exercise”* (Sudeck & Höhner 2011, p. 157) |

|  | **1.1.9 Others** | |
| --- | --- | --- |
| **Description** | | This category includes all factors that could not be covered by other categories, e.g., quality of life. |
| **Reference example** | | *“patients complete […] the quality of life questionnaire RAND-36”* (Alanko et al., 2022, p. 5) |

|  | **1.2 Purpose of Assessment** | |
| --- | --- | --- |
| **Description** | | This category includes the reasons and objectives behind conducting assessments. |
| **Reference example** | | *“Self-reported PA was measured at 1-, 2-, 3-, and 6-month follow-ups to examine whether intervention effects were maintained.”* (Ma et al., 2019, p. 1120) |

|  | **1.2.1 Evaluation of therapy effectiveness** | |
| --- | --- | --- |
| **Description** | | This category encodes instances where the assessment was utilized to evaluate the effectiveness of the therapy. |
| **Reference example** | | *“Self-reported PA was measured at 1-, 2-, 3-, and 6-month follow-ups to examine whether intervention effects were maintained.”* (Ma et al., 2019, p. 1120) |

|  | **1.2.2 Instant feedback** | |
| --- | --- | --- |
| **Description** | | This category encodes instances where the assessment was utilized to provide instant feedback for the participants. |
| **Reference example** | | *“Exercise diary: Participants will complete an exercise diary for review at each appointment. This diary provides immediate feedback and self-monitoring, and serves as a reminder to complete their exercises.”* (Richmond et al., 2018, p. 8) |

|  | **1.2.3 Selection of therapy content** | |
| --- | --- | --- |
| **Description** | | This category encodes instances where the assessment was utilized to select the therapy content. |
| **Reference example** | | *“The barrier and task self- efficacy score outcome measure, was used as the basis for the tailoring of behaviour change techniques”* (Johnson et al., 2020, p. 3) |

|  | **1.2.4 Development of therapy goals** | |
| --- | --- | --- |
| **Description** | | This category encodes instances where the assessment was utilized to inform the development of therapy goals. |
| **Reference example** | | *“Furthermore, for each subject the stage of physical activity change was assessed using a questionnaire, after which subjects received a stage-specific folder with assignments and tips to become more physically active. This included for example goal setting, rewarding, obtaining social support, and relapse prevention. Furthermore, attention was paid to possible solutions for barriers and to new physical activity possibilities.”* (Van der Ploeg et al., 2006, p. 225) |

| **TP** | **2. Therapy goals** | |
| --- | --- | --- |
| **Description** | | All procedures, activities, and/or processes used within the intervention/concept for formulating specific goals (e.g., based on assessments), such as shared decision-making, RUMBA, SMART, etc. |
| **Reference example** | | *“Introduce goal setting*  *• What is it and how might it help?*  *• Set one SMART goal”*  (Saxton et al., 2013, p. 209) |

|  | **2.1 Type of goals (temporal)** | |
| --- | --- | --- |
| **Description** | | This category includes all temporal information related to therapy goals (e.g. long-term goals). |
| **Reference example** | | *“Moreover, a long-term exercise goal is set and potential future barriers, facilitators and strategies can be brought up and discussed in order to facilitate future adherence to exercise.”* (Borg et al., 2017, p. 4) |

|  | **2.1.1 Long-term goals** | |
| --- | --- | --- |
| **Description** | | This category was applied when long-term goals were reported (sometimes, the concepts referred to goals as 'long-term,' but this category also included goals that extend beyond the therapy period or last longer than 8 weeks). |
| **Reference example** | | *“Moreover, a long-term exercise goal is set and potential future barriers, facilitators and strategies can be brought up and discussed in order to facilitate future adherence to exercise.”* (Borg et al., 2017, p. 4) |

|  | **2.1.2 Short-term goals** | |
| --- | --- | --- |
| **Description** | | This category was applied when short-term goals were reported (sometimes, the concepts labeled goals as 'short-term,' but this category also encompassed goals that were confined to the therapy period or lasted no longer than 8 weeks). |
| **Reference example** | | *“Coaching sessions began with a review of patients’ exercise diaries. Patients received feedback on their progress, and worked with the nurse to set short-term, realistic PA goals and action plans for the coming week (i.e. what PA, when, for how long).”* (Knittle et al., 2015, p. 233) |

|  | **2.2 Type of goals (content)** | |
| --- | --- | --- |
| **Description** | | This category includes all content related information related to therapy goals (explicit examples of goals e.g. walking for 2 hours a week). |
| **Reference example** | | *“At a minimum, the interventionist suggested achieving the international SCI exercise guidelines to improve fitness (at least 20 min of moderate– vigorous aerobic activity twice/week and strength training twice/week). For those already exceeding the fitness guidelines, the international SCI exercise guidelines to improve cardio-metabolic health were set as the goal (at least 30 min of moderate to vigorous aerobic activity three times/week) plus strength training twice/week.”* (Ma et al., 2019, p. 1121) |

|  | **2.3 Communication style/technique** | |
| --- | --- | --- |
| **Description** | | This category covers all information about how communication is conducted with the patient (e.g. motivational interviewing). |
| **Reference example** | | *“Motivational interviewing is used to find and deal with the barriers to individuals becoming physically active, but also to find out what personal goals a patient wants to achieve.”*  (De Vries et al., 2013, p. 3) |

|  | **2.3.1 Motivational Interviewing** | |
| --- | --- | --- |
| **Description** | | This category was applied when motivational interviewing was explicitly mentioned. |
| **Reference example** | | *“Motivational interviewing is used to find and deal with the barriers to individuals becoming physically active, but also to find out what personal goals a patient wants to achieve.”*  (De Vries et al., 2013, p. 3) |

|  | **2.3.2 Shared decision making** | |
| --- | --- | --- |
| **Description** | | This category was applied when shared decision making was explicitly mentioned or when the principles surrounding it were described (e.g. collaboration with the patient). |
| **Reference example** | | *“In collaboration with the principal investigator, participants were asked to implement one goal per week to reduce SB relating to any of the three target behaviours, so that by the sixth week of the intervention participants would have integrated six goals into daily life to reduce SB (e.g., Week 1: ‘Stand up and move during the TV advertisement breaks’, Week 2: ‘Stand up to talk on the phone instead of sitting down’).”* (Cheng et al., 2022, Supplemental material) |

|  | **2.4 Goal setting techniques** | |
| --- | --- | --- |
| **Description** | | This category covers all information about how goal setting is conducted with the patient (e.g. SMART). |
| **Reference example** | | *“The goal should be specific, measurable, activity-related, realistic and time-specific (SMART).”* (Elvén et al., 2015, p. 10) |

|  | **2.4.1 SMART** | |
| --- | --- | --- |
| **Description** | | This category was applied when SMART goal setting was explicitly mentioned. |
| **Reference example** | | *“The goal should be specific, measurable, activity-related, realistic and time-specific (SMART).”* (Elvén et al., 2015, p. 10) |

| **CMM** | **3. Content, methods, media** | |
| --- | --- | --- |
| **Description** | | All informational or physical materials used in the intervention/concept during treatment. All procedures, activities, and/or processes used in the intervention/concept for patient treatment. These can include, for example, conversation techniques (e.g., motivational interviewing), behavior change techniques (BCTs), or linking theory and practice. |
| **Reference example** | | *“Intervention elements included gaining commitment, identifying valued outcomes, setting goals, making choices, action planning, self-monitoring, identifying opportunities for physical activity at home, in the neighbourhood, and in the community, problem solving to overcome barriers, feedback and encouragement, symptoms and intensity management, and links to medical care.”*  (Reid et al., 2012, p. 162) |

|  | **3.1 Behavior Change Techniques (BCTs)** | |
| --- | --- | --- |
| **Description** | | A Behavior Change Technique (BCT) is “an observable, replicable, and irreducible component of an intervention designed to alter or redirect causal processes that regulate behavior; that is, a technique is proposed to be an ‘active ingredient’ (e.g. feedback, self-monitoring, and reinforcement).” (Mitchie et al., 2013, p. 82) |
| **Reference example** | | *Problem Solving: “Prompting the patients to identify barriers to exercise (self-management sessions)”*  (Bricca et al., 2022, p. 9) |

*All Behavior Change Techniques are described according to Mitchie et al. 2013.*

|  | **3.1 - 1.1 Goal setting (behavior)** | |
| --- | --- | --- |
| **Description** | | Set or agree on a goal defined in terms of the behavior to be achieved |
| **Reference example** | | *“By the end of the visit, goals are set and recorded. The goals, which are tailored to each patient individually, include the amount of increase in activity/exercise, possible changes in diet and decrease in substance use, screen time, and so forth.”*  *(*Alanko et al., 2022, p. 5*)* |

|  | **3.1 - 1.2 Problem solving** | |
| --- | --- | --- |
| **Description** | | Analyse , or prompt the person to analyse, factors influencing the behavior and generate or select strategies that include overcoming barriers and/or increasing facilitators (includes ‘Relapse Prevention’ and ‘Coping Planning’). |
| **Reference example** | | *“Prompting the patients to identify barriers to exercise (self-management sessions)*  (Bricca et al., 2022, p. 9) |

|  | **3.1 - 1.3 Goal setting (outcome)** | |
| --- | --- | --- |
| **Description** | | Set or agree on a goal defined in terms of a positive outcome of wanted behavior. |
| **Reference example** | | *“Enablement – Goal setting outcome”*  (Johnson et al., 2020, p. 10)*.* |

|  | **3.1 - 1.4 Action planning** | |
| --- | --- | --- |
| **Description** | | Prompt detailed planning of performance of the behavior (must include at least one of context, frequency, duration and intensity). Context may be environmental (physical or social) or internal (physical, emotional or cognitive) (includes ‘Implementation Intentions’). |
| **Reference example** | | *“The action plan for each goal was documented in a workbook.”*  (Cheng et al., 2022, p. 249) |

|  | **3.1 - 1.5 Review behavior goal(s)** | |
| --- | --- | --- |
| **Description** | | Review behavior goal(s) jointly with the person and consider modifying goal(s) or behavior change strategy in light of achievement. This may lead to re-setting the same goal, a small change in that goal or setting a new goal instead of (or in addition to) the first, or no change. |
| **Reference example** | | *“Secure/assure positive experiences of consequences Discussion of the satisfaction with the achieved consequences of PA based on the beforehand formulated realistic objectives and expectations”*  (Geidl et al., 2014, p. 2095) |

|  | **3.1 - 1.6 Discrepancy between current behavior and goal** | |
| --- | --- | --- |
| **Description** | | Draw attention to discrepancies between a person’s current behavior (in terms of the form, frequency, duration, or intensity of that behavior) and the person’s previously set outcome goals, behavioral goals or action plans (goes beyond self- monitoring of behavior). |
| **Reference example** | | *“Comparison of the self-formulated goals with the actual behaviour or the training improvements.”*  (Geidl et al., 2014, p. 2095) |

|  | **3.1 - 1.7 Review outcome goal(s)** | |
| --- | --- | --- |
| **Description** | | Review outcome goal(s) jointly with the person and consider modifying goal(s) in light of achievement. This may lead to re- setting the same goal, a small change in that goal or setting a new goal instead of, or in addition to the first. |
| **Reference example** | | *“The progress review session/s will be timed around the short-term goals (reviewed within less than three months) identified by the stroke survivors, e.g. if a goal is set for three weeks’ time, the review session will be de-livered three weeks from when the goal was set. The re-view will focus on progress towards outcomes and behavioural goals.“*  (Moore et al., 2020, p. 5) |

|  | **3.1 - 1.8 Behavioral contract** | |
| --- | --- | --- |
| **Description** | | Create a written specification of the behavior to be performed, agreed on by the person, and witnessed by another. |
| **Reference example** | | *“Concrete formulation and written confirmation of personal, short-term, achievable but appropriate exercise goals”*  (Geidl et al. 2014, p. 2095) |

|  | **3.1 - 1.9 Commitment** | |
| --- | --- | --- |
| **Description** | | Ask the person to affirm or reaffirm statements indicating commitment to change the behavior. |
| **Reference example** | | *“Committing to action that improves and enriches one’s life”*  (Casey et al., 2018, p. 5) |

|  | **3.1 - 2.1 Monitoring of behavior by others without feedback** | |
| --- | --- | --- |
| **Description** | | Observe or record behavior with the person’s knowledge as part of a behavior change strategy. |
| **Reference example** | | *“Environment: Specialized therapists tailor personal exercise programs, provide individual counselling and provide (follow-up) monitoring of exercise, outcomes and coping responses.*  (Hilberdink et al., 2020, p. 956) |

|  | **3.1 - 2.2 Feedback on behavior** | |
| --- | --- | --- |
| **Description** | | Monitor and provide informative or evaluative feedback on performance of the behavior (e.g. form, frequency, duration, intensity). |
| **Reference example** | | *“During control visits, patients’ progress is evaluated. Positive feedback is given even for patients with minor advances and possible problems are assessed.*  (Alanko et al., 2022, p. 6) |

|  | **3.1 - 2.3 Self-monitoring of behavior** | |
| --- | --- | --- |
| **Description** | | Establish a method for the person to monitor and record their behavior(s) as part of a behavior change strategy. |
| **Reference example** | | *“The participants recorded on the mail-back cards their goals and the frequency and duration of exercise for each week of the previous month. As part of the ongoing self-monitoring portion of the GMCB intervention, participants recorded their activity throughout the month and then transferred this information onto the mail-back card. In addition, they recorded their total steps for each week and whether they planned to maintain, decrease, or increase their activity for the next month.*  (Focht et al., 2004, p. 56) |

|  | **3.1 - 2.4 Self-monitoring of outcome(s) of behavior** | |
| --- | --- | --- |
| **Description** | | Establish a method for the person to monitor and record the outcome(s) of their behavior as part of a behavior change strategy. |
| **Reference example** | | *“2.4 Self-monitoring of outcome(s) of behaviour Ask the patients to record the exercise sessions and exertion (exercise therapy sessions)”*  (Bricca2022, p. 9) |

|  | **3.1 - 2.6 Biofeedback** | |
| --- | --- | --- |
| **Description** | | Provide feedback about the body (e.g. physiological or biochemical state) using an external monitoring device as part of a behavior change strategy. |
| **Reference example** | | *“After the stretching exercises, participants self-monitored and recorded a final cool-down heart rate”*  (Focht et al., 2004, p. 55) |

|  | **3.1 - 2.7 Feedback on outcome(s) of behavior** | |
| --- | --- | --- |
| **Description** | | Monitor and provide feedback on the outcome of performance of the behavior. |
| **Reference example** | | *“2.7 Feedback on outcome(s) of behavior “*  (Ma et al., 2019, Supplementary File 1) |

|  | **3.1 - 3.1 Social support (unspecified)** | |
| --- | --- | --- |
| **Description** | | Advise on, arrange or provide social support (e.g. from friends, relatives, colleagues,’ buddies’ or staff) or non- contingent praise or reward for performance of the behavior. It includes encouragement and counselling, but only when it is directed at the behavior. |
| **Reference example** | | *“Identifying preferences regarding social context of physical exercise”*  (Sudeck & Höner, 2011, p. 157) |

|  | **3.1 - 3.2 Social support (practical)** | |
| --- | --- | --- |
| **Description** | | Advise on, arrange, or provide practical help (e.g. from friends, relatives, colleagues, ‘buddies’ or staff) for performance of the behavior. |
| **Reference example** | | *“Planning concrete situations for physical exercise (action plans on where, when, and with whom)”*  (Sudeck & Höner, 2011, p. 157) |

|  | **3.1 - 3.3 Social support (emotional)** | |
| --- | --- | --- |
| **Description** | | Advise on, arrange, or provide emotional social support (e.g. from friends, relatives, colleagues, ‘buddies’ or staff) for performance of the behavior. |
| **Reference example** | | *“Define commitment and motivation using the motivational interviewing ‘rulers’”*  (Rethorn et al., 2021, p. 5) |

|  | **3.1 - 4.1 Instructing on how to perform a behavior** | |
| --- | --- | --- |
| **Description** | | Advise or agree on how to perform the behavior (includes ‘Skills training’). |
| **Reference example** | | *“each GMV, which combined specific medical advice, medication management, open discussion of patient concerns, a targeted educational component to guide participants’ self-management or ‘patient activation’ around PA, as well as a PA component.*  (Adams et al., 2015, p. 2-3) |

|  | **3.1 - 4.2 Information about antecedents** | |
| --- | --- | --- |
| **Description** | | Provide information about antecedents (e.g. social and environmental situations and events, emotions, cognitions) that reliably predict performance of the behaviour. |
| **Reference example** | | *“discussion about antecedents and consequences of the behaviour”*  (Elven et al., 2015, p. 10) |

|  | **3.1 - 4.3 Re-attribution** | |
| --- | --- | --- |
| **Description** | | Elicit perceived causes of behavior and suggest alternative explanations (e.g. external or internal and stable or unstable). |
| **Reference example** | | *“re-attribution”*  (O’Dwyer et al., 2017, p. 31) |

|  | **3.1 - 5.1 Information about health consequences** | |
| --- | --- | --- |
| **Description** | | Provide information (e.g. written, verbal, visual) about health consequences of performing the behavior. |
| **Reference example** | | *“During the PA component, participants went for a group walk on alternate weeks, emphasising that 150 min weekly of moderate activity (eg, brisk walking) was all that is necessary to obtain the medical benefits offered by regular PA.”*  (Adams et al., 2015, p. 3) |

|  | **3.1 - 5.2 Salience of consequences** | |
| --- | --- | --- |
| **Description** | | Use methods specifically designed to emphasise the consequences of performing the behaviour with the aim of making them more memorable (goes beyond informing about consequences). |
| **Reference example** | | *“Salience of consequences”*  (Moore et al., 2020, p.8) |

|  | **3.1 – 5.3 Information about social and environmental consequences** | |
| --- | --- | --- |
| **Description** | | Provide information (e.g. written, verbal, visual) about social and environmental consequences of performing the behavior. |
| **Reference example** | | “Information about social and environmental consequences”  (Moore et al., 2020, p.8) |

|  | **3.1 - 5.4 Monitoring of emotional consequences** | |
| --- | --- | --- |
| **Description** | | Prompt assessment of feelings after attempts at performing the behavior. |
| **Reference example** | | *“monitoring of emotional consequences”*  (O’Dwyer et al., 2017, p. 31) |

|  | **3.1 - 5.5 Anticipated Regret** | |
| --- | --- | --- |
| **Description** | | Induce or raise awareness of expectations of future regret about performance of the unwanted behavior. |
| **Reference example** | | *“anticipated regret”*  (O’Dwyer et al., 2017, p. 31) |

|  | **3.1 - 5.6 Information about emotional consequences** | |
| --- | --- | --- |
| **Description** | | Provide information (e.g. written, verbal, visual) about emotional consequences of performing the behavior. |
| **Reference example** | | *“Information about emotional consequences”*  (Moore et al., 2020, p. 8) |

|  | **3.1 - 6.1 Demonstration of the behavior** | |
| --- | --- | --- |
| **Description** | | Provide an observable sample of the performance of the behaviour, directly in person or indirectly e.g. via film, pictures, for the person to aspire to or mitate (includes ‘Modelling’). |
| **Reference example** | | *“On alternate weeks, participants stayed in the gym and were taught techniques and exercises that they could employ in their everyday lives to become more physically active without having to attend special classes, set aside time, or make concerted efforts to ‘exercise’ (eg, in one session, called ‘commercial exercises’, participants were given a series of brief (<5 min) upper and lower body activities they could do in their home during a television commercial in lieu of sitting still, eating a snack, etc).”*  (Adams et al., 2015, p. 3) |

|  | **3.1 - 6.2 Social comparison** | |
| --- | --- | --- |
| **Description** | | Draw attention to others’ performance to allow comparison with the person’s own performance. |
| **Reference example** | | *“Social comparison”*  (Willett et al., 2021, p. 9) |

|  | **3.1 - 6.3 Information about others’ approval** | |
| --- | --- | --- |
| **Description** | | Provide information about what other people think about the behavior. The information clarifies whether others will like, approve or disapprove of what the person is doing or will do. |
| **Reference example** | | *“Information about others’ approval”*  (Willett et al., 2021, p. 9) |

|  | **3.1 - 7.1 Prompts/cues** | |
| --- | --- | --- |
| **Description** | | Introduce or define environmental or social stimulus with the purpose of prompting or cueing the behavior. The prompt or cue would normally occur at the time or place of performance. |
| **Reference example** | | *“If yes well done! If not, why not? What can we do to help change this? Cues for action”*  (Saxton et al., 2013, S. 209) |

|  | **3.1 - 7.7 Exposure** | |
| --- | --- | --- |
| **Description** | | Provide systematic confrontation with a feared stimulus to reduce the response to a later encounter. |
| **Reference example** | | *“Reduce fear-avoidance through graded activity or exposure in vivo”*  (Elven et al. 2015, S. 10) |

|  | **3.1 - 8.1 Behavioral practice/rehearsal** | |
| --- | --- | --- |
| **Description** | | Prompt practice or rehearsal of the performance of the behavior one or more times in a context or at a time when the performance may not be necessary, in order to increase habit and skill. |
| **Reference example** | | *“The exercise sessions will take place in either a pool or a gym setting (four sessions of each). The aquatic sessions will include a warm up, gentle aerobic exercise, buoyancy-assisted and resisted movements, and informal ball games. The gym programme will feature a combination of gentle aerobic exercise, stretches and strengthening exercises. will be provided.* (Casey et al., 2018, p. 5) |

|  | **3.1 - 8.2 Behavior substitution** | |
| --- | --- | --- |
| **Description** | | Prompt substitution of the unwanted behavior with a wanted or neutral behavior. |
| **Reference example** | | *“Behaviour substitution”*  (O’Dwyer et al., 2017, p. 31) |

|  | **3.1 - 8.3 Habit formation** | |
| --- | --- | --- |
| **Description** | | Prompt rehearsal and repetition of the behavior in the same context repeatedly so that the context elicits the behavior. |
| **Reference example** | | *“habit formation”*  (O’Dwyer et al., 2017, p. 31) |

|  | **3.1 - 8.4 Habit reversal** | |
| --- | --- | --- |
| **Description** | | Prompt rehearsal and repetition of an alternative behavior to replace an unwanted habitual behavior. |
| **Reference example** | | *“habit reversal”*  (O’Dwyer et al., 2017, p. 31) |

|  | **3.1 - 8.6 Generalisation of a target behavior** | |
| --- | --- | --- |
| **Description** | | Advise to perform the wanted behaviour, which is already performed in a particular situation, in another situation. |
| **Reference example** | | *“Participants will be encouraged to carry out their exercises at home*  *or in their local pool.”*  (Casey et al., 2018, p. 6) |

|  | **3.1 - 8.7 Graded taks** | |
| --- | --- | --- |
| **Description** | | Set easy-to-perform tasks, making them increasingly difficult, but achievable, until behavior is performed. |
| **Reference example** | | *The programmes will be progressed and modified for each individual as deemed appropriate by the physiotherapist.*  (Casey et al., 2018, p. 6) |

|  | **3.1 - 9.1 Credible source** | |
| --- | --- | --- |
| **Description** | | Present verbal or visual communication from a credible source in favour of or against the behavior. |
| **Reference example** | | *“Credible source”*  (Willett et al., 2021, p. 9) |

|  | **3.1 - 9.2 Pros and cons** | |
| --- | --- | --- |
| **Description** | | Advise the person to identify and compare reasons for wanting (pros) and not wanting to (cons) change the behavior (includes ‘Decisional balance’). |
| **Reference example** | | *Do you know? Benefits of exercise. Importance of healthy eating. How are you managing ­– review pros and cons of exercise?*  (Daley et al., 2004, p. 694) |

|  | **3.1 - 9.3 Comparative imagining of future outcomes** | |
| --- | --- | --- |
| **Description** | | Prompt or advise the imagining and comparing of future outcomes of changed versus unchanged behaviour. |
| **Reference example** | | *“Comparative imagining of future outcomes”*  (O’Dwyer et al., 2017, p. 31) |

|  | **3.1 - 10.2 Material reward (behavior)** | |
| --- | --- | --- |
| **Description** | | Arrange for the delivery of money, vouchers or other valued objects if and only if there has been effort and/or progress in performing the behavior (includes ‘Positive reinforcement’). |
| **Reference example** | | *“Rewards (one of: Material, non-specific, social, self-reward,*  *or outcome).”*  (Willett et al., 2021, p. 9) |

|  | **3.1 - 10.3 Non-specific reward** | |
| --- | --- | --- |
| **Description** | | Arrange delivery of a reward if and only if there has been effort and/or progress in performing the behavior (includes ‘Positive reinforcement’). |
| **Reference example** | | *“Rewards (one of: Material, non-specific, social, self-reward,*  *or outcome).”*  (Willett et al., 2021, p. 9) |

|  | **3.1 - 10.4 Social reward** | |
| --- | --- | --- |
| **Description** | | Arrange verbal or non-verbal reward if and only if there has been effort and/or progress in performing the behavior (includes ‘Positive reinforcement’). |
| **Reference example** | | *“Rewards (one of: Material, non-specific, social, self-reward,*  *or outcome).”*  (Willett et al., 2021, p. 9) |

|  | **3.1 - 10.9 Self-reward** | |
| --- | --- | --- |
| **Description** | | Prompt self-praise or self-reward if and only if there has been effort and/or progress in performing the behavior. |
| **Reference example** | | *“Rewards (one of: Material, non-specific, social, self-reward,*  *or outcome).”*  (Willett et al., 2021, p. 9) |

|  | **3.1 - 10.10 Reward (outcome)** | |
| --- | --- | --- |
| **Description** | | Arrange for the delivery of a reward if and only if there has been effort and/or progress in achieving the behavioral outcome (includes ‘Positive reinforcement’). |
| **Reference example** | | *“Rewards (one of: Material, non-specific, social, self-reward,*  *or outcome).”*  (Willett et al., 2021, p. 9) |

|  | **3.1 - 11.1 Pharmacological support** | |
| --- | --- | --- |
| **Description** | | Provide, or encourage the use of or adherence to, drugs to facilitate behavior change. |
| **Reference example** | | *“Pharmacological support”*  (Willett et al., 2021, p. 9) |

|  | **3.1 - 11.2 Reduce negative emotions** | |
| --- | --- | --- |
| **Description** | | Advise on ways of reducing negative emotions to facilitate performance of the behavior (includes ‘Stress Management’). |
| **Reference example** | | *“Enable informed beliefs Improve self-efficacy through mastery experi-ences, improving physical and emotional states”*  (Elven et al., 2015, p. 10) |

|  | **3.1 - 12.1 Restructuring the physical environment** | |
| --- | --- | --- |
| **Description** | | Change, or advise to change the physical environment in order to facilitate performance of the wanted behavior or create barriers to the unwanted behavior (other than prompts/cues, rewards and punishments). |
| **Reference example** | | *“Modify contextual factors Social support and restructuring of the physical environment enabling the activity-related behaviour.”*  (Elven et al., 2015, p. 11) |

|  | **3.1 - 12.2 Restructuring the social environment** | |
| --- | --- | --- |
| **Description** | | Change, or advise to change the social environment in order to facilitate performance of the wanted behavior or create barriers to the unwanted behavior (other than prompts/cues, rewards and punishments). |
| **Reference example** | | *“Restructuring the social environment”*  (Ma et al., 2019, p. 1140) |

|  | **3.1 - 12.4 Distraction** | |
| --- | --- | --- |
| **Description** | | Advise or arrange to use an alternative focus for attention to avoid triggers for unwanted behaviour. |
| **Reference example** | | *“Distraction”*  (Ma et al., 2019, p. 1140) |

|  | **3.1 - 12.5 Adding objects to the environment** | |
| --- | --- | --- |
| **Description** | | Add objects to the environment in order to facilitate performance of the behavior. |
| **Reference example** | | *“Adding objects to the environment”.*  (Ma et al., 2019, p. 1140) |

|  | **3.1 – 12.6 Body changes** | |
| --- | --- | --- |
| **Description** | | Alter body structure, functioning or support directly to facilitate behavior change. |
| **Reference example** | | *During the PA component, participants went for a group walk on alternate weeks, emphasising that 150 min weekly of moderate activity (eg, brisk walking) was all that is necessary to obtain the medical benefits offered by regular PA.”*  (Adams et al., 2015, p. 3) |

|  | **3.1 - 13.1 Identification of self as a role model** | |
| --- | --- | --- |
| **Description** | | Inform that one's own behavior may be an example to others. |
| **Reference example** | | *“Identificatin of self as a role model.”* (Ma et al., 2019, p. 1140) |

|  | **3.1 - 13.2 Framing/reframing** | |
| --- | --- | --- |
| **Description** | | Suggest the deliberate adoption of a perspective or new perspective on behavior (e.g. its purpose) in order to change cognitions or emotions about performing the behavior (includes ‘Cognitive structuring’). |
| **Reference example** | | *“Acceptance; An ongoing willingness to experience difficult thoughts, feelings, emotion, and/or bodily sensations, without defense in the service of personally chosen values Cognitive defusion: The ability to step back, watch, and observe thoughts without being unnecessarily caught up, dominated, or engulfed by them Looking at thoughts with an attitude of curiosity rather than reactivity Self as context. The “observing” self: free from labels and judgments.”*  (Tatta et al., 2022 , p. 3) |

|  | **3.1 - 13.3 Incompatible beliefs** | |
| --- | --- | --- |
| **Description** | | Draw attention to discrepancies between current or past behavior and self-image, in order to create discomfort (includes ‘Cognitive dissonance’). |
| **Reference example** | | *“Identity: Incompatible beliefs.”*  (O’Dwyer et al., 2017, p. 31) |

|  | **3.1 - 13.4 Valued self-identity** | |
| --- | --- | --- |
| **Description** | | Advise the person to write or complete rating scales about a cherished value or personal strength as a means of affirming the person’s identity as part of a behavior change strategy (includes ‘Self- affirmation’). |
| **Reference example** | | *“relating intended behaviour to values”*  (Elsman et al., 2014, p. 13) |

|  | **3.1 - 15.1 Verbal persuasion about capability** | |
| --- | --- | --- |
| **Description** | | Tell the person that they can successfully perform the wanted behavior, arguing against self-doubts and asserting that they can and will succeed. |
| **Reference example** | | *“The facilitators are instructed to persuade the patients that they*  *can perform the exercises asserting that they can and will succeed (exercise therapy sessions).”*  (Bricca et al. 2022, p. 9) |

|  | **3.1 - 15.3 Focus on past success** | |
| --- | --- | --- |
| **Description** | | Advise to think about or list previous successes in performing the behavior (or parts of it). |
| **Reference example** | | *“Reflection about positive experiences/effects with/of exercise.”*  (Wolf et al., 2021, p. 9) |

|  | **3.1 - 16.3 Vicarious consequences** | |
| --- | --- | --- |
| **Description** | | Prompt observation of the consequences (including rewards and punishments) for others when they perform the behavior. |
| **Reference example** | | *“Vicarious consequences”*  (Ma et al., 2019, p. 1140) |

|  | **3.2 Physical activity component** | |
| --- | --- | --- |
| **Description** | | Any descriptions of intervention components that include physical activity or exercises. |
| **Reference example** | | *“All patients attended bicycle ergometer trainings (20 to 30 minutes) four to five times a week and medical training therapy (30 minutes) five to six times a week.”* (Sudeck & Höhner 2011, p. 160) |

| **TE** | **4. Therapy execution** | |
| --- | --- | --- |
| **Description** | | The specific application of the content, methods, and media in terms of how: 'How is the intervention/concept applied?' (e.g., personal contact, individual or group intervention, group size, etc.) Where: 'Where is the intervention/concept applied?' (e.g., specific characteristics of the facilities, necessary infrastructure) When and how much: 'How often and over what period is the intervention/concept applied?' (e.g., number, frequency, and duration of sessions, training units, etc.). |
| **Reference example** | | *“Participants in the intervention group will receive four physical activity sessions with a trained physiotherapist delivered over 3 months, supported by a handbook and pedometer, in addition to usual care. Participants will be provided with a handbook prior to session 1. “* (Ryan et al., 2017, p. 4) |

|  | **4.1 Intervention duration** | |
| --- | --- | --- |
| **Description** | | The duration of the intervention. |
| **Reference example** | | *“Participants in the intervention group will receive four physical activity sessions with a trained physiotherapist delivered over 3 months […] “* (Ryan et al., 2017, p. 4) |

|  | **4.2 Delivery format** | |
| --- | --- | --- |
| **Description** | | Format of delivery (e.g., one-to-one, group or combined). |
| **Reference example** | | *“The intervention consisted of four group sessions and a one-to-one session […] “* (Cramp et al., 2020, p. 489) |

|  | **4.3 Number of Sessions** | |
| --- | --- | --- |
| **Description** | | Number of Sessions. |
| **Reference example** | | *“In Coach2Move, three intervention profiles with a predefined number of sessions can be chosen. Patients are categorized into one of these three profiles based on the complexity of their problems and their potential for improvement. The first profile deals with patients who do not need physiotherapy intervention on the level of body function or structure, but who just need coaching and advice (≤4 sessions).”* (De Vries et al., 2013, p. 4) |

|  | **4.3.1 Number of Sessions (single)** | |
| --- | --- | --- |
| **Description** | | Number of one-to-one Sessions. |
| **Reference example** | | *“The intervention consisted of four group sessions and a one-to-one session […] “* (Cramp et al., 2020, p. 489) |

|  | **4.3.1 Number of Sessions (group)** | |
| --- | --- | --- |
| **Description** | | Number of group Sessions. |
| **Reference example** | | *“The intervention consisted of four group sessions and a one-to-one session […] “* (Cramp et al., 2020, p. 489) |

|  | **4.4 Mode of delivery** | |
| --- | --- | --- |
| **Description** | | Mode of delivery (e.g., face-to-face). |
| **Reference example** | | *“Coaching sessions were delivered either in person at the research facility, over Skype, or - when these modes were not possible - over the phone.”* (Ma et al., 2019, p. 1120) |

|  | **5. Didactic-methodological principles** | |
| --- | --- | --- |
| **Description** | | Underlying didactic-methodological principles that informed the intervention's implementation. |
| **Reference example** | | *“Action plans were patient-centred and individuals supported to undertake PA of their choice to promote intrinsic motivation.”* (Cramp et al., 2020, p. 489) |

|  | **5.1 Active participation in shaping the therapy process by patients** | |
| --- | --- | --- |
| **Description** | | This category was applied when the patient was involved into shaping the therapy process together with the therapist (e.g. shared decision making). |
| **Reference example** | | *“Collaborate with the patient or client to share examination findings and determine their readiness to change with respect to the patient’s or client’s desires and views.”* (Lein et al., 2017, p. 1175) |

|  | **5.2 Patients' self-responsibility and independence** | |
| --- | --- | --- |
| **Description** | | This category was applied when the patient's responsibility for their own behavior was emphasized, or when the patient's dependence on the therapist was addressed in any manner. |
| **Reference example** | | *“During the first 3-month period, there was a process of phased increase in personal responsibility for exercise among participants in conjunction with a phased decrease in staff, group, and clinic dependency.”* (Focht et al., 2004, p. 55) |

|  | **5.3 Facilitating positive movement experiences, enjoyment of physical activity** | |
| --- | --- | --- |
| **Description** | | This category was applied when promoting positive movement experiences or the enjoyment of physical activity was mentioned. |
| **Reference example** | | *“The physiotherapist will encourage improved physical activities in a manner that gradually increases physical function and enhances enjoyment of physical activity.”* (Casey et al., 2018, p. 6) |

|  | **5.4 Tailoring / Individualization** | |
| --- | --- | --- |
| **Description** | | This category was applied when tailoring or individualizing any of the therapeutic components was mentioned. |
| **Reference example** | | *“Action plans were patient-centred and individuals supported to undertake PA of their choice to promote intrinsic motivation.”* (Cramp et al., 2020, p. 489) |

|  | **5.5 Consideration of interaction & language / Collaborative communication** | |
| --- | --- | --- |
| **Description** | | This category was applied when the interaction or communication between therapists and patients was specifically described, or when general communication to patients was discussed (e.g., preparation of learning materials in plain language). |
| **Reference example** | | *“Acts in a warm and caring way; Expresses empathy; Acknowledges and supports patients’ perspectives, feelings, and values; Avoids judgment or blame”* (Quinn et al., 2016, p. 76) |

|  | **5.6 Utilizing group settings** | |
| --- | --- | --- |
| **Description** | | This category was applied when a specific form of therapy was only feasible or more effective in a group setting, leading to a particular impact. |
| **Reference example** | | *“Group counselling can promote group cohesion, which can help motivate patients. Cohesion can be seen as a field of forces that act on the participants to stay in the group. These forces depend on participants being attracted to the group and the ability of the group to mediate important goals for the participants.”* (Helmink et al., 2010, p. 6) |
